# The lichen symbiosis re-viewed through the genomes of *Cladonia grayi* and its algal partner *Asterochloris glomerata*

**DOI:** 10.1186/s12864-019-5629-x

**Published:** 2019-07-23

**Authors:** Daniele Armaleo, Olaf Müller, François Lutzoni, Ólafur S. Andrésson, Guillaume Blanc, Helge B. Bode, Frank R. Collart, Francesco Dal Grande, Fred Dietrich, Igor V. Grigoriev, Suzanne Joneson, Alan Kuo, Peter E. Larsen, John M. Logsdon, David Lopez, Francis Martin, Susan P. May, Tami R. McDonald, Sabeeha S. Merchant, Vivian Miao, Emmanuelle Morin, Ryoko Oono, Matteo Pellegrini, Nimrod Rubinstein, Maria Virginia Sanchez-Puerta, Elizabeth Savelkoul, Imke Schmitt, Jason C. Slot, Darren Soanes, Péter Szövényi, Nicholas J. Talbot, Claire Veneault-Fourrey, Basil B. Xavier

**Affiliations:** 1 Department of Biology, Duke University, Durham, USA; 2 Department of Molecular Genetics and Microbiology, Duke University Medical Center, Durham, USA; 3 Faculty of Life and Environmental Sciences, University of Iceland, Reykjavík, Iceland; 4 Aix Marseille University, Université de Toulon, CNRS, IRD, MIO UM 110, 13288 Marseille, France; 5 Molekulare Biotechnologie, Fachbereich Biowissenschaften & Buchmann Institute for Molecular Life Sciences (BMLS), Goethe University Frankfurt, Frankfurt am Main, Germany; 6 Argonne National Laboratory, Biosciences Division, Argonne, & Department of Bioengineering, University of Illinois at Chicago, Chicago, USA; 7 Senckenberg Biodiversity and Climate Research Center (SBiK-F), Frankfurt am Main, Germany; 8 US Department of Energy Joint Genome Institute, Walnut Creek, USA; 9 Department of Plant and Microbial Biology, University of California – Berkeley, Berkeley, USA; 10 College of General Studies, University of Wisconsin - Milwaukee at Waukesha, Waukesha, USA; 11 Department of Biology, University of Iowa, Iowa City, USA; 12 Gilead Sciences, Inc., Foster City, USA; 13 INRA, Université de Lorraine, Interactions Arbres-Microorganismes, INRA-Nancy, Champenoux, France; 14 Department of Population Health and Pathobiology, College of Veterinary Medicine, North Carolina State University, Raleigh, USA; 15 Department of Biology, St. Catherine University, St. Paul, USA; 16 Department of Molecular and Cell Biology, University of California – Berkeley, Berkeley, USA; 17 Department of Microbiology and Immunology, University of British Columbia, Vancouver, Canada; 18 Department of Ecology, Evolution, and Marine Biology, University of California - Santa Barbara, Santa Barbara, USA; 19 Department of Molecular, Cell, and Developmental Biology, and DOE Institute for Genomics and Proteomics, University of California, Los Angeles, USA; 20 National Evolutionary Synthesis Center, Durham, USA; 21 Calico Life Sciences LLC, South San Francisco, USA; 22 IBAM, Facultad de Ciencias Agrarias, CONICET, Universidad Nacional de Cuyo, Chacras de Coria, Argentina; 23 Institute of Ecology, Evolution and Diversity, Fachbereich Biowissenschaften, Goethe University Frankfurt, Frankfurt am Main, Germany; 24 College of Food, Agricultural, and Environmental Sciences, Department of Plant Pathology, The Ohio State University, Columbus, USA; 25 College of Life & Environmental Sciences, University of Exeter, Exeter, UK; 26 Department of Systematic and Evolutionary Botany, University of Zurich, Zurich, Switzerland; 27 The Sainsbury Laboratory, Norwich Research Park, Norwich, UK; 28 Université de Lorraine, INRA, Interactions Arbres-Microorganismes, Faculté des Sciences et Technologies, Vandoeuvre les Nancy Cedex, France; 29 Laboratory of Medical Microbiology, Vaccine & Infectious Disease Institute, University of Antwerp, Antwerp, Belgium

**Keywords:** Algal virus, Coculture, Fungi, Gene expression, Gene family evolution, Horizontal gene transfer, Plant-fungal interactions, Symbiont autonomy, Symbiosis genes

## Abstract

**Background:**

Lichens, encompassing 20,000 known species, are symbioses between specialized fungi (mycobionts), mostly ascomycetes, and unicellular green algae or cyanobacteria (photobionts). Here we describe the first parallel genomic analysis of the mycobiont *Cladonia grayi* and of its green algal photobiont *Asterochloris glomerata*. We focus on genes/predicted proteins of potential symbiotic significance, sought by surveying proteins differentially activated during early stages of mycobiont and photobiont interaction in coculture, expanded or contracted protein families, and proteins with differential rates of evolution.

**Results:**

A) In coculture, the fungus upregulated small secreted proteins, membrane transport proteins, signal transduction components, extracellular hydrolases and, notably, a ribitol transporter and an ammonium transporter, and the alga activated DNA metabolism, signal transduction, and expression of flagellar components. B) Expanded fungal protein families include heterokaryon incompatibility proteins, polyketide synthases, and a unique set of G-protein α subunit paralogs. Expanded algal protein families include carbohydrate active enzymes and a specific subclass of cytoplasmic carbonic anhydrases. The alga also appears to have acquired by horizontal gene transfer from prokaryotes novel archaeal ATPases and Desiccation-Related Proteins. Expanded in both symbionts are signal transduction components, ankyrin domain proteins and transcription factors involved in chromatin remodeling and stress responses. The fungal transportome is contracted, as are algal nitrate assimilation genes. C) In the mycobiont, slow-evolving proteins were enriched for components involved in protein translation, translocation and sorting.

**Conclusions:**

The surveyed genes affect stress resistance, signaling, genome reprogramming, nutritional and structural interactions. The alga carries many genes likely transferred horizontally through viruses, yet we found no evidence of inter-symbiont gene transfer. The presence in the photobiont of meiosis-specific genes supports the notion that sexual reproduction occurs in *Asterochloris* while they are free-living, a phenomenon with implications for the adaptability of lichens and the persistent autonomy of the symbionts. The diversity of the genes affecting the symbiosis suggests that lichens evolved by accretion of many scattered regulatory and structural changes rather than through introduction of a few key innovations. This predicts that paths to lichenization were variable in different phyla, which is consistent with the emerging consensus that ascolichens could have had a few independent origins.

**Electronic supplementary material:**

The online version of this article (10.1186/s12864-019-5629-x) contains supplementary material, which is available to authorized users.

## Background

Simon Schwendener in 1869 [1] correctly recognized lichens as intimate symbioses between specialized fungi and phototrophic unicellular green algae as the main symbionts. Cyanobacteria were later recognized also as primary phototrophs in many lichens. In addition to the main symbiotic partners, lichens harbor diverse communities of prokaryotes and fungi as cohabitants [2–6]. Recently, highly coevolved basidiomycete yeasts were discovered in the cortex of many lichens [7], sometimes causing disease [8]. The detailed interactions of the various cohabitants with the main symbionts are being investigated [9]. Typically, lichens thrive in above-ground niches with limited water in diverse environments, often withstanding extreme heat, desiccation, or cold [3, 10]. Widespread across terrestrial ecosystems, often dominant carbon and nitrogen fixers in alpine, subalpine, and high latitude habitats, the estimated 18,000 to 20,000 lichen species [11], mostly ascomycetes, represent about 20% of all known fungi [12]. There are only about 120 lichen phototroph species (photobionts) [10, 13], far fewer than the 20,000 known lichen fungal species (mycobionts). Lichens are named based on their mycobiont since the fungus is the most conspicuous partner and since the same photobiont species (alga or cyanobacterium) can be found in several different lichens.

Lichens can reproduce somatically through propagules comprising both symbionts, or sexually through meiotic fungal spores that must combine with the appropriate photobiont to re-form a lichen. Sexual reproduction is not commonly seen in trebouxoid lichen algae, although evidence supporting it has been found (Heterothallism probably evolved from homothallism in Cladonia; genetic evidence for sex in Asterochloris section). Lichens are well known for their unique and abundantly produced secondary metabolites [14, 15]. The genetic, physiological, and structural integration of mycobionts and photobionts has produced a vast array of beautifully differentiated partnerships [16], with only occasional instability [17–19]. The fungal and lichen fossil record [20, 21] has placed fossils resembling extant lichen taxa in the Devonian-early Carboniferous, 415–350 million years ago, and perhaps simpler mycobiont-photobiont associations even earlier [22, 23]. Despite their intimate coexistence for hundreds of millions of years and the construction of complex interfaces between them [10, 24], lichen symbionts have not lost their genetic and cellular independence. Cell membranes are not breached in their interactions and genomes are not merged, although some have extended to lichens the concept of genome acquisition [25]. There is no evidence of horizontal gene transfer (HGT) between the symbionts, yet both have acquired genes from other sources (A low-GC region in Asterochloris is a remnant of a large virus insertion, an HGT-mediator, Inferences from differential transcription about nutritional fluxes at the symbiotic interface, and Photobiont expanded families sections). The partners of many lichens have been isolated and grown separately in axenic culture, but free-living stages of lichen fungi and their algae remain mostly cryptic in nature [18, 26–29]. However, these stages are not insignificant for the lichen life cycle [29]. The laboratory reconstitution of cultured lichen symbionts into fully developed lichens has a checkered history [30], where the reproducibility needed for molecular investigations is still elusive. Molecular studies addressing functional aspects of this mutualistic symbiosis are few [9, 31–35], but the recent publication of several lichen -omics papers and datasets [9, 36–46] heralds expansion of this field.

**Fig. 1:**
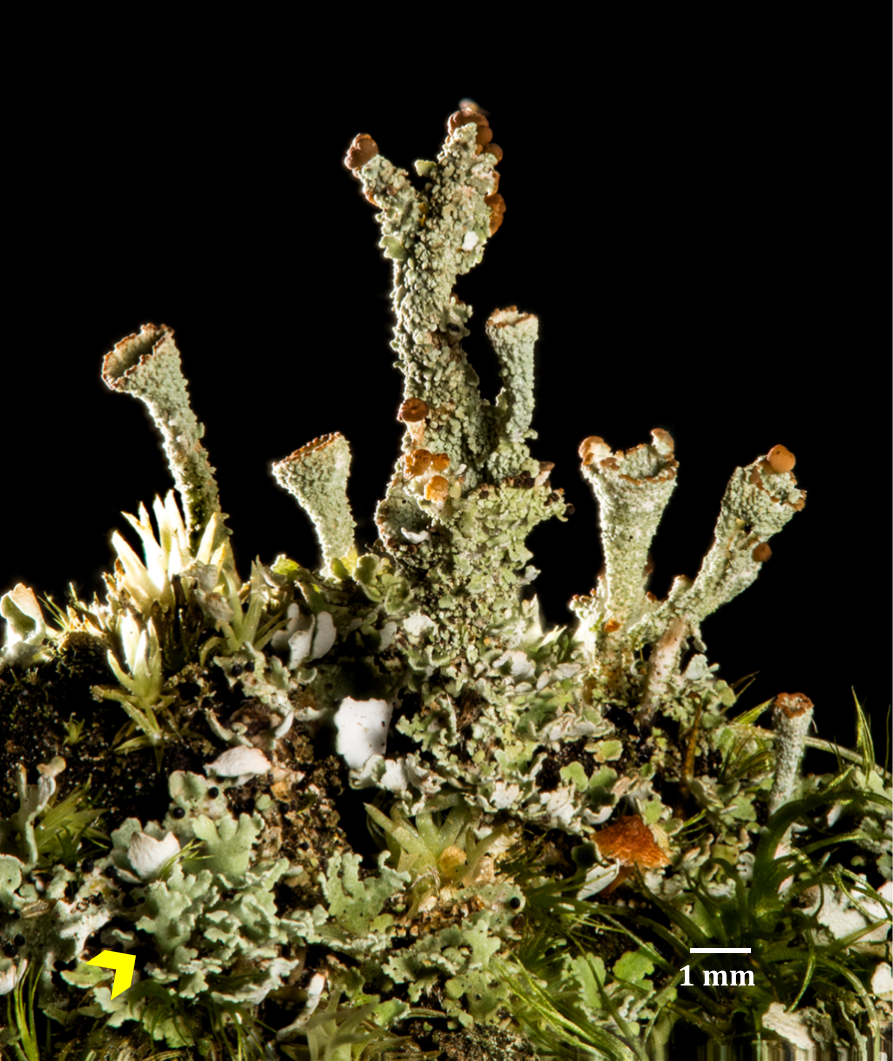
The lichen *Cladonia grayi*. The most conspicuous parts of the *Cladonia* thallus are the goblet-shaped podetia that support the sexual and vegetative reproductive structures: the goblets’ upper margins are covered with brown fungal apothecia, sites of meiotic spore production and ejection into the air; the podetial surfaces are covered with green vegetative propagules called soredia, which are tiny alga-fungus packets detached by rain and wind and able to grow and differentiate into full thalli. Soredia are continuously produced and extruded onto the podetial surface from the underlying fungal tissue, which has algae embedded in it. The ground is covered with the less conspicuous, leaf-shaped parts of *Cladonia* called squamules (yellow arrowhead), which are tiny but fully differentiated lichen thalli with typical medullar, algal, and cortical layers. The grass-like bodies are bryophyte initials. The focus-stacked photograph was taken in D.A.’s lab by Thomas Barlow, who holds the copyright and consents to its use in this study

Here we present the first parallel genomic analysis of both primary symbionts in a lichen, the fungus (mycobiont) *Cladonia grayi* and the alga (photobiont) *Asterochloris glomerata*, and use several approaches to identify genes/proteins of potential symbiotic relevance. Our analysis is based exclusively on the symbionts’ nucleic acid sequences, and the proteins involved are predicted. *Cladonia grayi* (Fig. 1) belongs to a genus with worldwide distribution, part of the class of Lecanoromycetes that includes 70% of the known lichens [47] (phylum Ascomycota, subphylum Pezizomycotina). The unicellular photobiont, *A. glomerata*, belongs to the most common order of lichen algae, the Trebouxiales [13, 48]*.* There are few sequenced genomes from unicellular chlorophyte algae [49–55]: some are naturally free-living and some, like *Coccomyxa subellipsoidea* [54] and *Chlorella variabilis*, are facultative symbionts [50, 54, 56]. Genomic analysis of lichens will not only increase the molecular and ecological understanding of a large and understudied portion of the fungal and algal phyla but also complement the emerging genomics of other symbioses involving mycorrhizal [57–60], endophytic [61, 62], or plant pathogenic fungi [63].

## Results and discussion

### General characteristics of the *C. grayi* and *A. glomerata* genomes

#### Genome sizes and gene organization

**Fig. 2:**
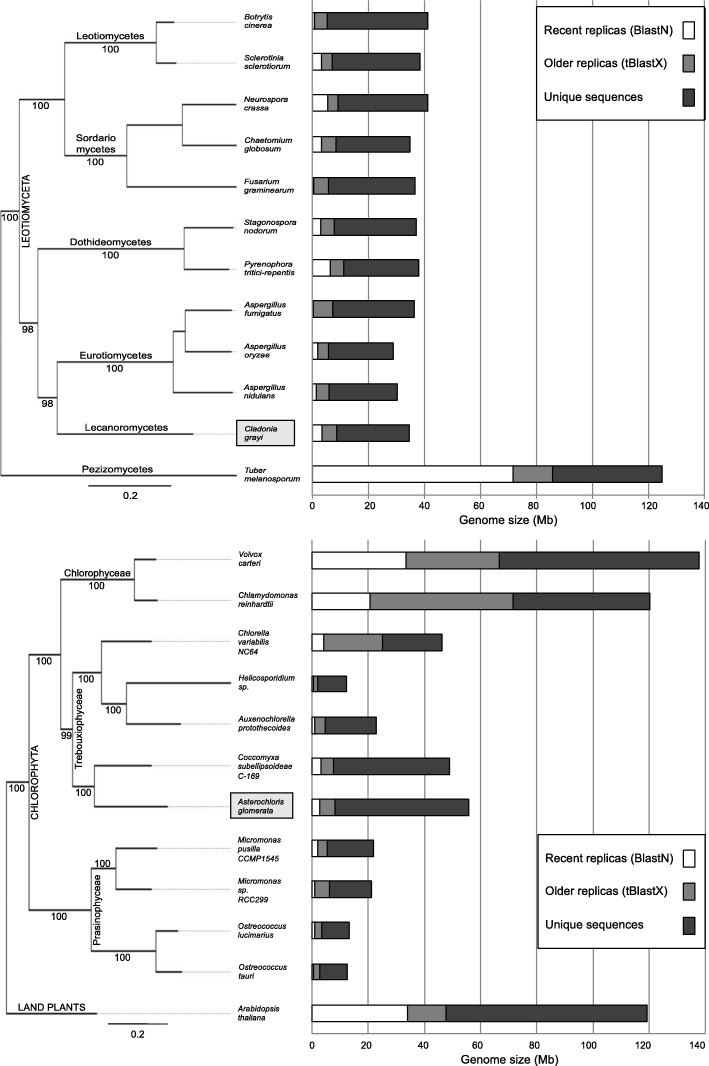
Phylogenies, genome sizes and sequence distribution. *Left side*: Fungal (top) and algal (bottom) PhyML trees (LG + G + F + I) for *C. grayi* and *A. glomerata* involving, respectively, a random sample of 6000 and 4000 ungapped sites extracted from a concatenated alignment of 2137 and 683 orthologous protein families containing 794,828 and 159,356 ungapped sites. Bootstrap support values label internodes. Scales indicate nucleotide substitutions per site. *Right side*: Bars are proportional to genome size, and different shadings indicate the proportions of recent and older sequence replicas or of unique sequences. Duplicated sequences in genomes were revealed by BLAST alignment of the genomic sequence against itself at the nucleotide (BLASTN) or amino acid (TBLASTX) levels. The duplicated regions include regular genes as well as repeated elements (not yet fully characterized), but microsatellites and low complexity sequences were filtered out. Sequences that matched in both BLASTN and TBLASTX searches were only counted in the BLASTN category. Only alignments with e-values <1e^− 15^ in both the BLASTN and TBLASTX analyses were considered

The mycobiont is a single-spore isolate from *C. grayi* and the photobiont is *Asterochloris glomerata* isolated from *C. grayi* soredia [64]. Table 1 includes basic features of the two nuclear and the three organelle genomes. Organelle genomes are briefly discussed in Additional file 1, and details are in Xavier et al. [65] and Xavier’s thesis [66]. Whole genome assemblies and annotations are at [67] for the mycobiont and at [68] for the photobiont. Relationships of *C. grayi* and *A.glomerata* within broad phylogenetic contexts, genome sizes, and proportions of repeated and unique sequences are shown in Fig. 2. The nuclear genomes of lichen symbionts are not reduced in size nor gene content compared to free-living relatives, in contrast to the reductions observed in many host-dependent bacteria [69]. With its 35 Mb genome and 11,400 gene models, the *C. grayi* mycobiont falls in the average size range for most Ascomycota [70]. Other lichen fungi fall in the same range, between 26 and 59 Mb [46]. Large increases in the number of transposable elements significantly affect genome size in many biotrophic fungi [71], including the ectomycorrhizal ascomycetes *T. melanosporum* [58], *E. granulatus* [72], and *C. geophilum* [73] but this was not observed in *C. grayi* (Fig. 2). Like other Chlorophyta, *A. glomerata* has more and larger introns than fungi. Its genome (56 Mb and 10,000 gene models) is significantly smaller than that of *C. reinhardtii* (120 Mb) [49] but is larger than that of other Trebouxiophyceae like *C. subellipsoideae* C-169 (49 Mb) [54] and *C. variabilis* NC64A (46.2 Mb) [50] (Fig. 2). *C. subellipsoideae* C-169 is free-living, but the genus includes lichenized species [56, 74]. *Chlorella* NC64A is a facultative symbiont of ciliates and is host to large dsDNA viruses. Our analyses suggest that *A. glomerata* has also been host to large DNA viruses (A low-GC region in Asterochloris is a remnant of a large virus insertion, an HGT-mediator section), although a live virus has not yet been isolated from it. Over evolutionary time, chromosomal rearrangements left little synteny among the genomes of *A. glomerata*, *Coccomyxa* C-169 and *Chlorella* NC64A (Additional file 2).

**Table 1:** Genome Basics

Nuclear Genome	*Cladonia grayi*		*Asterochloris glomerata*	
Coverage	15x		24.8 x	
Number of scaffolds	414		151	
Genome size (Mb)	35		56	
Number of predicted genes	11,388		10,025	
Number expressed in thallus	9800 (86%)		7700 (77%)	
Genes per million bases	288		173	
Average # of introns per gene	3		9	
Average gene (mRNA) length	1800 (1650)		4240 (1400)	
Intergenic DNA	~ 45%		~ 26%	
Repetitive DNA	~ 10%		~ 5%	
Organelle Genomes	Basepairs	Proteins	Unknown ORFs	tRNAs
Fungal mitochondrion	50,836	15	1	26
Algal mitochondrion	11,0932	32	18	25
Chloroplast	217,546	73	1	30

Additional file 3 shows a KEGG-based categorization of the mycobiont and photobiont gene models. In this broad overview only the environmental information processing category (signal transduction) appears overrepresented in both symbiotic partners. Among the free-living *Aspergillus* species, from the closely related class Eurotiomycetes (Fig. 2), the signal transduction genes constitute between 1.4 and 1.84% of the annotated genes [75, 76], while in *C. grayi* the proportion is 6.2%. In *A. glomerata* the proportion of signal transduction genes is 7.8%, while among other Chlorophyta they represent 5–6% of the total [77]. These broad comparisons are only suggestive of an expansion of signal transduction components in the *C. grayi* partners because methodologies and annotations differ. A specific analysis of signal transduction functions (Specific survey of mycobiont and photobiont signal transduction components section) also reveals diversification in some of the *C. grayi* and *A. glomerata* components. This bilateral restructuring may underpin the multifaceted interactions between partners [10].

#### A low-GC region in *Asterochloris* is a remnant of a large virus insertion, an HGT-mediator

**Fig. 3:**
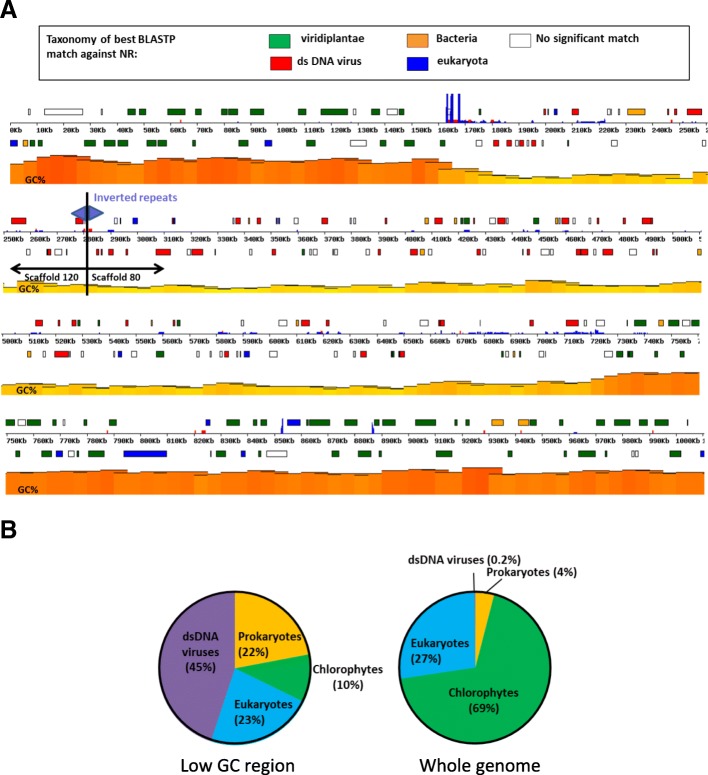
A viral insertion in the *Asterochloris* genome. **a** GC content and gene distribution. The diagram represents a 1 Mb genomic region produced by joining scaffolds 120 and 80 at their inverted repeat-containing edges (purple triangles). The % GC content is proportional to the height and color intensity of the orange-yellow band. Genes are indicated by rectangles whose color represents the category of their best match in Genbank (BLASTP e-value <1e^− 5^). The blue or red segments perpendicular to the Kb line are repeated sequences or gaps, respectively. The low GC region in yellow represents the remnant of a viral insertion (Additional file 4). **b** Origins of best matches. Most genes in the low GC region of *A. glomerata* are viral or prokaryotic in origin, in contrast to those in the genome as a whole

In 98% of the *Asterochloris* nuclear genome the GC content is between 56 and 62%. However, it is significantly lower (49%) in two large genomic regions, each located at one end of two scaffolds (~ 441 Kb on scaffold 80 and ~ 102 Kb on scaffold 120). Each low-GC region reaches the scaffold’s extremity with an array of duplicated 141-bp sequence units (totaling 1300 bp on scaffold 80 and 2053 bp on scaffold 120). These repeated sequences are found nowhere else in the *Asterochloris* genome. Figure 3a has the two scaffolds joined at the repeats, forming a single low-GC contiguous chromosomal region. Genomic contiguity has been confirmed by PCR and sequencing across the junction (Armaleo, not shown). The joined low-GC regions contain 462 predicted protein coding genes (Additional file 4), 236 of which exhibit significant matches in GenBank (BLASTP e-value <1e^− 5^). Of these, 45% have their best match in double-stranded DNA viruses [78]. While most genes in the algal genome have many introns, only 36 of the 462 protein coding genes in this region are predicted to have them, and only 24 of 462 (5.2%) match chlorophyte genes. This differs markedly from the rest of the genome (Fig. 3b), where most genes have best matches in chlorophytes (69%). The sharp switch in nucleotide composition and phylogenetic affinity strongly suggest that the low-GC region is a remnant of a large integrated viral genome, about 540 kb long. Nucleo-cytoplasmic large DNA viruses (NCLDV) form a monophyletic class of viruses that infect a variety of eukaryotes [78, 79], including other algae and protists [80–82]. A phylogenetic analysis places the *Asterochloris* virus within the *Phycodnaviridae* family, sister to viruses that infect other green algae (Additional file 4). The genome of the *A. glomerata* virus may be the largest among alga-infecting NCLDVs sequenced to date (ranging from 154 Kb for a *Feldmannia* sp. virus [83] to 473 Kb for a *Chrysochromulina ericina* virus [84]). Viral DNA thus is a major vehicle of HGT in *A. glomerata* and other algae [85]. The significance of this group of virally transferred genes for the symbiosis is unclear at this time. However, some of the genes in the viral region are actively transcribed, which suggests that they may eventually become functional in the photobiont. Other genes with potential symbiotic significance have been introduced into trebouxioid algae probably by HGT from bacteria [44] and archaea (Photobiont expanded families section). Trebouxioid algae ancestors may have even acquired genes from fungi before the origins of lichens [86].

#### Heterothallism probably evolved from homothallism in *Cladonia*; genetic evidence for sex in *Asterochloris*

**Fig. 4:**
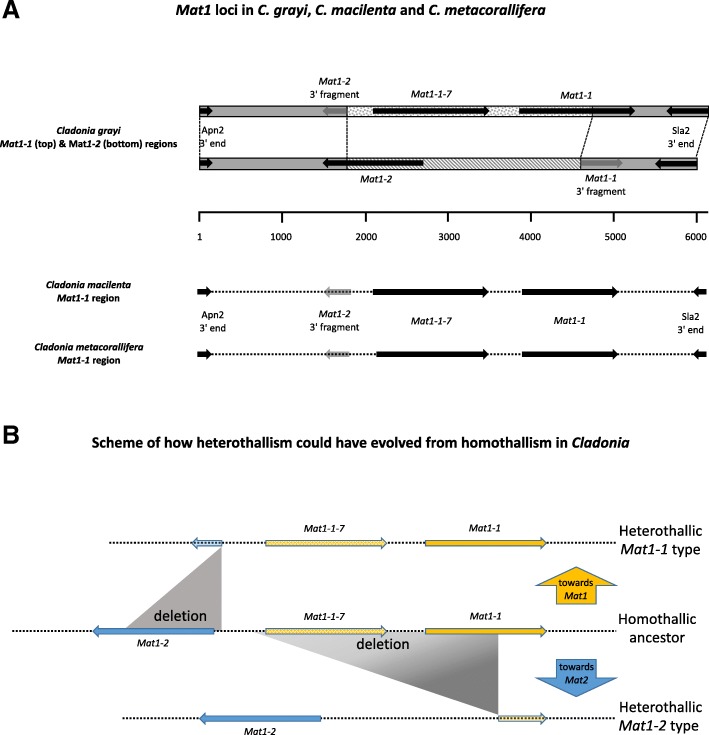
*Cladonia* MAT loci and their evolution. **a** Configurations of the MAT loci in three *Cladonia* species. The top diagrammed alignment is based on the alignment between the annotated *C. grayi MAT1–1* region (scaffold_00075:76000–90,000 at [67]) and a provisional sequence of the *C. grayi MAT1–2* region (accession MH795990). The *C. grayi MAT1–1* and *MAT1–2* regions are drawn above the basepair indicator line. Under the line are the *MAT1–1* regions derived from the genomes of two other *Cladonia* species (Additional file 5). In *C. grayi*, the conserved flanking regions are gray, while the unrelated central regions are stippled differently for each mating type. Dark or gray arrows represent genes and gene-segments. CLAGR_008123-RA is considered a putative *MAT1–1-7* ortholog because of its location and its BLAST hits to *MAT1–1-7* orthologs from *Trichophyton* and other fungi. **b** Evolutionary model. Horizontal colored arrows represent *MAT* idiomorphs. The central line represents the MAT locus configuration of a possible homothallic *Cladonia* ancestor, and the vertical arrows represent the putative transitions towards the present heterothallic *MAT1–1* (orange) or *MAT1–2* (blue) configurations (Additional file 5). The graded shading in the deletion triangle leading to *MAT1–2* symbolizes the deletion’s undefined left boundary beyond *MAT1–1-7*

Typically in ascomycetes, two kinds of mating genes, *MAT1–1* and *MAT1–2*, cooperate in mating [87]. They are referred to as idiomorphs because, while they share the same locus, *MAT1*, their encoded proteins are different transcription factors: *MAT1–1* is characterized by an α1-domain [88] and *MAT1–2* by a MAT A_ HMG domain [88]. These may be linked also to other idiomorph-specific genes. When both *MAT1–1* and *MAT1–2* are in the same genome, the fungus is self-fertile (homothallic), but when a genome contains only *MAT1–1* or only *MAT1–2*, the fungus is self-sterile (heterothallic) and mating occurs only between different mating types; both heterothallic and homothallic species of lichen fungi have been found [89–93]. *C. grayi* produces typical ascomycetous fruiting bodies with apothecia (Fig. 1); the *MAT* locus in the single-spore isolate *Cgr/DA2myc/ss*, like in many Pezizomycota [87, 94], is located between *Apn2* and *Sla2*, genes for a nuclease and a cytoskeleton assembly protein, respectively (Fig. 4a). The locus in *Cgr/DA2myc/ss* contains the core gene *MAT1–1* and an associated gene [87], *MAT1–1-7*, but no *MAT1–2* (Additional file 5 and Fig. 4a), as do the sequenced mating-type loci of single-spore isolates from two other *Cladonia* species (Additional file 5). This expands earlier RAPD-PCR and RFLP data on three other *Cladonia* species, also heterothallic [91]. PCR amplification and sequencing of the region between *Sla2* and *Apn2* from DNA of a natural *C. grayi* thallus revealed a single *MAT1–2* gene (Additional file 5 and Fig. 4a; sequence accession MH795990), further supporting heterothallism. The sequences of the two loci revealed the presence of vestigial sequences suggesting that, in *Cladonia*, heterothallism might have evolved from homothallism (Additional file 5 and Fig. 4b). This appears to diverge from the trends in other ascomycete genera where homothallism is thought to have evolved from heterothallism [94–96], although not in all cases [97].

**Fig. 5:**
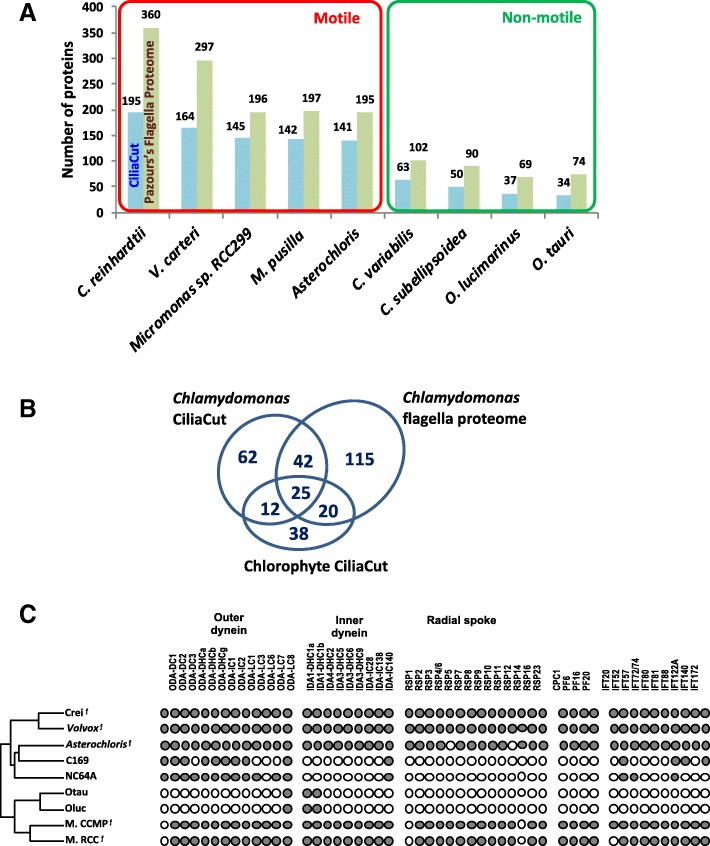
Flagellar proteins. **a** Number of candidate flagella proteins in chlorophytes. Reference *C. reinhardtii* proteins of the CiliaCut protein set (blue bars) and flagella proteome (green bars) were searched for putative orthologs in sequenced motile and non-motile chlorophytes using the reciprocal best BLASTP hit criterion. **b** The 314 candidate *A. glomerata* flagella proteins identified from multiple sources of evidence (see Methods). **c** Distribution of flagella proteins across Chlorophytes. The left cladogram shows the likely evolutionary relationships of sequenced Chlorophytes. The ƒ mark indicates organisms known to build motile flagella. Crei: *Chlamydomonas reinhardtii*; Volvox: *Volvox carteri*; C169: *Coccomyxa subellipsoidea* C-169; NC64A: *Chlorella variabilis* NC64A; Otau: *Ostreococcus tauri*; Oluc: *Ostreococcus lucimarinus*; M. CCMP: *Micromonas pusilla CCMP1545*; M. RCC: *Micromonas* sp. RC299. Presence (dot) or absence (circle) of putative orthologs identified by reciprocal best BLASTP hit of *C. reinhardtii* outer dynein proteins, inner dynein proteins, radial spoke proteins, central pair proteins and intraflagellar transport proteins

Sexual reproduction is generally assumed not to occur in trebouxoid algae like *Asterochloris* [98]. They reproduce vegetatively, both in the lichen thallus or in culture, through non-motile autospores and aplanospores or through flagellated zoospores [99]. Law and Lewis proposed that sexual reproduction in the photobiont, the inhabitant in the mutualism, should be selected against [100]. However, the occurrence of sex in these algae is indicated by isolated microscopic observation of presumptive gametes in the 1920s [99], 1960s (figure 28 [101]; page 135 and figure 28 [102]) and in 2015 [99], and molecular evidence of genetic recombination was also uncovered through phylogenetic analysis of a *Trebouxia* population in *Letharia* lichens [103]. The identification of many meiotic genes in the *A. glomerata* genome and their expression in coculture (Additional file 5) add further evidence for the occurrence of meiosis in trebouxoid algae, probably in their free-living stages [29]. The gametes observed in *Asterochloris* [99] are flagellated, as are its vegetative zoospores (9 + 2 type [104]). Not surprisingly, the motility proteins present in *Asterochloris* match those of other flagellated chlorophytes but are mostly absent from non-motile chlorophytes (Fig. 5 and Additional file 5). The critical implications of fungal and algal sexual reproduction for the lichen symbiosis and for symbiont autonomy are discussed in Conclusions.

### Search for symbiosis-specific genes I: differential expression in coculture vs. monoculture

**Fig. 6:**
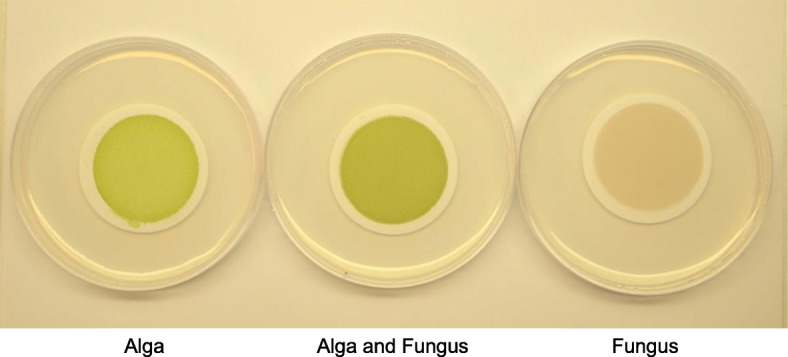
Cultures of *C. grayi* and *A. glomerata* reconstituted on filters in Petri dishes

Table 2 lists the genes discussed in the next three sections. The system to survey the gene sets differentially transcribed when fungus and alga first contact each other is based on three parallel cultures on filters placed on low nutrient agar medium [33]: the aposymbiotic fungus, the aposymbiotic alga, and a coculture of the two (Fig. 6 and Additional file 6). RNA was extracted from the individual cultures after 21 days of growth on the filters. Differential expression data (Methods) were analyzed in two ways. In the first, we identified GO annotations and metabolic pathways significantly enriched among genes induced or repressed more than 1.5x (0.6 > log_2_ > − 0.6) in coculture relative to monoculture. In the second, a more extensive approach, we focused only on induced genes and added information from PFAM domains and literature searches to define each gene’s putative function in more detail than was possible through GO terms alone.

**Table 2:** Compilation of genes/proteins of potential symbiotic significance discussed in Search for symbiosis-specific genes I, II, and III sections

Fungus: *Cladonia grayi*	
Gene groups induced in coculture	Small secreted proteins / Transcription / Cell wall turnover / Protein turnover / Metabolism / Membrane transport / Defense / Extracellular hydrolases
Selected examples	Polyol transporter / Ammonium transporter / Calcium channel inhibitor / lectins / DNA methyltransferase / Gα, RGS protein, dual specificity phosphatase
Expanded protein families	HET domain / Ank domain / Met permeases / Unknown transmembrane proteins / Fructosamine kinases / Polyketide synthases / Signal transduction components / Stress-related TFs
Contracted protein families	*Transportome*: Carbohydrate transporters / Major Facilitator Superfamily (MFS) / ATP Binding Cassette (ABC) superfamily / Aminoacid-Polyamine organo Cation (APC) family / Oligopeptide Transporter (OPT) family / Proton-dependent Oligopeptide Transporter (POT) family
Slow evolvers	Proteostasis maintenance / Aldehyde dehydrogenases / Major Facilitator Superfamily (MFS)
Selected examples	Mechanosensitive calcium channel / Sugar transporters
Fast evolvers	Signal transduction / Membrane trafficking / Stress protection
Selected examples	Superoxide dismutase / Trehalose synthase
Alga: *Asterochloris glomerata*	
Gene groups induced in co-culture	Secreted proteins / Transcription / Cell wall turnover / Protein turnover - ubiquitin / DNA processes / Signal transduction / Protein trafficking / Flagellum synthesis
Selected examples	Thioredoxin / Kinesin / Fasciclin domain proteins / Mechanosensitive calcium channel
Expanded/new protein families	Fam_16: DNA binding-recombination proteins / Kinases / Carbohydrate active enzymes (CAZ) / Ank domain proteins / Archaeal ATPases / Desiccation-Related-Proteins / Magnesium transporters / Signal transduction components / Stress-related TFs
Contracted protein families	Nitrate assimilation
Slow evolvers	Two kinases and one clathrin vesicle adaptor
Fast evolvers	Seven diverse proteins

#### GO term-centered analysis of transcription in coculture

The limited results are listed in Additional file 6. In coculture, the alga shows only differential activation of ubiquitin-dependent catabolic processes. The fungus induces redox-active enzymes and proteases, a finding also reflected in the data from the more extensive approach. Surprisingly, however, in presence of the alga the fungus also down-regulates several genes involved in respiratory ATP generation; the biological significance of this remains to be determined, pending experimental confirmation.

#### Extended analysis of transcription induced in coculture

**Fig. 7:**
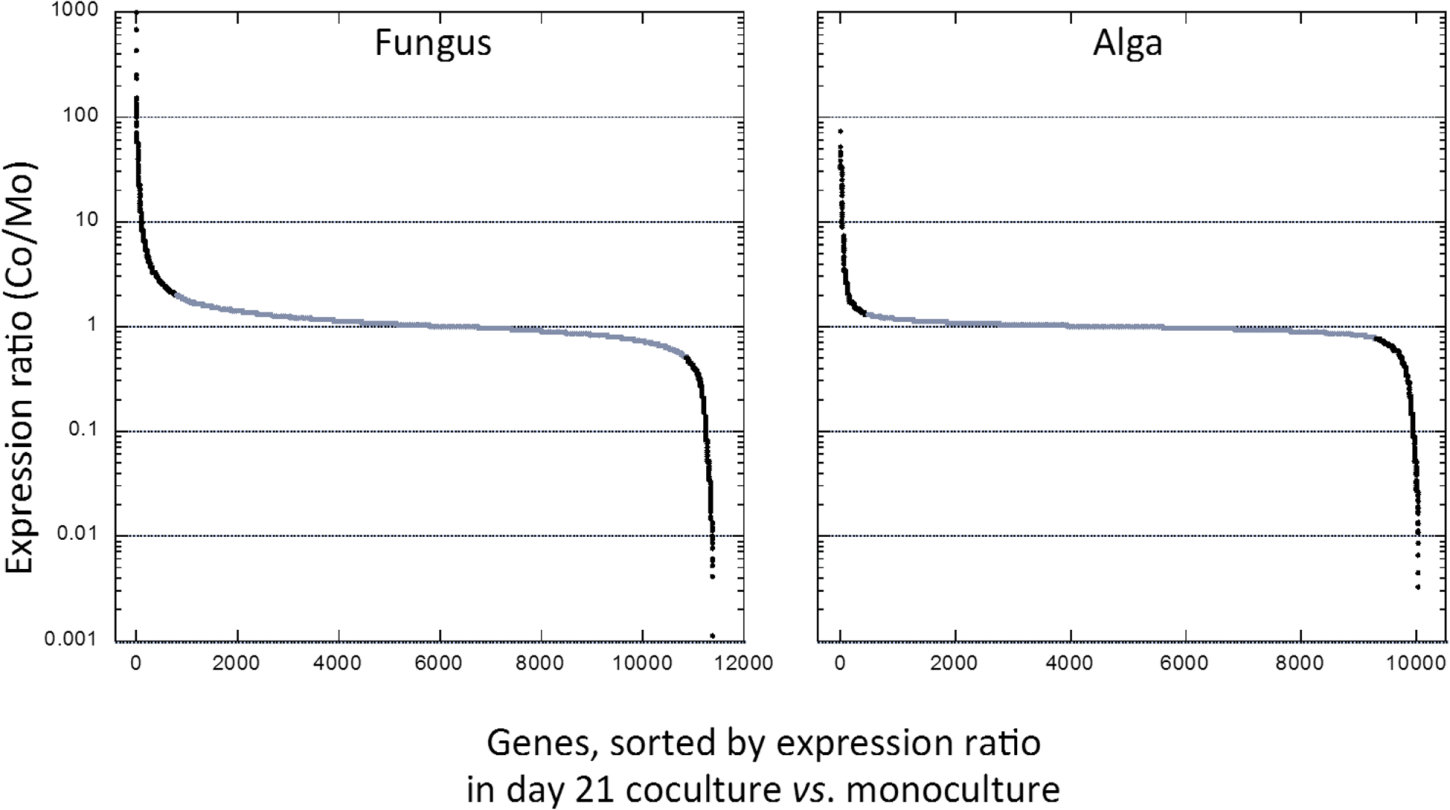
Differential fungal (*C. grayi*) and algal (*A. glomerata*) gene expression in coculture vs. monoculture. RPKM expression ratios are sorted from high to low. Genes considered unaffected in coculture are labeled gray (Co/Mo ~ 1). Those labeled black above or below the gray range are considered induced or repressed, respectively. Induction and repression thresholds correspond respectively to 2 and 0.5 for the fungus and 1.3 and 0.77 for the alga. Notice the smaller range of differential expression induction displayed by the alga under our experimental conditions (Additional file 6)

**Fig. 8:**
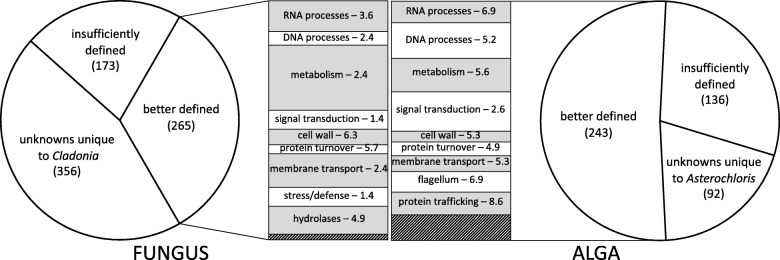
Classes of genes differentially induced during early fungus-alga interactions in coculture. The pie charts divide the induced genes for each symbiont into three broad classes (numbers of genes in parentheses). The “better defined” genes are subdivided in groups roughly comparable between the symbionts (gray and white boxes). The area of each box is proportional to the percent of genes it contains relative to all better defined genes (265 for the fungus and 243 for the alga). The number behind each group’s name indicates its enrichment factor relative to the whole genome (see Methods). The hatched areas represent groups with less than 10 genes each. The *p* values for the enrichment of the indicated groups within the induced genes are all < 0.05, and most are << 10^− 3^

In this analysis we used day-21 ratios of coculture (Co) vs. monoculture (Mo) RPKM directly rather than the log_2_ values. Ratios are abbreviated here as Co/Mo. In each symbiont, a few hundred genes changed expression in coculture relative to monoculture, while most genes remained unaffected (Co/Mo ≅ 1) (Fig. 7). The extended transcription analysis was limited to the genes induced in coculture. Due to differences in Co/Mo ranges for the symbionts (Additional file 6), we defined two different Co/Mo induction thresholds: ≥ 2 for the fungus, ≥1.3 for the alga (Fig. 7). This yielded 795 up-regulated genes out of 11,388 in *C. grayi* (7%) and 471 out of 10,024 in *A. glomerata* (4.7%) (Additional file 6). Induced gene products were inferred by BLAST [105] and, based on GO terms, PFAM domains and literature searches, were grouped into three categories summarized in Fig. 8: unknown and unique to each symbiont, insufficiently defined, and better defined (Additional file 6).

**Fig. 9:**
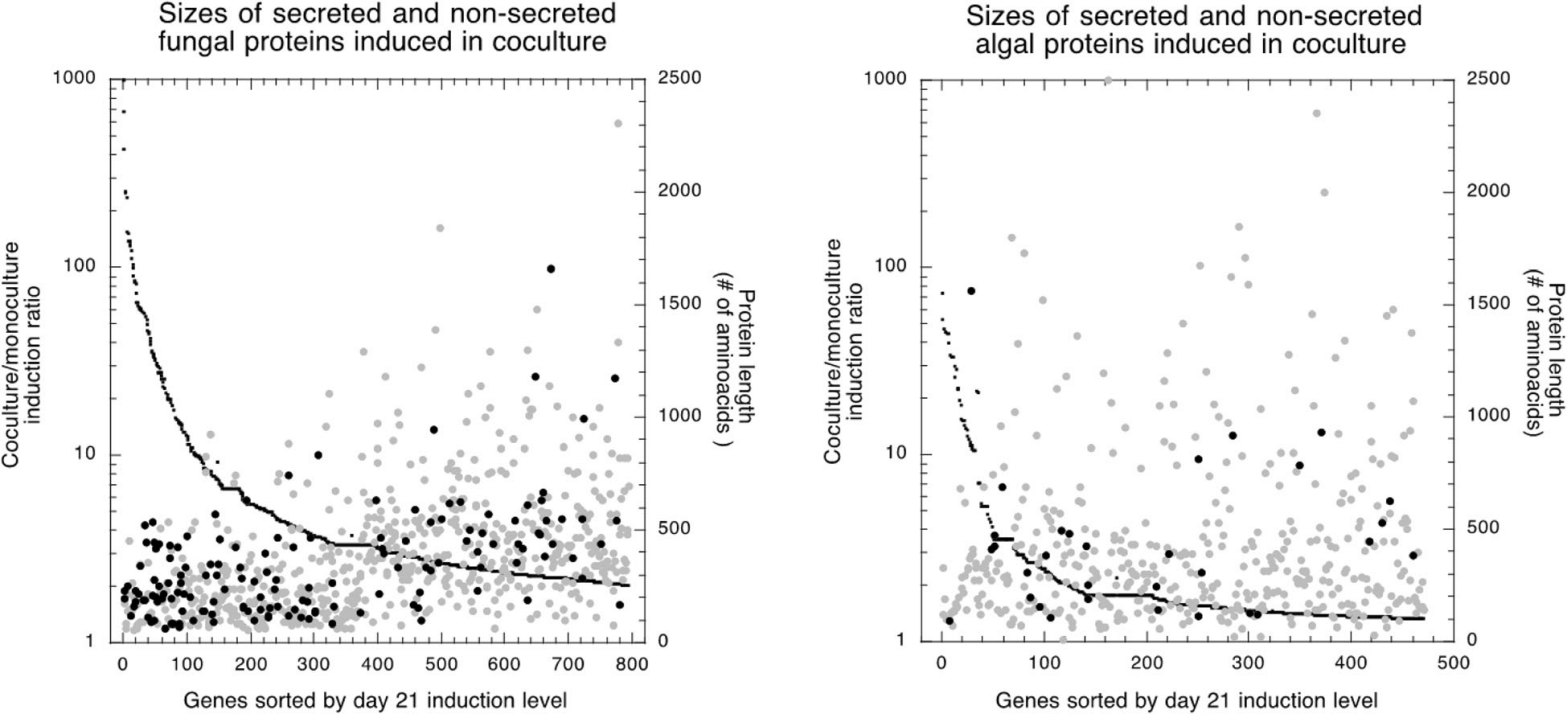
Secreted proteins among the proteins induced in coculture. The small black symbols coalescing into a curve represent the Co/Mo ratios of the genes induced in coculture; the circles represent the corresponding protein sizes, gray for non-secreted, black for secreted proteins. The average sizes (# of amino acids) of a) all genome proteins, b) all induced proteins, c) all induced and secreted proteins are a) 477, b) 381, c) 341 for the fungus and a) 447, b) 420, c) 436 for the alga. Proteins were considered secreted only if they scored as such in all three programs SignalP [109], TargetP [110], and TMHMM [111]. In TMHMM, a transmembrane domain prediction program, a protein was considered compatible with secretion only if it had either no predicted TM domains or only one at the N terminus. The data used in this figure are in Additional file 6

Relative to their overall genomic frequency of 7.2% (821/11388), mycobiont secreted proteins are disproportionately enriched to 18.5% (147/795) in coculture (*p* = 2.8E-28). The genomic average protein length is 477 AA, while the induced and secreted proteins are smaller, averaging 341 AA (Fig. 9 and Additional file 6), with most unknown and unique to *C. grayi*. The smallest proteins tend to be also among the most strongly induced. *Cladonia* shares biotrophic overexpression of small secreted proteins with the mycorrhizal basidiomycete *Laccaria bicolor* [57], the mycorrhizal ascomycete *Tuber melanosporum* [58], and with pathogenic fungi [106]. The *Cladonia* secretome (821 proteins) is comparable in size to the secretomes of ectomycorrhizal and many other fungi [107]. Also in the alga, relative to their overall genomic frequency of 3.6% (365/10025), secreted proteins appear significantly enriched (*p* = 0.00003) among the 471 induced proteins (32/471 = 6.8%), but they are not significantly smaller than average (Fig. 9 and Additional file 6). It remains to be seen whether the algal secretome in early symbiosis shares any features with the secretion responses of higher plants to fungal infection [108].

The better-defined gene products induced in coculture, 265 in *C. grayi* and 243 in *A. glomerata*, were subdivided for enrichment analysis in broad functional subgroups of at least 10 genes each (Fig. 8, Additional file 6). Except for the limited enrichment (1.4-fold) of signal transduction and defense genes in the fungus, most subgroups in both symbionts were enriched between 2- and 9-fold and with high significance in the set of induced genes relative to the whole genome (Fig. 8). At these early stages, both partners respond to the other’s presence by activating transcription, cell wall metabolism and protein turnover. The mycobiont’s specific responses center on upregulating membrane transporters, secreted hydrolases, and small proteins, broadly resembling the symbiotic responses of the EM fungus *L. bicolor* [112]. The photobiont’s specific responses center on growth and motility through the predominant activation of DNA and signal transduction processes, as well as protein trafficking and zoospore formation (flagella). This correlates with the alga’s rapid initial growth visible in coculture compared to monoculture (Additional file 6), possibly reflecting the growth observed in nature in free-living trebouxoid algae near suitable fungal hyphae [29] and increasing the chances of successful formation of new lichen initials. This issue is taken up again in Conclusions, where the importance for lichen evolution of re-lichenization of free-living algae released from natural thalli is discussed. It needs underlining that this coculture system and similar ones [113] do not proceed beyond formation of poorly differentiated lichenoids (Additional file 6), and thus they do not capture the complete interaction network extended in space and time needed for proper lichenization in nature. Individual genes of potential symbiotic significance are discussed in Inferences from differential transcription about nutritional fluxes at the symbiotic interface section and in Additional file 6.

#### Inferences from differential transcription about nutritional fluxes at the symbiotic interface

**Fig. 10:**
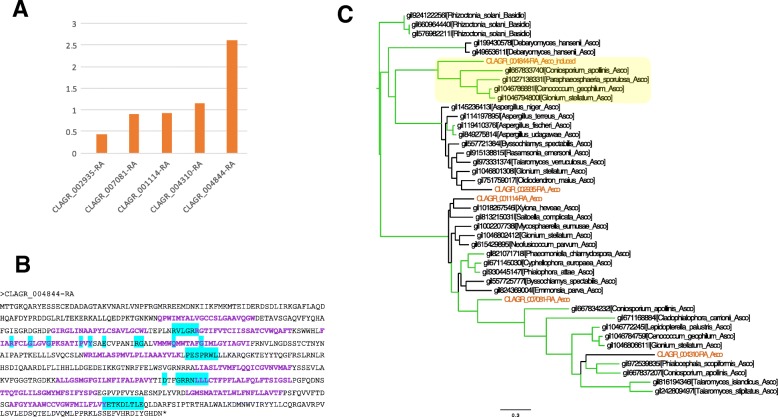
A predicted ribitol transporter in *C. grayi*. **a** Differential transcription in coculture vs. monoculture of five putative sugar transporters in *C. grayi*. They were the top five BLAST hits obtained by querying the genome with the sequences of two functionally validated fungal D-sorbitol/D-mannitol/ribitol transporters [117]. Only CLAGR_004844-RA is induced in coculture (Co/Mo on Y axis; 1 means no induction.). **b** CLAGR_004844-RA amino acid sequence. The 12 transmembrane domains [118] are indicated in bold purple. Consensus amino acids for sugar transporters [119, 120] are highlighted in cyan. **c** Protein phylogeny (PhyML, 100 bootstraps) of the five *C. grayi* transporters. The green branches correspond to nodes with bootstrap support ≥70%. The *C. grayi* proteins are labeled brown. The other transporters are identified by GI number and by fungal species. Taxa are also labeled as ascomycetes (Asco) or basidiomycetes (Basidio), the latter used as outgroup. The CLAGR_004844-RA clade is highlighted yellow. Bar indicates amino acid substitutions/site

Whereas hexose sugars are the carbohydrates transferred from plant to fungus in mycorrhyzae [114], polyols are the means by which trebouxoid algae transfer carbon to the fungus in lichens [115], and the first putative polyol transporter gene in a lichen fungus was identified recently [116]. We identified a family of five putative ribitol transporters in *C. grayi* (Additional file 6 and Fig. 10); in coculture, only one of the five was induced (Table 3 and Fig. 10), suggesting its involvement in importing ribitol to the fungus. The alga has dozens of putative sugar/MFS transporters whose expression remains mostly unchanged in coculture, except for seven that are weakly induced (Co/Mo 1.13–1.27). Their involvement, if any, in ribitol export to the fungus remains to be determined.

**Table 3:** Selected fungal (CLAGR) and algal (Aster) genes of potential symbiotic significance differentially expressed in coculture

Gene name	Hypothetical function	Co/Mo ratio	Gene name	Hypothetical function	Co/Mo ratio
A. Genes potentially involved in C and N exchange					
CLAGR_004844-RA	Ribitol transporter	2.6	Aster-02360	Ammonium transporter	1.0
CLAGR_005116-RA	ATO (Amm. exporter)	5.6	Aster-02123	Ammonium transporter	0.9
CLAGR_006601-RA	ATO (Amm. exporter)	0.9	Aster-04935	Ammonium transporter	1.0
CLAGR_003233-RA	ATO (Amm. exporter)	0.3	Aster-08051	Ammonium transporter	1.0
CLAGR_006538-RA	ATO (Amm. exporter)	0.4	Aster-08052	Ammonium transporter	n.d.
CLAGR_000407-RA	Mep2-type Amm. transp.	0.9			
CLAGR_009781-RA	Mep3-type Amm. transp.	0.6			
CLAGR_005848-RA	Mep1a-type Amm. transp.	0.6			
CLAGR_003366-RA	Mep1b-type Amm. transp.	1.5			
B. Other genes discussed in Additional file 6					
CLAGR_010764-RA	KP4 killer toxin-like	150	Aster-04252	Thioredoxin	73
CLAGR_008646-RA	Lectin	10	Aster-01625	Kinesin motor	2.3
CLAGR_010932-RA	Lectin	4	Aster-01936	Fasciclin domain protein	1.7
CLAGR_011186-RA	Gα signaling subunit	2	Aster-06761	Fasciclin domain protein	1.5
CLAGR_002910-RA	Regulator of Gα signaling	4	Aster-03695	Calcium channel	1.3
CLAGR_002710-RA	Dual specificity phosphatase	2			
CLAGR_007359-RA	DNA methyltransferase	3			
CLAGR_000113-RA	Calcium transporter	2			

It has been known since the 1970s that nitrogen is transferred from lichen mycobionts to eukaryotic photobionts [121, 122], and more recently it was suggested that the fungus acts as a nitrogen gateway for the alga [123]. This hypothesis is consistent with the results of a study of the lichen *Cladonia portentosa*, which found that the response of the algal proteome to nitrogen excess was more muted than that of the fungal proteome [43]. As is thought to be the case with mycorrhizal fungi [114], a primary form of nitrogen released by the lichen mycobiont to the alga is likely to be ammonium, although in lichens amino acids might be involved as well [124]. Ammonium transporters generally import NH_4_^+^ into the cell, but they can also export it [125–128]. Of particular interest are two ancient likely horizontal gene transfers of ammonium transporters from prokaryotes to fungi [129]. Transporters from the earliest transfer are found in all fungi, whereas transporters from the later transfer were derived from hyperthermoacidophilic Archaea and were retained primarily by lichenizing fungi [46, 129], suggesting a specific role in the symbiosis. Their selective retention might have been driven by the lichens’ frequent exposure to high temperatures and by the very low pH of their apoplast due to the high content of acidic secondary metabolites. Of the four MEthylammonium Permease-type (MEP) ammonium transporters in the *C. grayi* mycobiont, two belong to the general fungal class (CLAGR_000407-RA and CLAGR_009781-RA) and two to the class primarily retained by lichens (CLAGR_005848-RA and CLAGR_003366-RA). The *Cladonia* mycobiont also has four transporters belonging to the Gpr1/FUN34/YaaH family, whose functions are debated [130] but include the postulated ability to export NH_4_^+^ in yeast [128], where they are named ATO (Ammonia Transport Outward). The differential transcription of these eight transporter genes is interesting: relative to fungal monoculture, in coculture with the alga five are repressed, two are induced (one strongly), and one is unchanged (Table 3). The repression of a majority of these fungal transporters in coculture echoes the selective repression by the ectomycorrhizal fungus *A. muscaria* [131] of a fungal NH_4_^+^ importer at the symbiotic interface, thought to prevent re-absorption by the fungus of exported NH_4_^+^. Awaiting experimental verification, we speculate that the ATO transporter CLAGR_005116-RA (Co/Mo 5.6) and perhaps the Mep1b-like ammonium transporter CLAGR_003366-RA (Co/Mo 1.5) are candidates for mediating ammonium export to the alga, while the others, probably importers, are repressed to reduce re-absorption by the fungus. In the cocultured alga relative to monoculture, expression of four of its five ammonium transporters does not change and that of the fifth is undetectably low (Table 3). Overall, however, the transcriptional shifts in coculture of the fungal ammonium transporters are consistent with NH_4_^+^ being a major mediator of nitrogen transfer from mycobiont to photobiont. They are also consistent with the inability of freshly isolated *A. glomerata* to grow on nitrate (Specific survey of photobiont proteins involved in nitrate and CO2 assimilation section).

Phosphate is an important nutrient known to be transferred from mycorrhizal fungi to their plants [114], but there is no suggestive transport polarity or coculture induction pattern among the many putative phosphate transporters in *A. glomerata* and *C. grayi* that could shed light on this system. (For other individual genes induced in coculture, see Table 3, Additional file 6).

### Search for symbiosis-specific genes II: evolution of gene family size

Lineage-specific expansion or contraction of multigene families is often associated with lineage-specific functional shifts in eukaryotes [57, 58, 132–134]. We undertook one broad and one circumscribed analysis of multigene families. For the broad survey, multigene families in *C. grayi* and *A. glomerata* were identified using MCL [135] and analyzed for changes in family size using CAFE [136], with the taxa listed in Methods. In *C. grayi*, 390 families were expanded, 3369 showed no change, and 769 families had undergone contraction by comparison to a putative common ancestor. In *Asterochloris,* 648 families were expanded, 2729 showed no change, and no families were contracted. The circumscribed analysis was limited to the *C. grayi* transportome and predicted 458 *C. grayi* membrane transporters using the TransportTP online tool [137] with the Transporter Classification Database [138]. We discuss only families for which we can suggest symbiotic roles and highlight whenever a significant fraction of a gene family is also induced in coculture. We assume that the overlap of induction with expansion/contraction (Additional file 1) increases the likelihood that the genes involved play symbiotic roles. We did not find significantly contracted families in *A. glomerata*, although a separate search focused on nitrogen assimilation revealed a reduced set of nitrate assimilation genes in the alga (Specific survey of photobiont proteins involved in nitrate and CO2 assimilation section).

#### Mycobiont expanded families

**Fig. 11:**
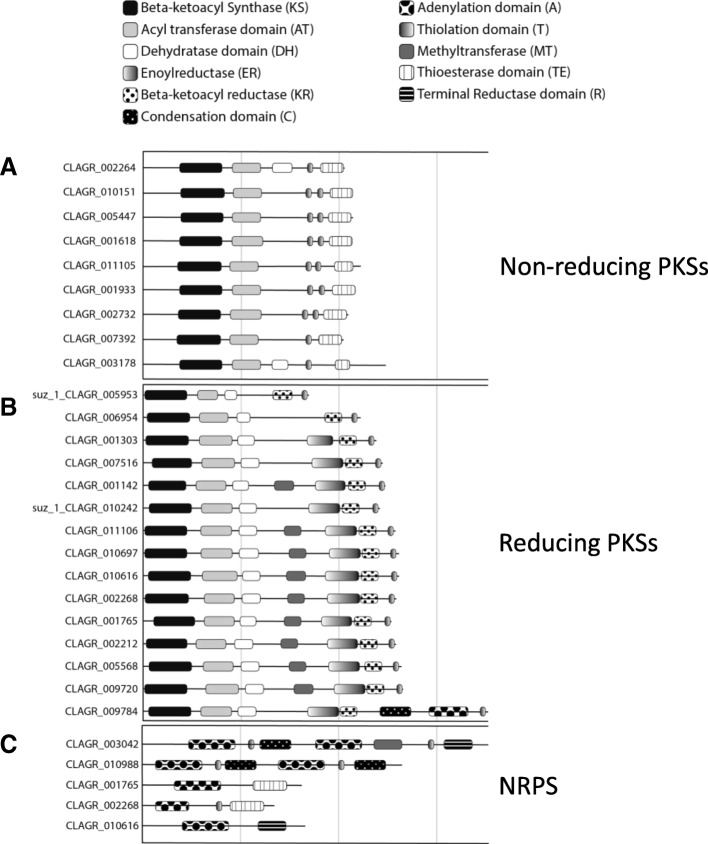
Polyketide Synthase (PKS) and Non-Ribosomal Peptide Synthetase (NRPS) genes in *C. grayi*. The three protein categories in **a**, **b**, **c** are named on the right. CLAGR_009784 is a PKS-NRPS hybrid. The only PKS whose likely downstream product (grayanic acid) is known is CLAGR_002732 [34]. The length of the horizontal lines is proportional to gene length (vertical gray lines delimit 3-Kb segments). Graphic symbols for protein domains are indicated at top. Genes with a suz_1 prefix were reannotated manually. See Additional file 7 for further details

In the *C. grayi* fungus, the most notable expansions involve 204 HET incompatibility proteins, 13 of which are also induced in coculture; 156 Ankyrin domain proteins; an 80-member family (Fam_5) of multi-transmembrane-domain proteins of unknown function with 10 coculture-induced members; Fam_668, a fructosamine kinase family with 7 members of which 3 are coculture induced; the family of polyketide synthases (PKSs) and Non-Ribosomal Peptide Synthases with at least 29 members. HET proteins might prevent incompatible *C. grayi* hyphae from fusing with a *C. grayi* mycobiont already lichenized with its photobiont. Ankyrin domains could be involved in inter-protein contacts at the symbiotic interface. Fructosamine kinases are known to reverse aging-associated protein damage produced by glycation [139, 140]. A few of the PKSs [34, 141] have been shown to be involved in the synthesis of the primary and most abundant secondary metabolites well known in lichens, but the large number of PKSs in *C. grayi* (Fig. 11 and Additional file 7) points to a vast and still uncharacterized metabolic potential. Further description of these expanded families and elaboration of the hypotheses on their possible symbiotic significance are found in Additional file 7. A separate analysis identified expanded families of signal transduction components (Specific survey of mycobiont and photobiont signal transduction components section and Additional file 8), some also differentially expressed in coculture (Additional files 6 and 8).

#### Mycobiont contracted families

Carbohydrate transporter Fam_1, MFS (Major Facilitator Superfamily) Fam_2 and Fam_7, and amino acid permease Fam_22 are dramatically reduced, and some members of these families are also induced in coculture (Additional file 7) or score as slow-evolvers as discussed in Slow-evolving proteins and anti-stress strategies in the mycobiont section. The contraction of these families is part of the overall contraction observed in the specific analysis of the mycobiont’s transportome (Additional file 7). A reduced transportome has been observed also in the ectomycorrhizal ascomycete *T. melanosporum* [58]. This is consistent with the loss of generalist nutrient uptake and the reliance of the fungus on a few specialized carbon sources from the photobiont, which are polyols in green algal lichens like *C. grayi* [115] (see Inferences from differential transcription about nutritional fluxes at the symbiotic interface section).

#### Photobiont expanded families

**Fig. 12:**
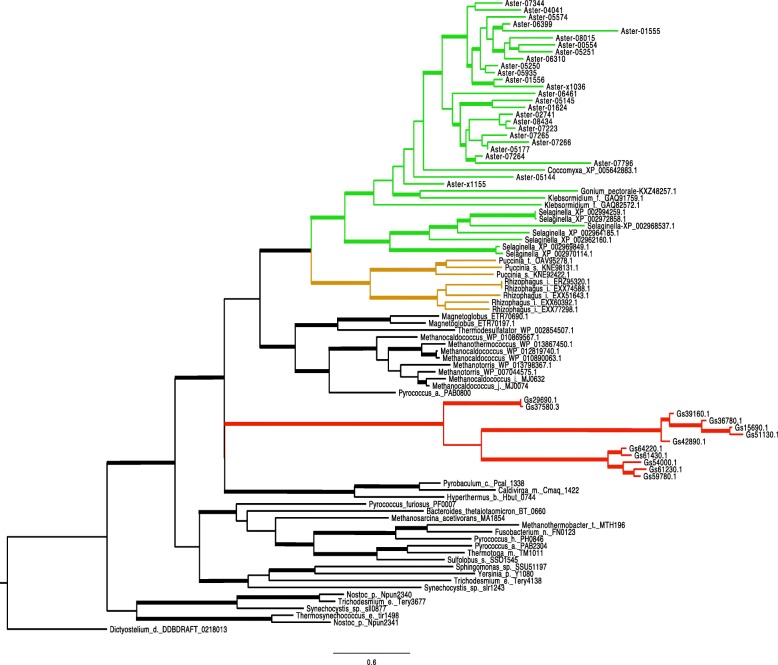
Protein family tree of archaeal ATPases. The phylogram was constucted using FastTree [144] on a MAFFT [145] amino acid alignment of 91 putative archaeal ATPases from prokaryotes and eukaryotes. Branches with bootstrap values ≥0.77 are thickened. Bar indicates amino acid substitutions/site. To the ATPases present in the published *Galdieria* phylogeny [146], we added all the proteins above *Methanocaldococcus*_j__MJ0632 in this figure. The eukaryotic taxa shown represent most of those currently known to harbor putative archaea-derived ATPases. ATPases from *Galdieria* (Gs) are marked red, from green algae and plants green, from fungi brown. All branches in black are prokaryotic, except for the amoeba *Dictyostelium* at the base. Branch labels include the taxon name or symbol and a protein identifier. The *Asterochloris* (Aster) proteins are indicated by their gene names in the JGI database [68]. The phylogeny suggests several independent HGT events, but it cannot exclude a very ancient HGT from Archaea to a common eukaryotic ancestor followed by losses in most eukaryotes. *Asterochloris*, *Galdieria*, and *Selaginella* have the largest families of archaeal ATPases (with 26, 12, and 7 members, respectively). See also Additional file 7

Additional file 7: The most notable expansions in *A. glomerata* involve the 100-member Fam_16 of unknown proteins unique to this alga, with 20% of the members induced in coculture; Kinase families totaling 52 members; Carbohydrate Active Enzymes (CAZ) with a total of 40 members; a 29-member Ankyrin domain protein family; a probably HGT-derived family of 26 archaeal ATPase-like proteins (Additional file 7 and Fig. 12). A survey using Phyre2, a protein structure prediction program [142, 143], suggests DNA-related functions for some Fam_16 members. The expansion of kinases matches the expanded signal transduction capability mentioned in Genome sizes and gene organization section. The expanded CAZ families might enhance the structural and biochemical versatility of the photobiont’s cell surface. The parallel expansion of Ank-domain proteins in *C. grayi* and *A. glomerata* might mediate reciprocal boundary interactions. Finally, the putative ATPases of archaeal origin and the Desiccation-Related-Proteins of bacterial origin might be involved in the lichen alga’s resistance to desiccation and elevated temperatures. Further description of these expanded families and elaboration of the hypotheses on their possible symbiotic significance can be found in Additional file 7.

#### Specific survey of mycobiont and photobiont signal transduction components

**Fig. 13:**
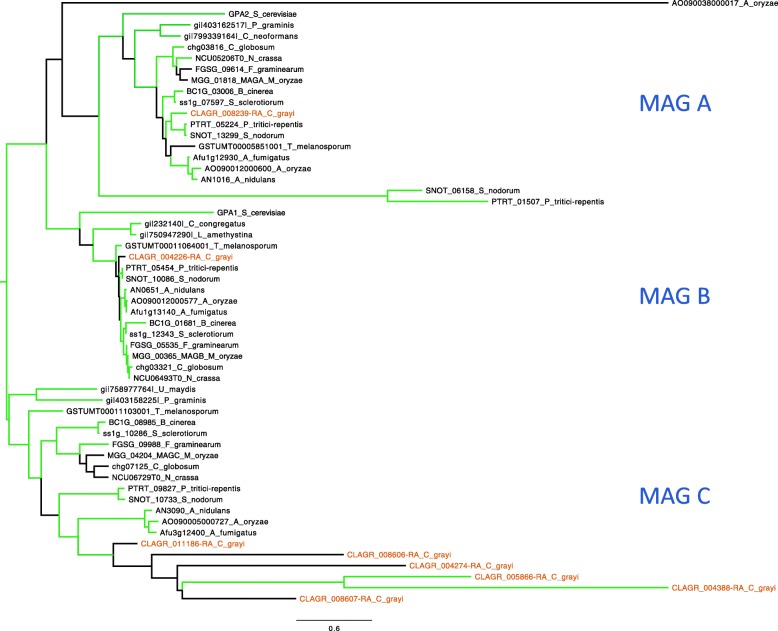
A unique set of Gα subunits is present in *Cladonia*. The protein phylogeny (PhyML, 100 bootstraps) of the eight *C. grayi* Gα subunits clusters into three major MAG A, MAG B, and MAG C clades (highlighted). The unique MAG C paralogs are shown on the bottom. The green branches correspond to nodes with bootstrap support ≥67%. The *C. grayi* proteins are labeled brown. Bar indicates amino acid substitutions/site. See also Additional file 8

This analysis confirms the suggestion from the data in Additional file 3 that signal transduction protein families are expanded in *C. grayi* and *A. glomerata* (Genome sizes and gene organization section). For *C. grayi*, these include PTH11-type receptors [147] and components of MAPK pathways described in Additional file 8, together with coculture expression data collected to identify signal transduction candidates potentially involved in early fungus-alga interactions (Additional files 6 and 8). The analysis also revealed an expanded set of five divergent Gα subunit paralogs in *C. grayi* (Fig. 13). The *Asterochloris* expansions (Additional file 8) involve a more diverse set of families than *Cladonia*’s.

#### Specific survey of mycobiont and photobiont transcription factor (TF) families

**Fig. 14:**
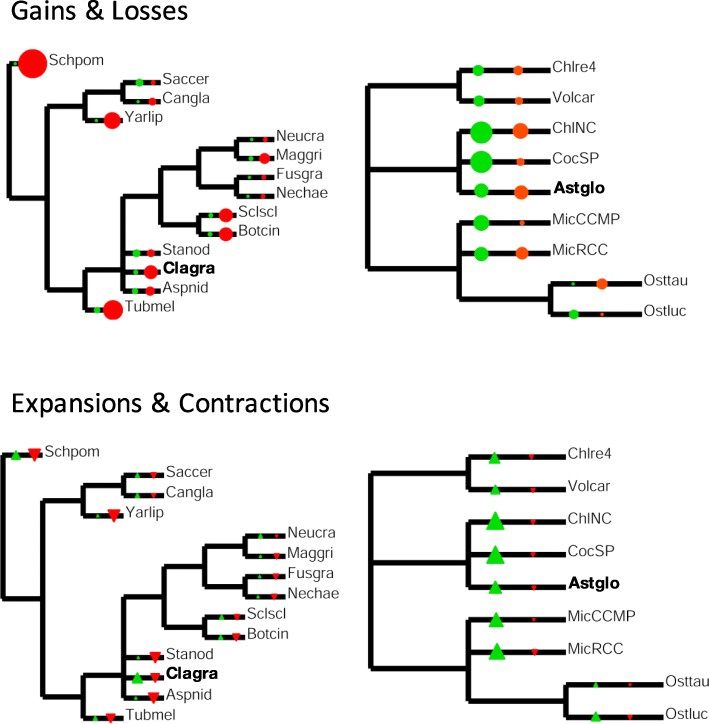
Evolution of transcription factor/regulator families in fungi (left) and algae (right). All the species used and numerical data are listed in Additional file 9. The *C. grayi* and *A. glomerata* abbreviations are bolded. Area of symbols is proportional to the change observed. Green circles: number of families gained, red circles: number of families lost. Green triangles: number of expanded families, red triangles: number of contracted families

Overall results are displayed in Fig. 14, and specific families are listed in Additional file 9. With regard to overall TF family gain or loss, neither *Cladonia* nor *Asterochloris* stand out within their respective phylogenies. With regard to family expansion and contraction, *Asterochloris* also conforms to the general algal phylogeny trend of expansions outnumbering contractions. In contrast, *Cladonia* appears to have experienced more expansions of TF families than not lichenized fungi. One *Cladonia* expansion involves a group of proteins characterized by PCI domains [148] (Additional file 9). PCI-domain proteins are involved in many diverse processes, and we could not find specific roles for these TFs. With regard to families with known functions, both *Cladonia* and *Asterochloris* have expanded TFs involved in chromatin remodeling (SWI/SNF [149] in *Cladonia*, SWI/SNF_Baf60 [150] and Sir2 [151] in *Asterochloris*) and stress responses (SGT [152] and CSD [153] in *Cladonia*, WRKY [154] in *Asterochloris*). *Asterochloris* has also contracted the SET family and the CCHC zinc finger family. The contracted SET family comprises protein lysine methyltransferases, involved in methylation of histones and other proteins [155]. We hypothesize that selection on both symbionts’ ability to shuttle reversibly between free-living and symbiotic states [31] and interact in the thallus in different ways across time and space [10] produced the needed genomic plasticity through the parallel expansion of chromatin-remodeling functions. In contrast, the significant SET family contraction seems odd, given the multifaceted effects of protein lysine methylation [156]. We suggest that the stresses placed on lichens by repeated, rapid and large oscillations in their exposure to light, temperature and hydration might have led to the expansion of stress-related TFs in both symbionts. These environmental stresses appear to affect the evolution of many lichen adaptations [44, 157–160] as discussed in Slow-evolving proteins and anti-stress strategies in the mycobiont section for the fungus (where several converge on maintaining the resilience of the proteome) and in Additional file 7 for the alga.

#### Specific survey of photobiont proteins involved in nitrate and CO_2_ assimilation

**Fig. 15:**
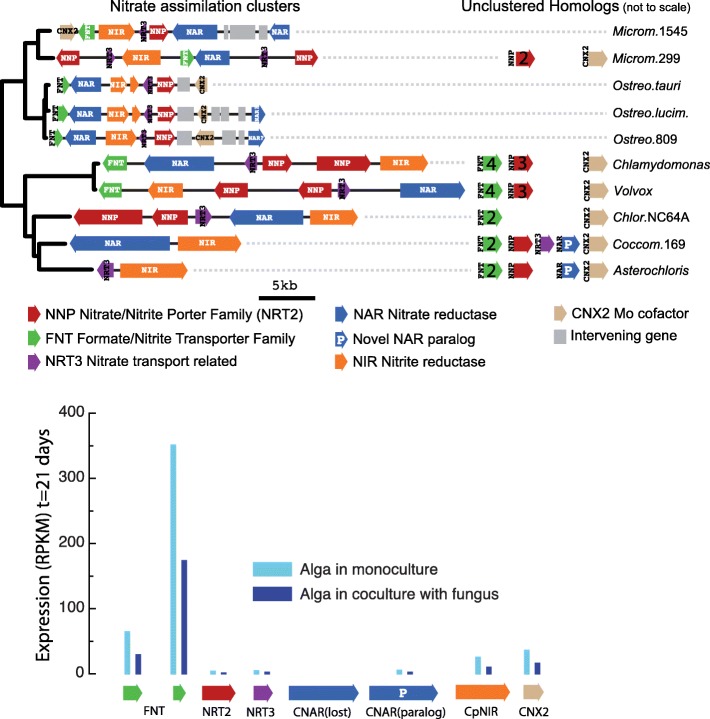
Nitrate assimilation gene clustering in Chlorophytes. In the upper part of the figure, the algal phylogeny (left) and the corresponding taxa (right) bracket the gene clusters and unclustered paralogs in each taxon. Gene and cluster lengths are to scale; color codes and acronyms are listed below the 5 kb bar. Phylogeny and clusters were obtained as described in Methods. The lower part of the figure displays as vertical bars the expression levels of the nitrate assimilation genes in the alga grown alone or with the fungus. The full names of the taxa listed from top to bottom are: *Micromonas pusilla* CCMP1545; *Micromonas* RCC299; *Ostreococcus tauri*; *Ostreococcus lucimarinus*; *Ostreococcus sp.* RCC809; *Chlamydomonas reinhardtii*; *Volvox carteri*; *Chlorella variabilis* NC64A; *Coccomyxa subellipsoidea* C-169; *Asterochloris glomerata.* All the corresponding genome data are at [164]

We compared *A. glomerata* to related algae for possible adaptations involving nitrogen and carbon assimilation proteins that did not surface in the protein family screens. The top of Fig. 15 displays the types and organization of nitrate assimilation genes linked to a phylogenetic tree for Chlorophytes. *A. glomerata* has a reduced set of nitrate assimilation genes compared to other green algal genomes in this figure. The nitrate assimilation cluster (HANT-AC) [161] present in most microalgae [162] is reduced to nitrite reductase (NIR) and the NRT3 transporter in *A. glomerata*, and the total number of NNP transporters is reduced in *Coccomyxa subellipsoidea* C-169 and *A. glomerata*. *A. glomerata* has lost the clustered nitrate reductase (NAR), but it retains a nitrate reductase paralog (NAR-P) also found in *C. subellipsoidea*. Nitrate cannot be taken up directly by the lichenized alga enclosed in fungal tissue but has to be first metabolized into other compounds by the mycobiont [123]. These compounds may include amino acids, excreted by the fungus and presumably taken up by the alga [124], and ammonium (see Inferences from differential transcription about nutritional fluxes at the symbiotic interface section). Congruent with these findings, *A. glomerata* isolated from *C. grayi* can grow on nitrate only after a period of adaptation to axenic culture (Armaleo, unpublished). Day 21 gene expression in mono- and coculture is graphed on the bottom of Fig. 15. Interestingly, nitrate assimilation genes in *A. glomerata* are turned down about 50% in coculture relative to monoculture, which may contribute to the regulation of growth experienced by the alga when it lichenizes with the fungus [163]. We hypothesize that, while *A. glomerata* is capable of autonomous nitrate assimilation utilizing nitrate transporters and NAR-P when free-living, suppression of these mechanisms during symbiosis has relaxed selection on their strict maintenance, resulting in the loss of a full HANT-AC and a reduction in the number of nitrate transporters. The extreme reduction of the nitrate assimilation toolkit in *A. glomerata* relative to other non-symbiotic chlorophytes could be viewed as a parallel to the transportome contraction in *C. grayi* (Mycobiont contracted families section and Additional file 7), each contraction resulting from the almost exclusive nutritional reliance of each partner on the other.

As algal photosynthesis is central to the lichen symbiosis, we surveyed the carbonic anhydrases (CAs) of *Asterochloris*, enzymes that catalyze the interconversion between CO_2_ and HCO_3_^−^. This adds to previous extensive work published on CAs in lichen algae [165, 166]. Delivery of CO_2_ to the enclosed lichen photobionts is extremely variable, depending on the specific anatomy and physiology of its mycobiont-photobiont combination [167] and on rapid changes in temperature and in the supply of nutrients, water, and light [168]. When lichen thalli become fully saturated with water, the diffusion of CO_2_ to the algae is further limited by the swollen hyphal tissue surrounding them. This reduces photosynthetic rates [169, 170] even if the hydrophobicity of the lichen interior maintains air-filled spaces for rapid CO_2_ diffusion [10]. Our data, detailed in Additional file 10, suggest that *Asterochloris* parallels *Chlamydomonas* and *Chlorella* in having one α CA functioning in the pyrenoid but differs in having expanded a specific subclass of cytoplasmic β CAs, whose potential relationship to the symbiosis needs exploring.

### Search for symbiosis-specific genes III: proteins with anomalous rates of evolution

We hypothesize that, after a burst of adaptive selection at the origin of Lecanoromycetes 300–350 million years ago [171–174], proteins that acquired fundamental symbiotic roles would have since stabilized under purifying selection and evolved at slower rates, as suggested for a polyketide synthase specific for a secondary metabolite unique to *C. grayi* [34]. Conversely, faster evolving proteins are likely to represent more recent changes. We compared amino acid substitution rates of *C. grayi* and *A. glomerata* proteins to those of eleven free-living ascomycetes and six unicellular chlorophytes respectively using three methods (Methods). We focus on the proteins whose rates were “slow” or “fast” relative to nonlichen species by at least two methods to reduce false positives. This yielded 38 slow- and 11 fast-evolving candidates in the fungus and 3 slow- and 7 fast-evolving candidates in the alga (Slow-evolving proteins and anti-stress strategies in the mycobiont and Fast-evolving proteins sections).

#### Slow-evolving proteins and anti-stress strategies in the mycobiont

Additional file 11 (Excel sheet “Fungus slow-evolvers”) lists a total of 72 proteins that score in multiple ways (highlighted in orange) for potential symbiotic significance. The first 38 were identified as slow-evolving by at least two of the rate methods. Of the 38, 22 are universal eukaryotic components of the protein translation, translocation and sorting machinery (Additional file 11). This machinery maintains proteostasis, the dynamic equilibrium of the proteome, through a complex network guiding protein folding and functionality from synthesis to modification, to sorting, and to degradation [175–177]. Studies with several eukaryotic systems, including yeast, show that downturns in protein translation and translocation are essential to protect cells from dehydration, heat, and hyperosmotic stresses [178–184]. In yeast for instance, many temperature-sensitive mutations impairing a variety of ribosome assembly steps also dramatically increase desiccation tolerance [185], highlighting how slowdowns in ribosomal assembly may reduce protein misfolding and aggregation during desiccation. However, lichens must withstand daily cycles of dehydration, rehydration, thermal and UV radiation stress so extreme that they would kill most other organisms [157, 186]. Therefore, we hypothesize that such exceptional circumstances in early lichen evolution selected for “upgraded” fungal components of the ribosomal biogenesis, mRNA processing, and protein trafficking networks, which were already part of the normal Environmental Stress Response (ESR) [184]. The upgrades were then stabilized under purifying selection and integrated with other defenses [157] involving, for instance, antioxidants [159, 187] and synthesis of photoprotective anthraquinones [188] that lichens share with non-extremophiles. There is some correlative support in the literature for the centrality of ribosomal function in the lichen response to stress. In the lichen *Lobaria pulmonaria* [37] as much as 35% of the expressed fungal proteome is involved in ribosomal and protein turnover functions. In the lichen *Cladonia rangiferina* [39] global transcriptional responses to desiccation and rehydration also suggest involvement of the protein translation machinery. In the cultured lichen fungus *Endocarpon pusillum,* ribosomal protein genes are highly induced during PEG-induced dehydration stress [159]. We speculate that the unusually high number of nuclear rDNA introns (Additional file 12) present in lichen fungi [189, 190] could be another possible adaptation to control the rate of ribosomal assembly for stress (e.g. desiccation) protection. A major consequence may be the well-known slow growth phenotype of lichens and lichen mycobionts. Other double-scoring slow evolving proteins of interest are listed and discussed in Additional file 11. These include a putative osmosensing calcium channel and two homogeneous groups of proteins: 18 membrane transporters and 7 aldehyde dehydrogenases (ALDHs).

#### Fast-evolving proteins

Additional file 11 includes a description of 11 putative fast-evolving mycobiont proteins involved in signal transduction, membrane trafficking and stress protection, as well as slow- and fast- evolving proteins in the photobiont.

## Conclusions

### Symbiotically relevant genes affect stress resistance, nutritional, signaling, and structural interactions

Our analysis identified several proteins expected to influence the symbionts’ environmental stress resistance. They include slow evolving mycobiont proteins (Slow-evolving proteins and anti-stress strategies in the mycobiont section, Additional file 11) enriched for universal eukaryotic components of the protein translation, translocation and sorting machinery which manages the proteome under normal as well as stress conditions like dehydration and heat [178–182]. Also, some of the faster evolving *C. grayi* proteins, probably under adaptive selection, appear to be involved in protection from stress (Additional file 11). In *A. glomerata*, the likely horizontally transferred archaeal ATPases and Desiccation-Related Proteins may contribute to its heat and desiccation resistance (Photobiont expanded families section, Additional file 7). Transcription factor family expansion includes proteins involved in chromatin remodeling and stress responses in both symbionts (Specific survey of mycobiont and photobiont transcription factor (TF) families section).

We also identified proteins governing several symbiotically relevant nutrient interactions. The fungus in coculture induced two transporters potentially central in the carbon and nitrogen exchange at the symbiotic interface: one an importer for ribitol, the carbon source provided by trebouxoid algae to their fungal partners [115] (Inferences from differential transcription about nutritional fluxes at the symbiotic interface section and Additional file 6), the other a possible ammonium exporter (Inferences from differential transcription about nutritional fluxes at the symbiotic interface section), pointing at NH_4_^+^ as a major nitrogen source provided by the mycobiont to the photobiont. Reliance of each partner on the other as a restricted nutrient source is also reflected by the contraction of the sugar transportome in *C. grayi* (Mycobiont contracted families section) and by the reduced nitrate assimilation potential in *A. glomerata* (Specific survey of photobiont proteins involved in nitrate and CO2 assimilation section). Unknown is the significance for symbiotic carbon fixation of the expansion of a specific *A. glomerata* subclass of cytoplasmic carbonic anhydrases, enzymes that catalyze the interconversion between CO_2_ and HCO_3_^−^ (Specific survey of photobiont proteins involved in nitrate and CO2 assimilation section and Additional file 10).

Gene families whose characteristics suggest involvement in other symbiotically relevant interactions comprise: signal transduction proteins, expanded in both symbionts, which include a unique new set of five MAG C paralogs in the mycobiont (Specific survey of mycobiont and photobiont signal transduction components section and Additional file 8); ankyrin domain protein families (Mycobiont expanded families and Photobiont expanded families sections), also expanded in both partners, perhaps involved in increased protein-protein interactions at the boundaries between them; algal glycosyl transferase families, whose expansion could be necessary to adapt extracellular surfaces to the varied contacts in which the photobiont engages (Photobiont expanded families sections, and Additional file 7); the expanded set of mycobiont polyketide synthases producing compounds for a mostly undiscovered array of biochemical functions (Mycobiont expanded families section); HET incompatibility protein families in *C. grayi*, some possibly involved in the competition among fungal genotypes to secure the appropriate alga (Mycobiont expanded families section). Also the induction in coculture of secreted proteins in the fungus and less prominently in the alga (Extended analysis of transcription induced in coculture section) suggests their involvement in a variety of unexplored symbiotic functions.

### The evolution of lichenization involved changes in many conserved genes scattered throughout the symbionts’ genomes

Most of the symbiotically relevant genes suggested here have homologs in non-lichen fungi and algae, and we assume that they are variants modified by symbiosis. This indicates that lichens evolved mainly through the accumulation of scattered regulatory and structural changes in available genes rather than through sudden key innovations. This in turn suggests that, to establish a basic and reversible nutritional dependency at very early evolutionary stages, the free-living ancestors of myco- and photobionts might have required at first only a few or even no changes. A model of such basic interactions was developed experimentally using the fungus *S. cerevisiae* and the alga *C. reinhardtii* [191]. Selection towards increasing stability and environmental adaptability would have then transformed such precarious mutualistic/antagonistic and reversible states into lichens over evolutionary time. This scenario suggests that there were multiple pathways for fungi, algae and cyanobacteria to evolve into lichens, which is consistent with the emerging consensus that ascolichens could have had a few independent evolutionary origins [171, 192]. It is also compatible with the fact that lichens display a wide array of structures with different levels of complexity, from leprose and crustose to fruticose and foliose, and with the overall staggering variety of interactions throughout the biosphere between fungi and photosynthetic organisms [193, 194].

### Sexual reproduction, symbiont autonomy and equivalence

While the advantages accrued by lichens through sexual reproduction of their mycobionts are fairly clear [91, 92, 97, 195–197] and consistent with the behavior of a mutualistic exhabitant [100], the advantages of sex for the algal inhabitant were previously expected to be limited [100], mostly based on the assumption that the lichen alga is asexual and that it dies when its lichen thallus dies [198]. As indicated in Heterothallism probably evolved from homothallism in Cladonia; genetic evidence for sex in Asterochloris section, however, there is strong evidence that sex in trebouxoid algae does occur and that free photobiont populations exist on the substrates near lichen thalli [27, 29, 199–201], so that sexually produced variation and algal adaptations to lichenization are likely to be incorporated and selected for in lichenized populations. W.B. Sanders proposed in 2005 [29] that the transient free-living state is necessary for completion of the algal sexual cycle, which is consistent with the fact that the rare direct observations of algal sexual stages have always involved aposymbiotic cells (Heterothallism probably evolved from homothallism in Cladonia; genetic evidence for sex in Asterochloris section). Encounters of germinating mycobiont spores and free-living photobionts [199] could produce lichens with new combinations of both genomes, expanding and fine-tuning a lichen’s adaptation to different ecological niches. Photobiont contributions to lichen adaptability are in fact highlighted by several studies. For a given lichen, the correlation of algal genotypic variation with habitat appears stronger than that of the fungal genotype [103, 202–205]. Other analyses indicate that both fungal and algal genotypes substantially influence a lichen’s ecological adaptability [206, 207], and that multiple algal genotypes can coexist within single thalli [208–210] and move horizontally among fungal genotypes [207, 208, 211–214] even when the predominant means of photobiont transmission is vertical through vegetative propagules [201, 208]. We propose therefore that, over hundreds of millions of years of tight coexistence, genomic and functional autonomy in each partner were maintained by the benefits of periodically detaching each partner from the symbiosis for sex and for partner switching, which increased the overall adaptability of the lichenized symbionts. Beyond sex, the list of phycobiont investments in the symbiosis is long. It includes the adaptations suggested in Photobiont expanded families, Specific survey of mycobiont and photobiont signal transduction components, Specific survey of mycobiont and photobiont transcription factor (TF) families, Specific survey of photobiont proteins involved in nitrate and CO2 assimilation sections, the intrinsic resilience of the free-living lichen alga to desiccation [44, 160], the increased resistance to PSII photoinhibition in the symbiotic vs. free-living alga [215], and correlated structural and physiological adaptations: algal morphology changes significantly between lichenized and free-living states ([216] and references therein) and, when the lichen is hydrated, about 50% of the fixed carbon can be converted to lichen biomass [217] with an energy conversion efficiency comparable to that with which chloroplast photosynthesis translates into plant biomass [218]. Ribosomal DNA introns occur in lichenized trebouxoid algae [219–221], including the Group IB intron in the LSU gene of *A. glomerata* [221]. We speculate, as for the mycobiont rDNA introns (Slow-evolving proteins and anti-stress strategies in the mycobiont section), that these photobiont rDNA introns may be involved in mediating desiccation tolerance. The evolutionary introduction of *A. glomerata* or Trebouxiales to lichenization is not yet known but, based on the only published broad Chlorophyta chronogram [222] (which does not include Trebouxiales), the timing could be compatible with that estimated for lichen fungi [173, 174]. It is therefore possible that the *Asterochloris/Trebouxia* lineage has been adapted to symbiosis for hundreds of millions of years. Metaphorically, the lichen alga is not the “second sex” [223]: it deserves a full seat at the symbiotic table.

## Methods

Where appropriate, methodological information is included in figure and table legends.

### Biological isolates

The sequenced mycobiont is the *C. grayi* single-spore isolate *Cgr/DA2myc/ss* [34] (Culture Bank accession # CBS 132746; GenBank ITS accession # KC592272) [224]. The sequenced photobiont is the *Asterochloris* strain *Cgr/DA1pho*, isolated from *C. grayi* soredia as described [64]. The species was identified by ITS1 sequencing and 100% identity to *Asterochloris glomerata* [48]. Natural *C. grayi* podetia used for EST generation were harvested, and chemotype was confirmed by Thin Layer Chromatography as described [34].

### Genomic DNA extraction

The *C. grayi* mycobiont was grown for ~ 9 weeks in 50-ml cultures of HMY (MY according to Hamada [225]: 10 g/L malt extract; 4 g/L yeast extract; 4 g/L sucrose) in 125-ml flasks shaking at room temperature, with periodic medium changes and grinding to improve growth. Typically, after 3 weeks of growth, a 50-ml mycelial culture was first ground and then expanded as follows. A Polytron homogenizer (Kinematica GmbH, model PT 10/35) fitted with an autoclaved standard 10-mm diameter probe (Brinkmann generator PTA 10S) was set up in a laminar flow hood with the probe at a 45^o^ angle. The 50-ml culture was then sterilely ground in the tilted flask for 5 s at half-maximal setting. Using wide bore pipets, four 10-ml aliquots from the ground culture were transferred to four 125-ml flasks, each with 40 ml of fresh HMY. After every grinding, absence of contamination in each flask was tested by spreading 1 ml of the ground and diluted culture onto an HMY test plate and periodically monitoring growth by microscopy. Mycelia were harvested from the liquid cultures by filtration through nylon mesh, rinsed on the filter with sterile TE, lyophilized, weighed, and stored at -80^o^ C. DNA was extracted from 850 mg mycelia (dry weight). The sample was thoroughly ground in a mortar with liquid nitrogen; the powder was transferred to a 50-ml polypropylene tube and resuspended in 18 ml (~ 20/1, *v*/*w*) of lysis buffer (40 mM TrisHCl pH 8; 20 mM Na Acetate; 1 mM EDTA; 1% *w*/*v* SDS) plus freshly added RNAse to 0.1 mg/ml. The suspension was repeatedly mixed with a pipet while being kept at 65^o^ C for 5 min. A 5 M NaCl solution (6 ml) was mixed in, and the suspension was centrifuged at 3 K rpm for 5 min on a tabletop centrifuge to pellet cell debris, polysaccharides and some proteins. The supernatant was extracted with an equal volume of a 1:1 phenol:chloroform mix saturated with 100-mM Tris HCl at pH 8. The aqueous phase was brought to 65^o^ C, and 0.11 volumes of CTAB solution (10% CTAB; 0.7 M NaCl) were mixed in. The mixture was extracted with an equal volume of chloroform. An equal volume of room-temperature isopropanol was mixed into the aqueous phase, and the solution was aliquoted into six 12-ml polypropylene tubes and centrifuged at 5^o^ C for 15 min at 6 K rpm. After completely removing the supernatants, the procedure was repeated from the lysis buffer step in a concentrated and simplified fashion. Each pellet was first left undisturbed at 65^o^ C for 3–5 min in 150 μl of lysis buffer plus RNAse to facilitate resuspension. The six pellets were then thoroughly resuspended by pipetting, suspensions were pooled into one of the six tubes, and 1/3 volume (300 μl) of 5 M NaCl was mixed into the pool. The solution was aliquoted into two 1.5-ml microfuge tubes, undissolved residue was removed by benchtop centrifugation for 5 min at 15 K rpm, and the supernatants were extracted once with chloroform. Two volumes of ice-cold 100% ethanol were added to the aqueous phases, and the precipitated DNA was pelleted by benchtop centrifugation at room temperature. The pellets were rinsed with 70% ethanol, air-dried in a laminar flow hood for 30 min and left undisturbed to rehydrate on ice in TE buffer overnight. They were then resuspended by gentle pipetting, and the solutions were pooled. The total yield was 127 μg of high molecular weight DNA, verified by Qubit quantitation and gel electrophoresis.

*Asterochloris glomerata* was incubated on AMY plates (MY according to Ahmadjian [226]: 20 g/L malt extract; 2 g/L yeast extract) for 4 weeks at room temperature under constant light (photon flux = 40 μmol m^− 2^ s^− 1^). Algae were harvested from one plate, transferred to sterile deionized water in a microfuge tube, pelleted, lyophilized, weighed and stored at -80^o^ C. DNA was extracted from 83 mg algae (dry weight) as described [64], with one modification: after the final resuspension of the DNA in TE, the volume of TE was increased to 1 ml, the sample was transferred to a 12-ml Falcon tube, and the DNA was re-precipitated by adding 330 μl of 5 M NaCl followed by 2.7 ml of cold 100% EtOH. The solution was mixed by inversion and centrifuged at 6 K rpm for 10 min at 5^o^ C. The pellet was rinsed with 70% EtOH, air-dried in a laminar flow hood for 30 min and left undisturbed to rehydrate on ice in TE buffer overnight. It was then resuspended by gentle pipetting. The total yield was 110 μg of high molecular weight DNA, verified by Qubit quantitation and gel electrophoresis.

### Genomic DNA sequencing, assembly, annotation and data storage

For the mycobiont, 50,883,772 Illumina single-end reads (36 b, 52 x coverage) were pre-assembled with Velvet [227]. Contigs larger than 500 b were subsequently fragmented into 159,239 sub-sequences, 300 b long and with 100 b overlaps. These sub-sequences were assembled with Newbler (Roche) together with 1,486,692 single-end 454 reads (14 x coverage) and a random sub-library of 45,000 paired-end 454 reads. The resulting mycobiont 34.6 Mb draft assembly contained 414 scaffolds >2Kb, with an N50 of 243,412. For the photobiont, 3,020,503 single-end 454 reads and 2,159,218 paired-end 454 reads (24.8 x total coverage) were assembled with Newbler (Roche), resulting in a 56 Mb draft assembly with 151 scaffolds and an N50 of 784,872. Completeness of draft assemblies was assessed by screening for the presence of 248 core eukaryotic genes with CEGMA (Core Eukaryotic Genes Mapping Approach) [228], a now discontinued pipeline to annotate highly conserved Core Eukaryotic Genes (CEGs). In the mycobiont assembly, 234 (94.35%) CEGs were fully recovered, and scores increased to 241 (97.18%) if partial hits were considered as well. In the photobiont assembly, 216 complete (87.10%) and 235 partial (94.6%) CEGs were recovered. Gene model annotation was performed with MAKER2 [229]. For the mycobiont, assembled *C. grayi* mRNA-seq reads (see next section) were used as EST evidence, and the gene prediction tool SNAP [230] was trained through MAKER2 with *C. grayi* protein annotations from the earlier JGI genome release [231] and the 248 eukaryotic core protein sequences of CEGMA. Annotations were additionally complemented by Genemark-ES [232] trained on the assembled *C. grayi* genome, and generated models were passed to MAKER2. For the photobiont, assembled *A. glomerata* mRNA-seq reads (see next section) and *Coccomyxa sp.* C-169 ESTs [233] were used as EST evidence, and SNAP was trained through MAKER2 with *A. glomerata* protein annotations from the earlier JGI genome release [234]. Annotations were additionally complemented by Genemark-ES trained on the assembled *A. glomerata* genome, and generated models were passed to MAKER2. In the mycobiont, 11,398 gene models were predicted, including 115 isoforms, and 10,075 gene models were predicted in the photobiont. The annotated and searchable genome assemblies are at [67] for the mycobiont and at [68] for the photobiont.

### RNA extraction and EST generation from whole lichen thalli

Total RNA for EST generation was extracted from freshly harvested *C. grayi* podetia treated with RNAlater (ThermoFisher) to prevent RNA degradation. Dirt was removed with tweezers, and podetia were left to dry at room temperature and moisture for 2 h and weighed (200 mg). RNAlater permeates dry better than moist tissue, and RNA quality is not significantly affected by this brief period of “natural” drying, as lichens are exposed to drying in nature. RNAlater was added (2 ml/100 mg podetia) and the suspension was vortexed until all air pockets were removed, indicating an even permeation of the solvent into the tissues. Podetia were kept in RNAlater at room temperature for about 1 h with occasional mixing. Excess RNAlater was removed by centrifugation, followed by gentle blotting of the podetia with filter paper. Podetia were lyophilized overnight and stored at -80^o^ C. Podetia were thoroughly ground in liquid nitrogen with a mortar, and RNA was extracted with the RNAqueous Total RNA Isolation Kit (Life Technologies) according to the manufacturer’s protocol with two modifications. For the first, the initial buffer volume in which the ground powder was resuspended was increased to 5 ml Lysis Buffer mixed with 680 μl of Plant Isolation Aid for 200 mg of air-dried podetia. For the second, a DNAse-treatment step was inserted as follows. After loading the lysate onto the cartridge and centrifuging to remove residual Lysis Buffer-Plant Isolation Aid solution, 500 μl of Wash 2/3 solution were centrifuged through the cartridge. The cartridge was then centrifuged again without added liquid to remove all traces of Wash 2/3. After moving the cartridge to a new collection tube, the matrix was wetted by adding 60 μl of DNAse solution (52 μl RNase-free water, 6 μl 10xDNase buffer Promega M198A, 2 μl RQ1 RNase-free DNase Promega M610A). Under these conditions, nucleic acids detach from but remain within the matrix. After incubating the cartridge with DNase at 37^o^ C for 15 min, 300 μl of a 1:1 mix of Lysis Buffer: 64% ethanol were added to allow the RNA to bind again to the matrix. The cartridge was kept at room temperature for 5 min, and the liquid was removed by centrifugation. The manufacturer’s protocol was resumed by adding 700 μl of Wash 1. The final yield was 27 μg of purified RNA. Agilent microchip electrophoresis showed a RIN = 9. The RNA was used to prepare a 454 cDNA library of 740,680 reads averaging around 500 bases in length. Of the reads, 76.2% mapped onto the mycobiont genome and 12.1% onto the photobiont genome. The residual unmapped reads are from other eukaryotic or prokaryotic inhabitants of natural *C. grayi* thalli. Reads were assembled into ESTs using Newbler (Roche), Velvet [227] and Trinity [235], and mapped to the assembled symbiont genomes to aid in the annotation (see preceding section).

### RNA extraction and sequencing for analysis of differential expression in coculture

To analyze differential gene expression during symbiont coculture in vitro, RNA was isolated from mycobiont and photobiont grown on filters in agar plates. The culturing details are as described [33]. Briefly, mycobiont and photobiont were routinely propagated as described for the DNA extractions. To set up filter cultures, separate fungal and algal cultures were harvested and filtered first through gauze and then through 74 μm nylon to obtain uniform suspensions either of small mycelial fragments or of algal cells. Cell density of the separated suspensions was measured by OD, and fixed amounts (dry weight equivalents: 2.9 mg mycobiont and/or 1.3 mg photobiont) were uniformly delivered by suction onto 0.22 μm pore-size nitrocellulose filters (Millipore, GSWP04700) to obtain cultures of either fungus alone, alga alone, or the two together. The seeded filters were placed on 1.5% agarose (Sigma-Aldrich A9539) plates (one filter per plate) with “99:1” medium [33] and incubated for 21 days at room temperature under a constant photon flux of 40 μmol m^− 2^ s^− 1^ (Fig. 6). The main methodological difference relative to Joneson et al. (2011) [33] was the addition of RNAlater at harvest. Each filter was peeled off the agarose surface and placed over three stacked Whatman 9-cm filter paper disks in the lid of a 10-cm plastic Petri dish. To exchange residual growth medium for RNAlater, 2 × 1 ml of RNAlater were evenly pipetted over the entire surface of the nitrocellulose filter culture. After the RNAlater was absorbed by the Whatman filters, the nitrocellulose filter was placed (culture facing up) into the empty bottom half of the Petri dish, and 1 ml RNAlater was uniformly pipetted over it. While submerged in the RNAlater layer, the culture was scraped off the nitrocellulose with a sterile blade, pooled to the edge in RNAlater by tilting the dish, and the suspension was pipetted into a microfuge tube. Addition of 1 ml RNAlater, scraping and pooling were repeated once more, and the two suspensions were kept in two tubes for 1 h at room temperature. To obtain a single pellet from the two tubes corresponding to one filter, the two suspensions were centrifuged sequentially in the same tube, the RNAlater was discarded, and the pellet was lyophilized and stored at -80^o^ C. Each frozen pellet was ground with a fitting pestle in its microcentrifuge tube cooled in liquid nitrogen [31]. The ground powder derived from one filter culture was resuspended in 500 μl of Lysis Buffer-Plant Isolation Aid combination (RNAqueous Total RNA Isolation Kit, Life Technologies), and RNA was extracted according to the manufacturer’s instructions except for the insertion of a DNAse treatment step as described in the preceding section. For each growth condition, the extracts from two replicate cultures were combined. RNA yields per combined cultures were 3 μg, 7 μg and 16 μg respectively from fungus alone, alga alone, and coculture. Agilent microchip electrophoresis showed RINs near 9. The RNA was used to produce cDNA libraries totaling 156 million RNAseq reads. Reads were aligned using Bowtie [236]. Gene expression was calculated using BowStrap [237] with the annotated gene models from the mycobiont and photobiont genomes at JGI. Gene expression is defined as re-sampled Reads Per Kilobase of gene sequence per Million aligned sequences (RPKM). For the GO term-centered analysis, differential expression was represented as the ratio between the base 2 logs of each gene’s coculture and monoculture RPKMs on day 21. Genes with log fold changes > 0.6 (for upregulation) and < 0.6 (for downregulation) were analyzed. Statistical significance (*p* < 0.05) was based on hypergeometric distribution enrichment of genes or pathways relative to their representation in the whole genome. For the more extensive analysis (Extended analysis of transcription induced in coculture section), the ratio between each gene’s coculture and monoculture RPKMs on day 21 (referred to here as Co/Mo) was taken directly to represent differential expression, and only ratios > 2 for the mycobiont or > 1.3 for the photobiont were considered induced. To combine the induced genes/proteins (795 in the mycobiont and 471 in the photobiont) into functionally related protein subgroups, we collected information from GO terms, PFAM domains, and literature searches (Additional file 6). Statistical significance (p < 0.05) for the coculture induction of each resulting subgroup (Fig. 8) was based on that subgroup’s hypergeometric distribution enrichment within all induced proteins relative to the representation, in the whole genome, of genes encoding proteins similar to those in that subgroup. Such proteins were identified (e-value <1e^− 5^) by using the subgroup as query in a BLASTP genome search.

### Predictions of multigene families, PKSs, and secreted proteins

Protein sequences for multigene family analysis were retrieved from the Joint Genome Institute Portal [164] and the Broad Institute Portal [238]. As references for *Cladonia grayi*, the following fungal taxa were used: *Stagonospora nodorum* (*Phaeosphaeria nodorum*) (16,597 models), *Botrytis cinerea* (*Botryotinia fuckeliana*) (16,389 models), *Sclerotinia sclerotiorum* (14,522 models), *Neurospora crassa* (9907 models), *Aspergillus nidulans* (10,753 models), *Aspergillus fumigatus* (9887 models), *Aspergillus oryzae* (12,017 models), *Tuber melanosporum* (7496 models), *Fusarium graminearum* (13,826 models), *Pyrenophora tritici-repentis* (12,169 models) and *Chaetomium globosum* (11,103 models). As references for *Asterochloris glomerata*, the following algal taxa were used: *Coccomyxa* sp. C169 (9629 models), *Chlorella* sp. NC64A (9791 models), *Chlamydomonas reinhardtii* 169 (17,113 models), *Ostreococcus tauri* (7725 models), *Volvox carteri* (14,542 models), and *Micromonas sp*. CCMP1545 (10,545 models). Orthogroups of mutual best BLAST hits present in the symbiont genomes and all reference species (2455 in *C. grayi*, 1454 in *A. glomerata*) were further analyzed. Protein groups were aligned with MAFFT [145] using *Tuber melanosporum* or *Volvox carteri* as outgroups, and alignments were subsequently trimmed with GBLOCKS [239, 240] (*A. glomerata*) and Guidance [241] (*C. grayi*). Proteins were queried in an all-against-all BLASTP analysis, and protein families were subsequently predicted with MCL [135] with inflation parameters set to 3.0. In the fungal data set, 5051 protein families were identified that were phylogenetically relevant (containing at least two species and 10 sequences), of which 4528 were found in *C. grayi*. In the algal data set, 3545 phylogenetically relevant gene families were identified. *C. grayi* PKSs and NRPSs were predicted using antiSMASH v3.0 [242], followed by manual curation. Secreted proteins in *C. grayi* and *A. glomerata* were predicted as indicated in the legend of Fig. 9.

### Analysis of gene family expansions and contractions

For the broad analysis encompassing all *C. grayi* and *A. glomerata* genes, multigene families predicted as described in the previous section were analyzed for evolutionary changes in protein family size using CAFE [136]. The program uses a random birth and death model of gene gain and loss across a user-specified tree structure. The distribution of family sizes generated under the random model provides a basis for assessing the significance of the observed family size differences among taxa (*p*-value < 0.001). CAFE estimates for each branch in the tree whether a protein family has not changed, has expanded or has contracted. For the analysis of *C. grayi* family expansion and contraction centered on the transportome, predicted proteins containing at least three transmembrane domains were extracted from the same fungal genomes used in the broad analysis and listed in the previous section. These predicted proteins were classified according to the Transport Classification database [138] using the TransportTP on-line tool [137, 243]. For several families, classifications were checked manually with predicted fungal transporter proteins retrieved from TCDB, TransportDB [244], and the *Saccharomyces* Genome Database [245] used as queries in BLASTP and TBLASTX searches against the 12 fungal genomes. Lineage-specific gene gains and losses were estimated using CAFE [136].

For the analysis centered on signal transduction components, predicted proteins were identified based on PFAM domains among the taxa indicated above. To identify family expansions and contractions, protocols similar to those indicated above were used. For the analysis centered on putative DNA-binding transcription factors (TFs), the proteomes of the fungal and algal taxa indicated in Additional file 9 were searched using the hmmscan module of HMMER version 3.0 [246] against a comprehensive DNA-binding domain database assembled from three compilations [247–249] to which corresponding PFAM domain designations were assigned. GA (gathering bitscore) thresholds for each domain were retrieved from PFAM 25.0, and each hit on a protein exceeding the appropriate GA threshold was recorded. Matches where alignments covered less than 80% of the corresponding PFAM profile HMM were discarded. Putative transcription factors were classified into families based on domain structure as described by Lang et al. [249] and rules were extended by incorporating fungal-specific terms. A random birth and death model [250] was used to investigate expansions, contractions, gains, losses and ancestral states of TF families across algal and fungal phylogenies [4, 251] (Fig. 14). The best model was selected from a series of models with increasing complexity by using likelihood ratio tests to drop parameters that did not further improve the likelihood of a model. After rate parameter optimization, posterior probability of ancestral gene content of each node and the evolutionary change for each family along the branches of the tree were estimated.

### Analysis of nitrate assimilation genes in chlorophytes

Homologs of nitrate assimilation-related (HANT) genes (*C. reinhardtii*: nitrate reductase, AAF17595.1; nitrite reductase, XP_001696787.1; NRT2, XP_001696788.1; formate/nitrite transporter, XP_001696698.1; *M. pusilla*: nitrite reductase, XP_003057941.1; molybdopterin synthase, XP_003057940.1) were determined by BLASTP of algal genome best protein models (*Asterochloris glomerata* Cgr/DA1pho v1.0, *Chlamydomonas reinhardtii* v4.0, *Chlorella sp.* NC64A v1.0, *Coccomyxa* sp. C-169 v2.0, *Micromonas pusilla* CCMP1545 v 2.0, *Micromonas commoda* NOUM17 (RCC 299) v 2.0, *Volvox carteri nagariensis* v 1.0, *Ostreococcus* sp. RCC809 v 2.0, *Ostreococcus lucimarinus* v 2.0, *Ostreococcus tauri* RCC4221 v3.0). Retained hits had at least 45% similarity with and covered between 50 and 150% of the length of the query. Homologs of the query genes were considered part of a nitrate assimilation cluster (HANT-AC) if separated by no more than 6 intervening gene models from another gene in the query [252]. Intervening and flanking sequence up to 10 kb of HANT-ACs were examined for unannotated nitrate assimilation-related genes by BLASTX against the NCBI non-redundant protein database; NRT3 was determined by the presence of NAR2 PFAM domain. Orthology and paralogy of nitrate assimilation genes were determined by examination of phylogenetic trees generated as follows: protein sequences were aligned with MAFFT v.7.2.2.1 [145] using default parameters, and poorly aligned regions were removed with trimAl v.1.4 using the “automated1” algorithm [253]. The best model of protein evolution (JTT, WAG, LG) was inferred by AICc using ProtTest v.3.4 [254], and maximum likelihood analysis was performed with RAxML v.8.1.20 [255]. Nitrite reductase phylogeny was estimated using the combined sequences from both BLAST searches and was reduced to models containing a PLN02431 protein domain. Expected but missing gene homologs were considered absent by TBLASTN of the respective genome assemblies. Algal species phylogeny was estimated by analysis of *RPB2* using the methods above.

### *A. glomerata* meiosis gene identification and phylogenetic analysis; flagellar proteins

We sought *A. glomerata* orthologs of multiple meiosis-specific genes proposed as a “meiosis detection toolkit” for identifying genomic evidence for sexual reproduction ([256, 257] and references therein) and select paralogs: *Msh4*; *Msh5*; *Asy1*/*Hop1*; *Ahp2*/*Hop2*; *Mnd1*; *Dmc1* (paralog *Rad51*—DNA repair in meiosis and mitosis); *Syn1*/*Rec8* (paralogs *Syn2*, *Syn3*, *Syn4*—cohesins not specific to meiosis [258]); *Spo11–1*, *Spo11–2* [259] (paralog *Spo11–3*—no reported meiotic phenotype; affects overall plant size, hormone responses, and endoreduplication [260]). We also sought orthologs of two additional meiosis-specific genes: *Pch2* [261] and *Mer3* [262, 263]. *Arabidopsis thaliana* orthologs of these 16 genes were used as TBLASTN [105] queries against the version 2 assembly of the *A. glomerata* genome and additional publicly available green algal genomes (*Ostreococcus* sp. RCC809, *Ostreococcus lucimarinus* CCE9901, *Micromonas pusilla* CCMP1545, *Chlamydomonas reinhardtii* CC-503 cw92 mt+, *Volvox carteri* f. *nagariensis* Eve (HK10 derivative), *Chlorella variabilis* NC64A, *Coccomyxa subellipsoidea* C-169; JGI, 2013). If initial *A. thaliana* queries failed, subsequently identified green algal orthologs were used for additional queries. All genome hits were compared to the NCBI non-redundant protein database by BLASTX and, for putative orthologs of the 16 genes listed above, manually annotated with a predicted protein sequence based on *A. thaliana* and revisions following amino acid multiple sequence alignment comparisons (MUSCLE v3.6 [264], the MEGA5.05 interface of MUSCLE [265]). Phylogenetic analyses (not shown) were done to test for orthology (e.g., distinguishing meiotic *Spo11–1* or *Spo11–2* from non-meiotic *Spo11–3*) and green algal origin (i.e., confirming that the putative *A. glomerata* orthologs fall into a clade with plants and green algae rather than fungal orthologs) using maximum likelihood (green algae and plants) or NJ (green algae, plants, select additional fungi) methods. This analysis identified 12 *A. glomerata* genes specific for meiosis and/or mitosis and one, *Spo11–3*, for endoreduplication (Additional file 5).

The 314 candidate flagella proteins in *A. glomerata* (Additional file 5) were identified by searching for putative orthologs among sequenced motile and non-motile chlorophytes using the reciprocal best BLASTP hit criterion (e-value<1e^− 5^). The queried Chlorophytes included *C. reinhardtii*; *Volvox carteri*; *Coccomyxa subellipsoidea* C-169; *Chlorella variabilis* NC64A; *O. tauri*; *O. lucimarinus*; *Micromonas pusilla* CCMP1545; *Micromonas* sp. RC299. The queried databases included the *Chlamydomonas* CiliaCut protein set [49], the *Chlamydomonas* flagella proteome [266, 267] and protein families ubiquitous in motile chlorophytes but absent in non-motile chlorophytes (Fig. 5). The latter proteins were referred to as Chlorophyte CiliaCut in reference to work by Merchant et al. [49], and contain candidate flagella proteins. A majority (69/95 = 68%) of the 95 *Asterochloris* proteins in the Chlorophyte CiliaCut are orthologs to proteins of the *Chlamydomonas* CiliaCut or *Chlamydomonas* flagella proteome.

### Tests of relative evolutionary rates

Three methods were used to assess evolutionary rates in *C. grayi* and *A. glomerata* relative to the 11 fungal and 6 algal taxa and alignments described in the multigene family prediction methods. The first protocol used HyPhy [268] to determine substitution rates for each individual conserved orthogroup from aligned mutual best BLAST hit orthologs present in all taxa (2455 in the ascomycetes and 1454 in the chlorophytes). Rates of *C. grayi* and *A. glomerata* genes were considered reduced or accelerated if predictions matched in at least 90% of all analyzed triplets and rates were below the significance threshold (*p*-value <= 0.05), while only nonsignificant values were scored in remaining triplets. In the second protocol, HyPhy was applied to the multigene families predicted through MCL [135]. To detect anomalous evolutionary rates, the third protocol used likelihood ratios applied to the same orthogroups used in the other protocols. Since it is applied here for the first time, the third protocol is detailed in Additional file 13.

## Additional files

Additional file 1: Organelle genomes and maps. (DOCX 5930 kb)

Additional file 2: Synteny between *Asterochloris* vs. *Coccomyxa* or *Chlorella*. (DOCX 583 kb)

Additional file 3: KEGG-based functional gene categories in the *C. grayi* symbionts. (DOCX 758 kb)

Additional file 4: A 500-Kb DNA virus insertion left a clear footprint in the *A. glomerata* genome. (DOCX 100 kb)

Additional file 5: Sexual reproduction and candidate genes. (ZIP 182 kb)

Additional file 6: In vitro culture and gene expression. (ZIP 1530 kb)

Additional file 7: Expanded and contracted families. (ZIP 220 kb)

Additional file 8: Signal transduction diversification. (ZIP 3420 kb)

Additional file 9: Transcription factor family expansions and contractions. (XLSX 30 kb)

Additional file 10: Carbonic anhydrases in *A. glomerata*. (DOCX 217 kb)

Additional file 11: Slow- and fast-evolving proteins. (ZIP 104 kb)

Additional file 12: Locations and lengths of nuclear rDNA introns in *Cladonia grayi*. (DOCX 130 kb)

Additional file 13: Likelihood-based heterotachy detection method. (DOCX 22 kb)

## Abbreviations

AICc: Corrected Akaike’s information criterion
ALDH: Aldehyde dehydrogenase
AM: Arbuscular mycorrhizae
ANK: Ankyrin domain
ATO: Ammonia Transport Outward
BLAST(P): Basic Local Alignment Search Tool (Proteins)
BLASTX: Protein databases search using a translated nucleotide query
CA: Carbonic anhydrase
CAFE: Computational Analysis of gene Family Evolution
CAZ: Carbohydrate Active Enzymes
CCHC: Cys_2_ His Cys zinc finger domain
CEG: Core eukaryotic gene
CEGMA: Core Eukaryotic Genes Mapping Approach
Co/Mo: Coculture to Monoculture expression ratio
CTAB: Cetyltrimethyl ammonium bromide
DRP: Desiccation-Related Protein
dsDNA: Double-stranded DNA
EM: Ectomycorrhizae
ESR: Environmental Stress Response
EST: Expressed Sequence Tags
GO: Gene Ontology
GPCR: G Protein-Coupled Receptor
HANT-AC: High Affinity Nitrate Transporter-Assimilation Cluster
HET: Heterokaryon incompatibility
HGT: Horizontal gene transfer
ITS: Internal Transcribed Spacer
JTT: Jones-Taylor-Thornton model of protein evolution
KEGG: Kyoto Encyclopedia of Genes and Genomes
LAT: L-type amino acid Transporter
LG: Le-Gascuel model of protein evolution
LSU: Large ribosomal subunit gene
MAG: Magnaporthe grisea-like Gα protein
MAPK: Mitogen Activated Protein Kinase
Mb: Megabases
MCL: Markov Cluster Algorithm
MEP: Methylammonium Permease-type transporters
MFS: Major Facilitator Superfamily
ML: Maximum likelihood
MY: Malt Yeast extract
NAR: Nitrate reductase
NAR-P: Nitrate reductase paralog
NCLDV: Nucleo-cytoplasmic large DNA viruses
NIR: Nitrite reductase
NJ: Neighbor Joining
NNP: Nitrate/Nitrite Porter
NRPS: Non-Ribosomal Peptide Synthases
NRT3: Nitrate transport related
ORF: Open Reading Frame
PFAM: Protein Family database
PhyML: Maximum Likelihood Phylogeny estimation
PKS: Polyketide synthase
PS II: Photosystem II
RGS: Regulators of G-protein Signaling
RIN: RNA Integrity Number
RPKM: Reads Per Kilobase of gene sequence per Million aligned sequences
SET domain: Su(var)3-9, Enhancer-of-zeste and Trithorax
SSU: Small ribosomal subunit gene
TBLASTN: Translated nucleotide databases search using a protein query
TBLASTX: Translated nucleotide databases search using a translated nucleotide query
TCDB: Transporter Classification Database
TE: Tris EDTA buffer
TF: Transcription factor
TM: Transmembrane
TransportTP: Membrane transporter prediction and characterization program
WAG: Whelan-Goldman model of protein evolution
