# Supplementary material for: The lichen symbiosis re-viewed through the genomes of Cladonia grayi and its algal partner Asterochloris glomerata
Source: BMC Genomics. 2019 Jul 23;20:605. doi: 10.1186/s12864-019-5629-x (PMC6652019; doi:10.1186/s12864-019-5629-x)
Supplement: Supplementary file 1 — Organelle genomes and maps. (DOCX 5930 kb) [file 12864_2019_5629_MOESM1_ESM.docx]

**Additional file 1**

**Organelle genomes and maps**

**Possible significance of a split tRNA-Ile lysidine synthase gene (*tilS*) in *A. glomerata***

Among nonlichen fungal mitochondrial genomes, genome size and gene order variation are common, and size variation is mostly due to variation in the numbers of group I introns rather than to the kind of genes present [1]. Lichen mycobiont mitochondrial genomes vary as well, but are generally smaller than in nonlichen fungi due to gene loss [2]. Size reduction is not observed in the photobiont: based on GenBank data, the *Asterochloris* organelle genomes are among the largest in green algae. Table 1 summarizes the basic features of the organelle genomes (see also maps below). The *C. grayi* mitochondrial genome (50,836 bp) is near the average (~60,500bp) of the lichen fungal mitochondrial genomes sequenced and annotated so far [2-4]. The *C. grayi* mitochondrial genome has 15 predicted ORFs for known protein coding genes (including *atp9* which is missing in some other lichen mycobiont mitochondria [2]) and one with no significant match in GenBank. It encodes one 23S RNA with two group I introns, one 16S rRNA, and a full set of 26 tRNA genes. The mitochondrial genome of *A. glomerata* (110,932 bp) contains a canonical arrangement of a single rRNA gene along with a 5S rRNA gene, encodes an almost full set of tRNAs, and has remnants of Group II introns. The chloroplast genome (217,546 bp) lacks *cysA*, a generally conserved putative sulfate transporter.

The *A. glomerata* chloroplast genome shares a split lysidine synthase gene with a few other Trebouxiophyceae taxa, all belonging to genera which include some lichenized members: *Coccomyxa* C-169, *Leptosira* (as *Pleurastrum*) [5], and the parasitic *Helicosporidium* [6]. The co-occurrence of split lysidine synthase genes in genera with symbiotic members prompted the following considerations. Lysidine synthases are ubiquitous in bacteria and chloroplasts and convert the wobble-position cytosine in the 3'-UAC-5' anticodon of a latent tRNA^Met^ into lysidine [7], producing the anticodon 3'-UAL-5'. This is necessary and sufficient to turn the latent tRNA^Met^ into a tRNA^Ile^, charged with isoleucine by Ile tRNA synthase and recognizing Ile AUA codons [8]. Why do bacteria and chloroplasts use 3'-UAL-5' to pair with the Ile codon AUA instead of using a 3'-UAU-5'? Experiments in bacteria show that, in the structural context of most bacterial tRNAs and ribosomes, 3'-UAU-5' anticodons would be prone to wobble-pair also with methionine AUG codons, whereas 3'-UAL-5' is strictly specific for isoleucine AUA codons [9, 10]. Although based on a limited number of known cases, the fact that the *tilS* gene is split only in the chloroplasts of four trebouxioid genera with mutualistic or parasitic species suggests a possible connection to symbiosis. The split separates the N terminal from the C terminal domains of the *tilS* protein, respectively involved in anticodon recognition/modification and in recognition of the acceptor arm of the tRNA [8]. We speculate that the separate transcription/translation into two components of what is commonly a single protein might add regulatory layers useful in a mutualistic or parasitic context.

**Organelle genome maps**

A

B

C

# A: Mitochondrial genome of *C. grayi.* Color legend, top right. "Other" (gray) refers to introns & homing endonucleases. B: Mitochondrial genome of *A. glomerata.* Color legend, top right. "Other" (gray) refers to protein-coding genes. C: Chloroplast genome map of *A. glomerata.* Color legend, top right. "Other" (gray) refers to protein-coding genes. All maps were generated with the online tool described in [11].

# References

1. Aguileta G, de Vienne DM, Ross ON, Hood ME, Giraud T, Petit E, Gabaldon T: **High Variability of Mitochondrial Gene Order among Fungi**. *Genome Biol Evol* 2014, **6**(2):451-465.

2. Pogoda CS, Keepers KG, Lendemer JC, Kane NC, Tripp EA: **Reductions in complexity of mitochondrial genomes in lichen-forming fungi shed light on genome architecture of obligate symbioses**. *Mol Ecol* 2018, **27**(5):1155-1169.

3. Xavier BB, Miao VPW, Jonsson ZO, Andresson OS: **Mitochondrial genomes from the lichenized fungi *Peltigera membranacea* and *Peltigera malacea*: Features and phylogeny**. *Fungal Biol-Uk* 2012, **116**(7):802-814.

4. Simon A, Liu, Y., Sérusiaux, E., Goffinet, B: **Extensive mitogenome rearrangement within the Peligerinae (lichenized Ascomycetes): comparison between *Ricasolia amplissima* and *Peltigera membranacea***. In*.* Abstract Book, 8th IAL Symposium, Poster: University of Helsinki; 2016: 115.

5. Friedl T: **Inferring Taxonomic Positions and Testing Genus Level Assignments in Coccoid Green Lichen Algae - a Phylogenetic Analysis of 18s Ribosomal-Rna Sequences from *Dictyochloropsis reticulata* and from Members of the Genus *Myrmecia* (Chlorophyta, Trebouxiophyceae Cl-Nov)**. *J Phycol* 1995, **31**(4):632-639.

6. Xavier BB: **Organelle Genomes of Lichens**. *Masters Thesis.* Reykjavik: University of Iceland; 2011.

7. Muramatsu T, Nishikawa K, Nemoto F, Kuchino Y, Nishimura S, Miyazawa T, Yokoyama S: **Codon and Amino-Acid Specificities of a Transfer-Rna Are Both Converted by a Single Post-Transcriptional Modification**. *Nature* 1988, **336**(6195):179-181.

8. Suzuki T, Miyauchi K: **Discovery and characterization of tRNA(Ile) lysidine synthetase (TilS)**. *Febs Lett* 2010, **584**(2):272-277.

9. Kohrer C, Mandal D, Gaston KW, Grosjean H, Limbach PA, RajBhandary UL: **Life without tRNA(Ile)-lysidine synthetase: translation of the isoleucine codon AUA in *Bacillus subtilis* lacking the canonical tRNA(2)(Ile)**. *Nucleic acids research* 2014, **42**(3):1904-1915.

10. Taniguchi T, Miyauchi K, Nakane D, Miyata M, Muto A, Nishimura S, Suzuki T: **Decoding system for the AUA codon by tRNA(Ile) with the UAU anticodon in *Mycoplasma mobile***. *Nucleic acids research* 2013, **41**(4):2621-2631.

11. Grant JR, Stothard P: **The CGView Server: a comparative genomics tool for circular genomes**. *Nucleic acids research* 2008, **36**:W181-W184.
