# Supplementary material for: The lichen symbiosis re-viewed through the genomes of Cladonia grayi and its algal partner Asterochloris glomerata
Source: BMC Genomics. 2019 Jul 23;20:605. doi: 10.1186/s12864-019-5629-x (PMC6652019; doi:10.1186/s12864-019-5629-x)
Supplement: Supplementary file 2 — Synteny between Asterochloris vs. Coccomyxa or Chlorella. (DOCX 583 kb) [file 12864_2019_5629_MOESM2_ESM.docx]

**Additional file 2**

**Synteny between *Asterochloris* *vs*. *Coccomyxa* or *Chlorella***

A total of 5,657 and 5,208 couples of putative orthologous proteins were identified between *Asterochloris* and *Coccomyxa subellipsoidea* and between *Asterochloris* and *Chlorella variabilis*, respectively, using the reciprocal best BLASTP hit criterion (e-value <1e^-5^). The relative location of orthologous genes on the genomic scaffolds was compared by means of a dot-plot matrix (Panels A and B below). The mostly random background distribution of dots indicates that widespread chromosomal rearrangements disrupted gene order over long distances, against which clusters of dots represent genes that remain syntenic in the same genomic neighborhoods. Most of the colocalized genes are not diagonally arrayed within a scaffold, however, indicating that frequent rearrangements occur also over short distances.

**Synteny dot plots of *Asterochloris vs. Coccomyxa* (A) or *Chlorella* (B) proteins**

**A**

*Coccomyxa* scaffolds


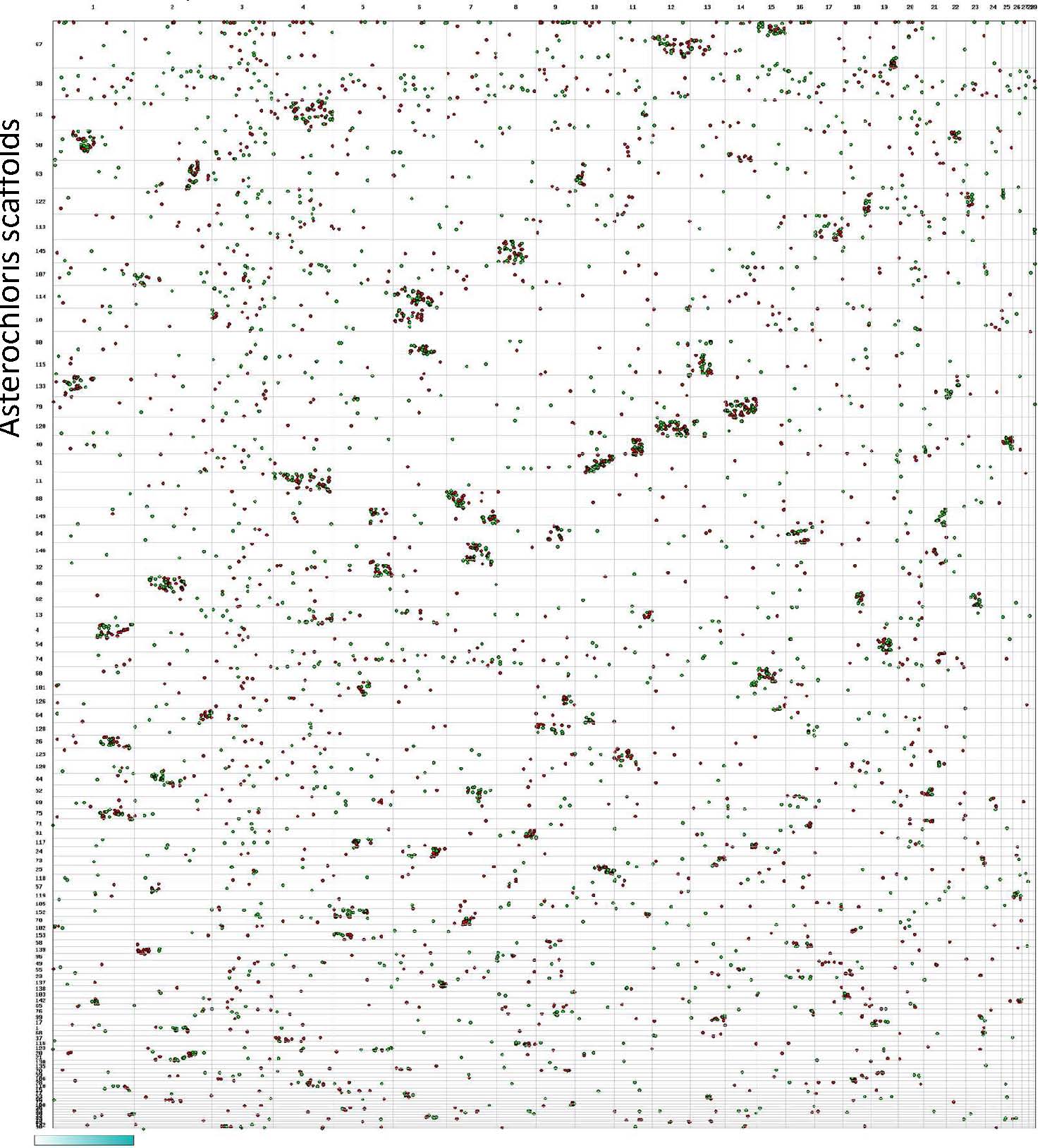


*Asterochloris* scaffolds

**B**

*Chlorella* scaffolds


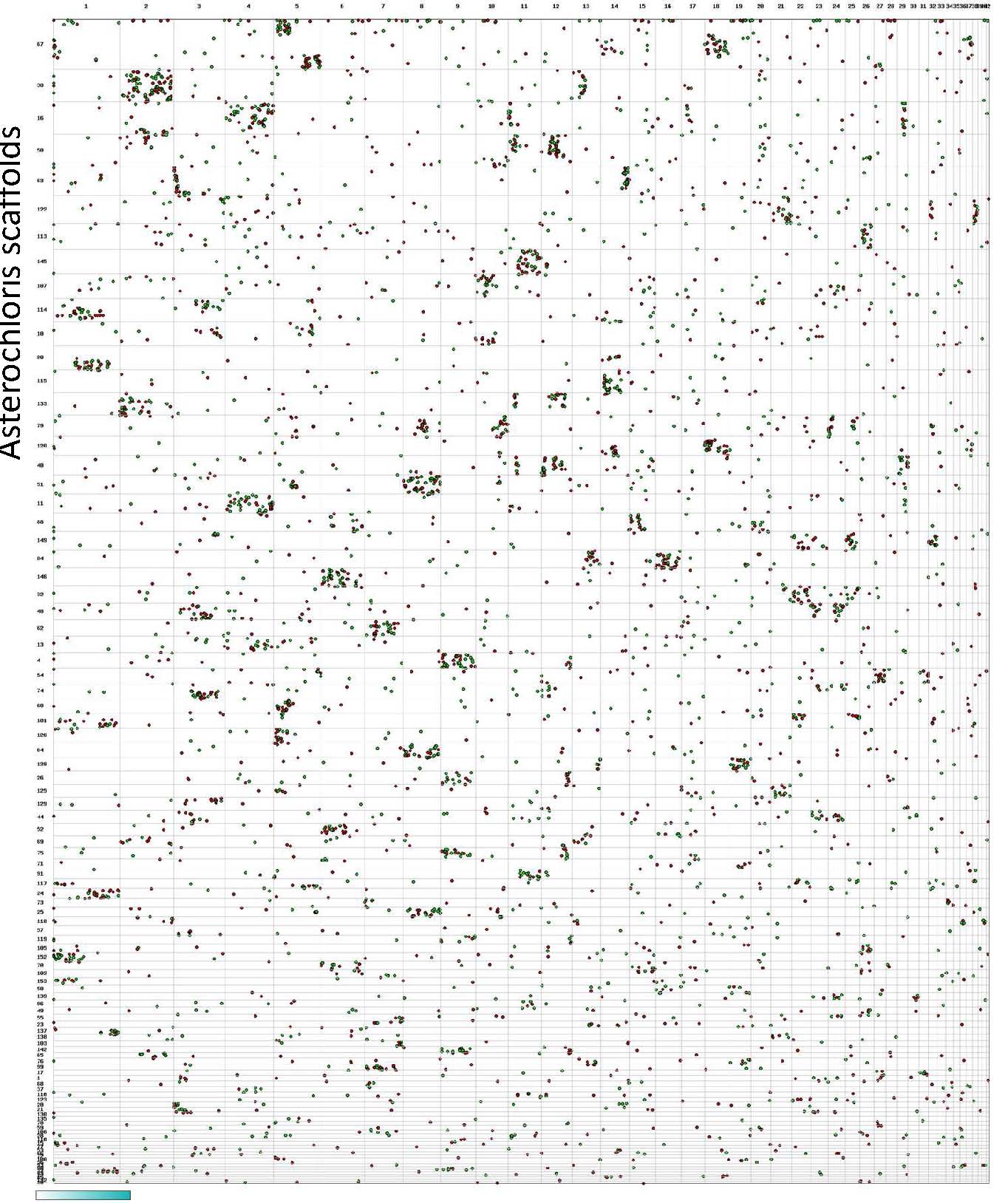


*Asterochloris* scaffolds

# The dots represent 5,657 (A) and 5,208 (B) reciprocal best BLASTP hits. Same and opposite strand hits are colored red or green, respectively. Scaffolds are listed from left to right and top to bottom in decreasing size order.
