# Supplementary material for: The lichen symbiosis re-viewed through the genomes of Cladonia grayi and its algal partner Asterochloris glomerata
Source: BMC Genomics. 2019 Jul 23;20:605. doi: 10.1186/s12864-019-5629-x (PMC6652019; doi:10.1186/s12864-019-5629-x)
Supplement: Supplementary file 3 — KEGG-based functional gene categories in the C. grayi symbionts. (DOCX 758 kb) [file 12864_2019_5629_MOESM3_ESM.docx]

**KEGG-based functional gene categories in the *C. grayi* symbionts**

Between 30% (*C. grayi*) and 40% (*A. glomerata*) of the total proteins were identified as part of a KEGG pathway. Additional annotations (Gene Ontology, MapMan) from *Chlamydomonas reinhardtii* and *Arabidopsis thaliana* were also used to increase coverage. The top two charts represent the four top-level functional categories. Each category is subdivided in the other pie charts. Notice the large representation of signal transduction functions in both symbionts.
