# Supplementary material for: The lichen symbiosis re-viewed through the genomes of Cladonia grayi and its algal partner Asterochloris glomerata
Source: BMC Genomics. 2019 Jul 23;20:605. doi: 10.1186/s12864-019-5629-x (PMC6652019; doi:10.1186/s12864-019-5629-x)
Supplement: Supplementary file 4 — A 500-Kb DNA virus insertion left a clear footprint in the A. glomerata genome. (DOCX 100 kb) [file 12864_2019_5629_MOESM4_ESM.docx]

**Additional file 4**

**A 500-Kb DNA virus insertion left a clear footprint in the *A. glomerata* genome**

The low-GC region contains 462 genes, 57 genes on scaffold 120 and 405 genes on scaffold 80. They include 13 pseudogenes with internal stop codons or frame shifts, 1 putative tRNA gene (tRNA-Tyr on scaffold 80), and one non-coding RNA gene containing an intron. There are 52 genes with prokaryotic and 78 with eukaryotic ancestry. The remainder are derived from nucleo-cytoplasmic large DNA viruses (NCLDV) including members of Phycodnaviridae (73 genes), Mimiviridae (20 genes), bacteriophages (8 genes), Poxviridae (3 genes), Herpesviridae (1 gene) and Ascoviridae (1 gene). The low-GC region encodes the five known universal proteins of NCLDVs, namely the major capsid protein (genes “036”), primase-helicase (VV D5), family B DNA polymerase (VV E9), packaging ATPase (VV A32), and a transcription factor (VV A2). NCLDV form a monophyletic class encompassing at least seven viral families, including Phycodnaviridae, Mimiviridae, Marseilleviridae, Poxviridae, Ascoviridae, Iridoviridae and Asfarviridae [1], as well as the recently discovered giant Pandoraviruses and Pithoviruses [2, 3]. A phylogenetic analysis of DNA polymerase proteins, a reference marker for DNA virus phylogeny, places the *Asterochloris* virus within the phycodnavirus clade (Phylogram below), in a sister position to the chlorovirus and prasinovirus that infect other green algae (*Chlorella* species and mamiellophytes respectively).


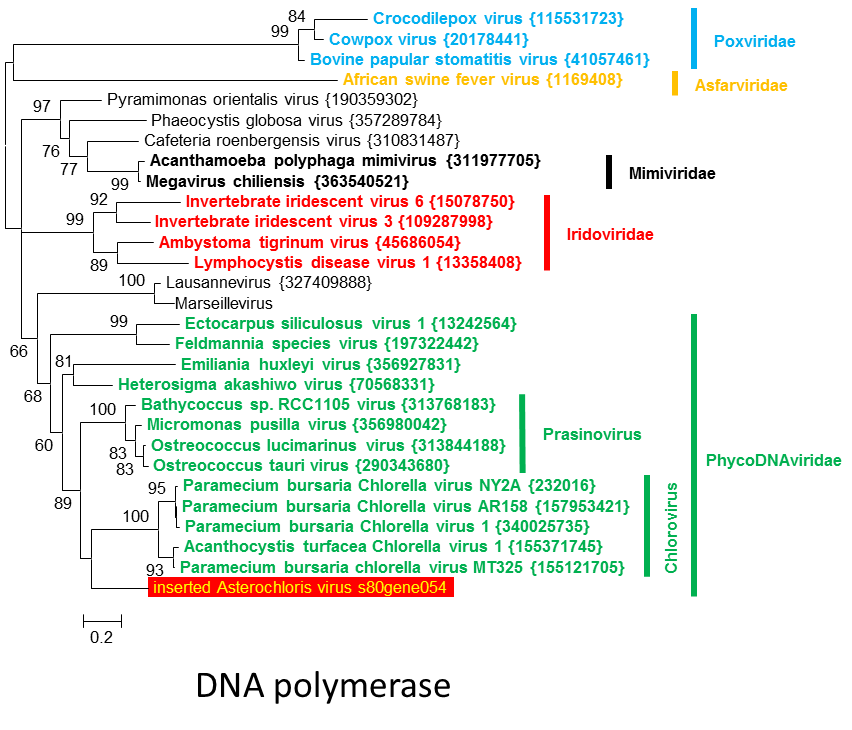


**Phylogram for the DNA polymerase of the *Asterochloris* virus**

The *Asterochloris* virus polymerase (boxed in red) clusters with the polymerases of Phycodnaviridae infecting Chlorophytes. The corresponding gene and protein I.D. are Aster-02261 and 5733, respectively [4]. The homologous protein set for phylogenetic analysis was constructed using the BLAST-EXPLORER [5] website. Proteins were aligned using MUSCLE [6], and amino-acid positions in multiple-alignments containing >30% gaps were removed. The best fitting substitution model (i.e., LG+Γ+I) for the aligned sequence dataset was determined using PROTTEST [7]. ML phylogenetic reconstruction was performed using PhyML [8]. Bootstrap support values label internodes. Scale indicates amino acid substitutions per site.

**References**

1. Iyer LA, Balaji S, Koonin EV, Aravind L: **Evolutionary genomics of nucleo-cytoplasmic large DNA viruses**. *Virus Res* 2006, **117**(1):156-184.

2. Philippe N, Legendre M, Doutre G, Coute Y, Poirot O, Lescot M, Arslan D, Seltzer V, Bertaux L, Bruley C *et al*: **Pandoraviruses: Amoeba Viruses with Genomes Up to 2.5 Mb Reaching That of Parasitic Eukaryotes**. *Science* 2013, **341**(6143):281-286.

3. Legendre M, Bartoli J, Shmakova L, Jeudy S, Labadie K, Adrait A, Lescot M, Poirot O, Bertaux L, Bruley C *et al*: **Thirty-thousand-year-old distant relative of giant icosahedral DNA viruses with a pandoravirus morphology**. *P Natl Acad Sci USA* 2014, **111**(11):4274-4279.

4. Institute DJG. ***Asterochloris sp.* Cgr/DA1pho v2.0.** Available from: <https://genome.jgi.doe.gov/Astpho2/Astpho2.home.html>. Accessed August 8 2018.

5. Dereeper A, Audic S, Claverie JM, Blanc G: **BLAST-EXPLORER helps you building datasets for phylogenetic analysis**. *Bmc Evol Biol* 2010, **10**:8.

6. Edgar RC: **MUSCLE: multiple sequence alignment with high accuracy and high throughput**. *Nucleic acids research* 2004, **32**(5):1792-1797.

7. Abascal F, Zardoya R, Posada D: **ProtTest: selection of best-fit models of protein evolution**. *Bioinformatics* 2005, **21**(9):2104-2105.

8. Guindon S, Dufayard JF, Lefort V, Anisimova M, Hordijk W, Gascuel O: **New Algorithms and Methods to Estimate Maximum-Likelihood Phylogenies: Assessing the Performance of PhyML 3.0**. *Syst Biol* 2010, **59**(3):307-321.
