# Supplementary material for: The lichen symbiosis re-viewed through the genomes of Cladonia grayi and its algal partner Asterochloris glomerata
Source: BMC Genomics. 2019 Jul 23;20:605. doi: 10.1186/s12864-019-5629-x (PMC6652019; doi:10.1186/s12864-019-5629-x)
Supplement: Supplementary file 5 — Sexual reproduction and candidate genes. (ZIP 182 kb) [file 12864_2019_5629_MOESM5_ESM.zip › Additional file 5/Additional file 5_1.Sexual reproduction.docx]

**Additional file 5_1**

**Sexual reproduction in mycobiont and photobiont**

We used the protein sequences from the *C. grayi MAT* locus as queries in TBLASTN searches to probe the organization of the *MAT* loci in *C. macilenta* and *C. metacorallifera*, whose whole genome shotgun sequences from single spore isolates are in the NCBI database [1, 2]. Both isolates happen to be *MAT1-1*, and the structure of their loci is almost identical to that of *C. grayi's MAT1-1* (Fig. 4A). Like other heterothallic mating loci, the *C. grayi* loci show synteny around *Apn2* and *Sla2* but no similarity in the internal region where the idiomorphs are located. However, in *C. grayi*, a 3' end segment of *MAT1-1* protrudes into the syntenic region on the *Sla2* side and a 3' end segment of *MAT1-2* protrudes into the syntenic region on the *Apn2* side (Fig. 4A). This arrangement suggest that today's heterothallic arrangement in *Cladonia* evolved through two alternative deletions removing the edges of an ancestral homothallic locus containing one copy each of *MAT1-1,* *MAT1-2* and *MAT1-1-7* (Fig. 4B).

The figure below lists RNA expression levels (in RPKM, see Methods) in monoculture (Additional file 6) for 13 *A. glomerata* genes (the *Arabidopsis thaliana* nomenclature is used here), most of whose orthologs are thought to operate in meiosis and some in both meiosis and mitosis (*Rad51*, *Syn4*, *Spo11-2*, *Spo11-3*, *Syn1*, *Dmc1*, *Asy1*, *Ahp2*, *Mnd1*, *Pch2*, *Mer3*, *Msh4*, *Msh5*) [3-12]. The expression levels of seven of these genes is about 0.1 of the median expression of all *Asterochloris* genes, while six are in the median range (Figure below). These mostly very low expression levels are consistent with the rare occurrence of sexual reproduction in this alga in nature. *Asterochloris* does not appear to have additional paralogs found in *Arabidopsis* (*Syn2*, *Syn3*, *Spo11-1*); however, these are not *Asterochloris*-specific losses because the two other trebouxoid algae we examined by BLAST [13], *Coccomyxa* C169 [14] and *Chlorella* NC64A [15], also had the same set of 13 genes found and 3 undetected.

A

| ***Arabidopsis*** | ***Asterochloris*** | **Expression** |
| --- | --- | --- |
| **names** | **names** | **(RPKM)** |

| Dmc1 | Aster-x0476 | 3.21257355 |
| --- | --- | --- |
| Ahp2 | Aster-04707 | 3.531771563 |
| Rad51 | Aster-x0963 | 4.136974574 |
| Mer3 | Aster-04262 | 5.067929759 |
| Msh4 | Aster-00547 | 5.25523616 |
| Msh5 | Aster-07039 | 6.097874276 |
| Syn1 | Aster-08167 | 7.344790615 |
| Pch2 | Aster-06511 | 13.81025186 |
| Syn4 | Aster-03199 | 18.78260331 |
| Spo11-3 | Aster-00389 | 43.64193123 |
| Mnd1 | Aster-06098 | 47.02607348 |
| Asy1 | Aster-x0647 | 58.76428522 |
| Spo11-2 | Aster-02518 | 108.2021741 |

B


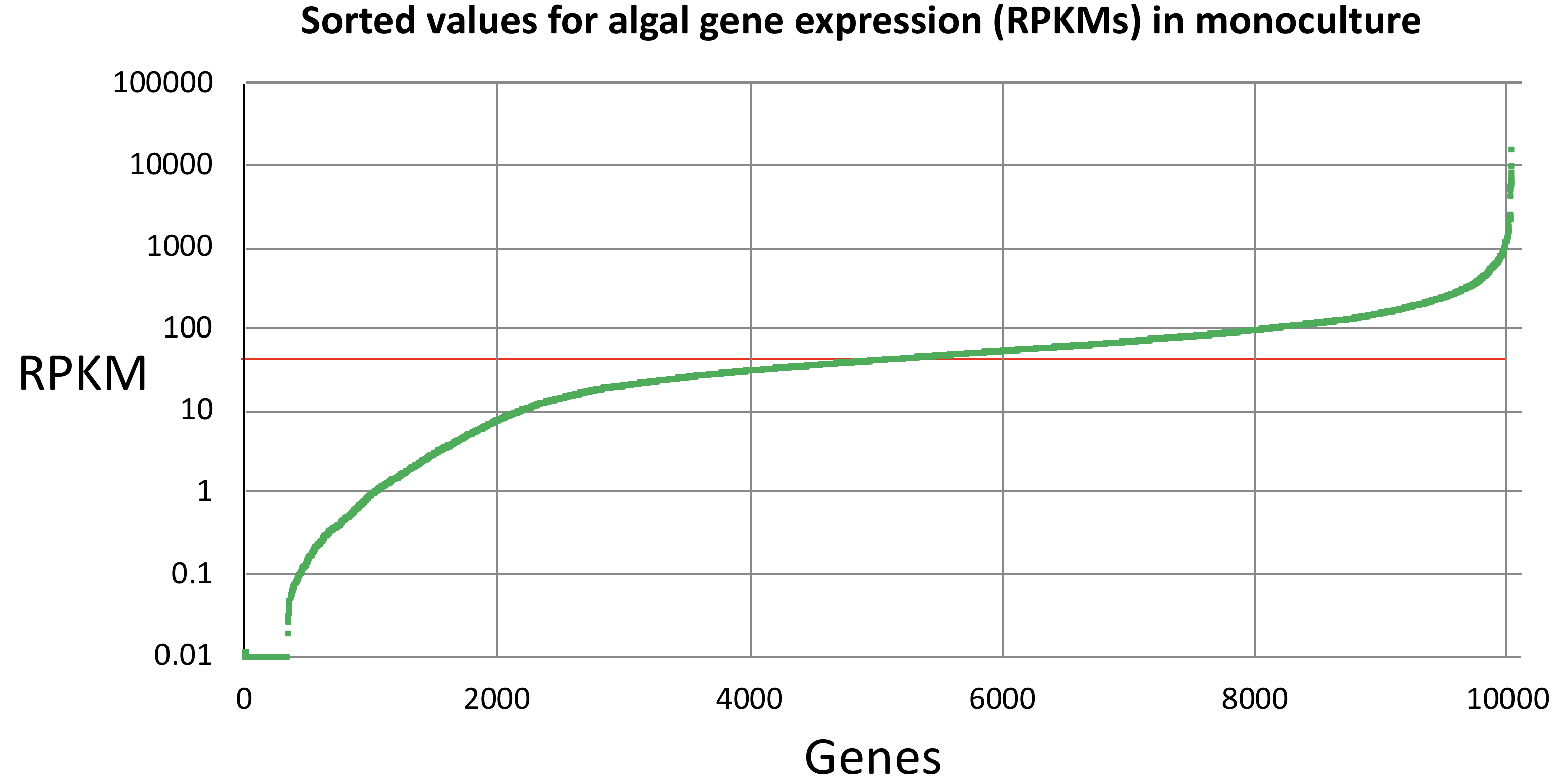


***Asterochloris* meiosis genes and their expression.**

**A:** List of meiosis-specific genes and select paralogs identified in *Asterochloris*. Details on gene identification and expression measures are in Methods. **B:** The green line represents the expression values for all measured *A. glomerata* genes; the red line marks the median expression. Expression for 7 of the 13 genes in panel A is around 1/10 of the median.

**References**

1. Park SY, Choi J, Kim JA, Jeong MH, Kim S, Lee YH, Hur JS: **Draft Genome Sequence of *Cladonia macilenta* KoLRI003786, a Lichen-Forming Fungus Producing Biruloquinone**. *Genome announcements* 2013, **1**(5).

2. Park SY, Choi J, Lee GW, Kim JA, Oh SO, Jeong MH, Yu NH, Kim S, Lee YH, Hur JS: **Draft Genome Sequence of Lichen-Forming Fungus *Cladonia metacorallifera* Strain KoLRI002260**. *Genome announcements* 2014, **2**(1).

3. Schubert V, Weissleder A, Ali H, Fuchs J, Lermontova I, Meister A, Schubert I: **Cohesin gene defects may impair sister chromatid alignment and genome stability in *Arabidopsis thaliana***. *Chromosoma* 2009, **118**(5):591-605.

4. Stacey NJ, Kuromori T, Azumi Y, Roberts G, Breuer C, Wada T, Maxwell A, Roberts K, Sugimoto-Shirasu K: ***Arabidopsis* SPO11-2 functions with SPO11-1 in meiotic recombination**. *Plant Journal* 2006, **48**(2):206-216.

5. Sugimoto-Shirasu K, Stacey NJ, Corsar J, Roberts K, McCann MC: **DNA topoisomerase VI is essential for endoreduplication in *Arabidopsis***. *Curr Biol* 2002, **12**(20):1782-1786.

6. Lambing C, Osman K, Nuntasoontorn K, West A, Higgins JD, Copenhaver GP, Yang J, Armstrong SJ, Mechtler K, Roitinger E *et al*: ***Arabidopsis* PCH2 Mediates Meiotic Chromosome Remodeling and Maturation of Crossovers**. *Plos Genet* 2015, **11**(7).

7. Chen CB, Zhang W, Timofejeva L, Gerardin Y, Ma H: **The *Arabidopsis* ROCK-N-ROLLERS gene encodes a homolog of the yeast ATP-dependent DNA helicase MER3 and is required for normal meiotic crossover formation**. *Plant Journal* 2005, **43**(3):321-334.

8. Higgins JD, Armstrong SJ, Franklin FCH, Jones GH: **The *Arabidopsis* MutS homolog AtMSH4 functions at an early step in recombination: evidence for two classes of recombination in *Arabidopsis***. *Gene Dev* 2004, **18**(20):2557-2570.

9. Kerzendorfer C, Vignard J, Pedrosa-Harand A, Siwiec T, Akimcheva S, Jolivet S, Sablowski R, Armstrong S, Schweizer D, Mercier R *et al*: **The *Arabidopsis thaliana* MND1 homologue plays a key role in meiotic homologous pairing, synapsis and recombination**. *J Cell Sci* 2006, **119**(12):2486-2496.

10. Liu JJ, Qu LJ: **Meiotic and mitotic cell cycle mutants involved in gametophyte development in *Arabidopsis***. *Mol Plant* 2008, **1**(4):564-574.

11. Lu XD, Liu XL, An LZ, Zhang W, Sun J, Pei HJ, Meng HY, Fan YL, Zhang CY: **The *Arabidopsis* MutS homolog AtMSH5 is required for normal meiosis**. *Cell Res* 2008, **18**(5):589-599.

12. Ross KJ, Fransz P, Armstrong SJ, Vizir I, Mulligan B, Franklin FCH, Jones GH: **Cytological characterization of four meiotic mutants of *Arabidopsis* isolated from T-DNA-transformed lines**. *Chromosome Res* 1997, **5**(8):551-559.

13. Altschul SF, Madden TL, Schaffer AA, Zhang J, Zhang Z, Miller W, Lipman DJ: **Gapped BLAST and PSI-BLAST: a new generation of protein database search programs**. *Nucleic acids research* 1997, **25**(17):3389-3402.

14. Blanc G, Agarkova I, Grimwood J, Kuo A, Brueggeman A, Dunigan DD, Gurnon J, Ladunga I, Lindquist E, Lucas S *et al*: **The genome of the polar eukaryotic microalga *Coccomyxa subellipsoidea* reveals traits of cold adaptation**. *Genome biology* 2012, **13**(5):R39.

15. Blanc G, Duncan G, Agarkova I, Borodovsky M, Gurnon J, Kuo A, Lindquist E, Lucas S, Pangilinan J, Polle J *et al*: **The *Chlorella variabilis* NC64A Genome Reveals Adaptation to Photosymbiosis, Coevolution with Viruses, and Cryptic Sex**. *Plant Cell* 2010, **22**(9):2943-2955.
