# Supplementary material for: The lichen symbiosis re-viewed through the genomes of Cladonia grayi and its algal partner Asterochloris glomerata
Source: BMC Genomics. 2019 Jul 23;20:605. doi: 10.1186/s12864-019-5629-x (PMC6652019; doi:10.1186/s12864-019-5629-x)
Supplement: Supplementary file 6 — In vitro culture and gene expression. (ZIP 1530 kb) [file 12864_2019_5629_MOESM6_ESM.zip › Additional file 6/Additional file 6_1.In vitro culture and gene expression.docx]

**Additional file 6_1**

***In vitro* culture and gene expression**

**Effect of algal overgrowth**

After 21 days of growth on filters, mycobiont and photobiont are forming mixed and poorly differentiated aggregates (we refer to them as "lichenoids" [1]) that do not differentiate further under these culture conditions [2]. Although we and others assume that these stages resemble natural early stages of lichenization [2, 3], this remains undemonstrated due to the lack of further *in vitro* development. The RNA was sequenced and the ratio of day-21 coculture to day-21 monoculture RPKM (Reads Per Kilobase per Million reads, calculated using BowStrap [4]) was taken to represent each gene’s expression shift in coculture. For the genes that changed expression, the average shift was about 10x smaller in the alga than in the fungus. We do not ascribe this to a fundamental difference in the way fungus and alga modulate their transcriptional responses but rather to faster growth of the alga in coculture relative to monoculture. Algal cells visibly overgrew the fungus in coculture and many were not in direct contact with hyphae, while most hyphae were in contact with algae. It is therefore likely that all fungal transcripts in the RNA extracted from cocultures were from hyphae reacting to nearby algae, whereas transcripts from the algal cells contacting the fungus were diluted by those from the more abundant cells not contacting it. Therefore, the algal differential expression values appear weaker because only a fraction of the algal population was reacting to the presence of the fungus in coculture.

**Description of individual fungal proteins induced in coculture
A.** CLAGR_010764-RA is induced more than 100-fold. This gene encodes a KP4-like killer toxin similar to that produced by an obligate intracellular virus in some *Ustilago maydis* strains [5]. The *U. maydis* toxin is a small secreted protein shown to inhibit Ca^++^ channels and associated signal transduction events in sensitive *U. maydis* strains [6]. *In vitro*, it can inhibit the Ca^++^ channels of mammalian cells [7]. The putative *Cladonia* homolog is genome-encoded, has a secretion signal, and is among the smallest (192 AA) and most highly induced proteins in our system. Although its function is unknown, we speculate that it may target algal Ca^++^-based signaling, central in plant and algal responses to developmental and environmental cues [8]. In relation to symbiosis, cytosolic and nuclear [Ca^++^] oscillations are known to mediate the responses of plant root cells to AM fungi and to *Rhizobium* in what is called the common symbiotic signaling pathway [9, 10]. The involvement of Ca^++^ signaling in early mycobiont-photobiont interactions is also suggested by the 2-fold upregulation of CLAGR_000113-RA, a putative calcium transporter related to FIG1 from *S. cerevisiae* [11]. Although not induced in coculture, another putative fungal Ca^++^ channel, encoded by CLAGR_002836-RA, is one of the slow evolving proteins discussed in Slow-evolving proteins and anti-stress strategies in the mycobiont section. The potential symbiotic relevance of these Ca^++^ flux proteins has at least another parallel in the alga (see the description below of induced algal proteins.) **B.** CLAGR_008646-RA and CLAGR_010932-RA, induced 10- and 4-fold respectively, encode two lectins. It has been postulated for a long time that the ability of mycobiont lectins to bind specifically to selected carbohydrate moieties in the photobiont cell wall may play a central role in lichen symbiotic interactions [12]. Very high differential expression of mycobiont lectin *lec1* was seen in the symbiotic tissue of the lichen *Peltigera membranacea* [13]. During lichen heat stress, expression changes of paralogs *lec1* and *lec2* correlated with expression changes in genes of the cyanobacterial photobiont of *P. membrancea* [14]. Fungal lectins appear to be involved also in the EM symbiosis [15]. **C.** CLAGR_011186-RA, induced 2-fold, CLAGR_002910-RA, induced 4-fold, and CLAGR_002710-RA, induced 2 fold, encode respectively a Gα subunit, a regulator of Gα signaling, and a dual specificity phosphatase, central components of MAPK signal transduction pathways (Additional file 8). All three belong to expanded families identified through a specific survey of signal transduction components (Specific survey of mycobiont and photobiont signal transduction components section and Additional file 8). A phylogeny of Gα subunits (Fig. 13) identifies CLAGR_011186-RA as a member of a unique expansion of MAG C-like Gα subunits. Coculture induction and family expansion point to these proteins as promising targets of future investigations into specific symbiosis signaling pathways. **D.** CLAGR_007359-RA, induced 3-fold, encodes a homolog of *Dim-2*, a DNA methyltransferase responsible for methylation at the C5 position of selected cytosines in the ascomycete *Neurospora crassa* [16]*.* Earlier experiments involving methylation-sensitive restriction analysis and Southern blots of specific *C. grayi* genes showed higher levels of DNA methylation in the lichenized than in the non-lichenized mycobiont [17]. This suggested that epigenetic re-programming may be associated with symbiosis in *Cladonia*. The induction of the *Dim-2* homolog CLAGR_007359-RA during early fungus-alga interaction is consistent with this possibility. **E.** Carbon is transferred from trebouxoid algae to the mycobiont as ribitol [18], a polyol sugar, and a putative ribitol transporter has been identified in the lichen *Ramalina conduplicans* [19]. We queried the *C. grayi* proteome with the sequences of two functionally validated D-sorbitol/D-mannitol/ribitol transporters from the ascomycetous yeast *Debaryomyces hansenii* [20[20] and checked the Co/Mo expression ratios of the top five hits. Only one of the five genes, CLAGR_004844-RA, was induced (2.6-fold) (Fig. 10A). Like many sugar transporters, the protein has twelve predicted transmembrane domains and characteristic consensus motifs [21, 22] (Fig. 10B). In a protein phylogeny with 37 other fungal putative sugar transporters, the five *C. grayi* proteins are polyphyletic; CLAGR_004844-RA falls in a small clade diverging early, right after the divergence of the yeast orthologs (Fig. 10C), suggesting an ancient origin for this transporter. Induction in coculture, structure, and phylogeny predict that CLAGR_004844-RA encodes a ribitol transporter specific for the symbiosis. In all fungi, polyols not only are sources of metabolic carbon but also appear to be involved in protection from osmotic stress [23, 24]. There are many more sugar transporters in *C. grayi*, and most are part of the contracted protein family Fam_1 (see Mycobiont contracted families section), but CLAGR_004844-RA and the other four discussed here are not part of Fam_1. See Inferences from differential transcription about nutritional fluxes at the symbiotic interface section for further details on individual genes that could be involved in symbiotic carbon and nitrogen transfer.

**Description of individual algal proteins induced in coculture**

**A.** Most highly induced (73-fold) is Aster-04252, a gene encoding a thioredoxin, a small protein from a ubiquitous and multifunctional family essential for disulfide bond regulation and redox control in all organisms [25]. One of thioredoxin's primary roles is to maintain the redox cycle of ribonucleotide reductase to produce deoxyribonucleotides from ribonucleotides. Thus, its activation in coculture could be part of the activation of algal DNA synthesis and cell division mentioned in relation to the data in Fig. 8. However, as a principal actor in redox defense in plants [26], thioredoxin induction also could be part of the alga's defenses against the dehydration and rehydration stresses that characterize the symbiosis [27]. (During dehydration stress, the lichen fungus *Endocarpon pusillum* induces several thioredoxin genes [28, 29], but *C. grayi* did not induce thioredoxins in our coculture system). **B.** The alga induces 2.3-fold Aster-01625, encoding a kinesin motor domain that also surfaces as one of a few "fast-evolvers" (Additional file 11). Kinesins hydrolyze ATP to power intracellular movement along microtubule tracks, and are central in vesicle and protein trafficking [30]. Thus, the induction of Aster-01625 correlates with the apparent increase in algal protein trafficking (Fig. 8). **C.** Slightly above the algal induction threshold are Aster-01936 and Aster-06761, induced 1.7- and 1.5-fold respectively, that encode two secreted proteins with fasciclin domains. Fasciclin domain proteins are found extracellularly in animals, plants, algae, and prokaryotes and are mediators of cell-cell adhesion [31, 32]. A fasciclin-domain protein was 5-10-fold more abundant in the cyanobacterial photobiont freshly isolated from the lichen *Peltigera membranacea* than in free-living isolates of the same cyanobacterium [33]. Lichen photobionts, whether *Cladonia* green algae or *Peltigera* cyanobacteria (*Nostoc*), aggregate to form the photobiont layer and other symbiotic structures in natural lichens or in coculture. It is therefore possible that the Aster-01936 and Aster-06761 gene products are involved in algal aggregation processes occurring early in the *Cladonia-Asterochloris* interaction. **D.** Finally Aster-03695, induced 1.31-fold, is an 11-transmembrane domain protein similar to *Arabidopsis* *ERD-4* (early response to dehydration) proteins. One of these, *OSCA1*, has been characterized as a hyperosmolality-gated Ca^++^ channel involved in drought protection [34]. As mentioned in the above description of induced fungal proteins, Ca^++^ is central to algal signaling, and the importance of the lichen's responses to dehydration is highlighted by several other algal and fungal genes identified by our screens.

**References**

1. Armaleo D, May S: **Sizing the fungal and algal genomes of the lichen *Cladonia grayi* through quantitative PCR**. *Symbiosis* 2009, **49**(1):43-51.

2. Joneson S, Armaleo D, Lutzoni F: **Fungal and algal gene expression in early developmental stages of lichen-symbiosis**. *Mycologia* 2011, **103**(2):291-306.

3. Athukorala SN, Huebner E, Piercey-Normore MD: **Identification and comparison of the 3 early stages of resynthesis for the lichen *Cladonia rangiferina***. *Canadian journal of microbiology* 2014, **60**(1):41-52.

4. Larsen PE, Collart FR: **BowStrap v1.0: Assigning statistical significance to expressed genes using short-read transcriptome data**. *BMC Research Notes* 2012, **5**:275.

5. Park CM, Bruenn JA, Ganesa C, Flurkey WF, Bozarth RF, Koltin Y: **Structure and Heterologous Expression of the *Ustilago maydis* Viral Toxin Kp4**. *Mol Microbiol* 1994, **11**(1):155-164.

6. Gage MJ, Bruenn J, Fischer M, Sanders D, Smith TJ: **KP4 fungal toxin inhibits growth in *Ustilago maydis* by blocking calcium uptake**. *Mol Microbiol* 2001, **41**(4):775-785.

7. Gu F, Khimani A, Rane SG, Flurkey WH, Bozarth RF, Smith TJ: **Structure and Function of a Virally Encoded Fungal Toxin from *Ustilago maydis* - a Fungal and Mammalian Ca2+ Channel Inhibitor**. *Structure* 1995, **3**(8):805-814.

8. Wheeler GL, Brownlee C: **Ca2+ signalling in plants and green algae - changing channels**. *Trends Plant Sci* 2008, **13**(9):506-514.

9. Bonfante P, Genre A: **Mechanisms underlying beneficial plant-fungus interactions in mycorrhizal symbiosis**. *Nat Commun* 2010, **1**:48.

10. Parniske M: **Arbuscular mycorrhiza: the mother of plant root endosymbioses**. *Nat Rev Microbiol* 2008, **6**(10):763-775.

11. Muller EM, Mackin NA, Erdman SE, Cunningham KW: **Fig1p facilitates Ca2+ influx and cell fusion during mating of *Saccharomyces cerevisiae***. *Journal of Biological Chemistry* 2003, **278**(40):38461-38469.

12. Singh RS, Walia AK: **Characteristics of lichen lectins and their role in symbiosis**. *Symbiosis* 2014, **62**(3):123-134.

13. Miao VPW, Manoharan SS, Snaebjarnarson V, Andresson OS: **Expression of lec-1, a mycobiont gene encoding a galectin-like protein in the lichen *Peltigera membranacea***. *Symbiosis* 2012, **57**(1):23-31.

14. Steinhauser SS, Andresson OS, Palsson A, Werth S: **Fungal and cyanobacterial gene expression in a lichen symbiosis: Effect of temperature and location**. *Fungal Biol-Uk* 2016, **120**(10):1194-1208.

15. Giollant M, Guillot J, Damez M, Dusser M, Didier P, Didier E: **Characterization of a Lectin from *Lactarius deterrimus* - Research on the Possible Involvement of the Fungal Lectin in Recognition between Mushroom and Spruce during the Early Stages of Mycorrhizae Formation**. *Plant Physiol* 1993, **101**(2):513-522.

16. Kouzminova E, Selker EU: **dim-2 encodes a DNA methyltransferase responsible for all known cytosine methylation in *Neurospora***. *Embo J* 2001, **20**(15):4309-4323.

17. Armaleo D, Miao V: **Symbiosis and DNA methylation in the *Cladonia* lichen fungus**. *Symbiosis* 1999, **26**(2):143-163.

18. Richardson DHS, Jackson Hill, D., Smith, D.C.: **Lichen Physiology XI. The Role of the Alga in Determining the Pattern of Carbohydrate Movement Between Lichen Symbionts**. *The New phytologist* 1968, **67**:469-486.

19. Yoshino K. SM, Sakamoto K., Yamamoto Y.: **Candidates of ribitol transporter gene obtained from *Ramalina conduplicans***. In*.* Abstract Book, 8th IAL Symposium, Poster: University of Helsinki; 2016: 116.

20. Pereira I, Madeira A, Prista C, Loureiro-Dias MC, Leandro MJ: **Characterization of New Polyol/H+ Symporters in *Debaryomyces hansenii***. *Plos One* 2014, **9**(2):e88180.

21. Leandro MJ, Fonseca C, Goncalves P: **Hexose and pentose transport in ascomycetous yeasts: an overview**. *Fems Yeast Res* 2009, **9**(4):511-525.

22. Gao ZF, Maurousset L, Lemoine R, Yoo SD, van Nocker S, Loescher W: **Cloning, expression, and characterization of sorbitol transporters from developing sour cherry fruit and leaf sink tissues**. *Plant Physiol* 2003, **131**(4):1566-1575.

23. Kranner I, Beckett R, Hochman A, Nash TH: **Desiccation-tolerance in lichens: a review**. *Bryologist* 2008, **111**(4):576-593.

24. Ruijter GJG, Visser J, Rinzema A: **Polyol accumulation by *Aspergillus oryzae* at low water activity in solid-state fermentation**. *Microbiol-Sgm* 2004, **150**:1095-1101.

25. Arner ESJ, Holmgren A: **Physiological functions of thioredoxin and thioredoxin reductase**. *European Journal of Biochemistry* 2000, **267**(20):6102-6109.

26. Dos Santos CV, Rey P: **Plant thioredoxins are key actors in the oxidative stress response**. *Trends Plant Sci* 2006, **11**(7):329-334.

27. Honegger R: **The symbiotic phenotype of lichen-forming ascomycetes and their endo- and epibionts. In: Hock, Bertold. Fungal Associations. Berlin, Heidelberg, 287-339. ISBN 978-3-642-30825-3.**, vol. 9. Berlin, Heidelberg: Springer; 2012.

28. Wang YY, Zhang XY, Zhou QM, Zhang XL, Wei JC: **Comparative transcriptome analysis of the lichen-forming fungus *Endocarpon pusillum* elucidates its drought adaptation mechanisms**. *Sci China Life Sci* 2015, **58**(1):89-100.

29. Li H, Wei JC: **Functional analysis of thioredoxin from the desert lichen-forming fungus, *Endocarpon pusillum* Hedwig, reveals its role in stress tolerance**. *Sci Rep-Uk* 2016, **6**.

30. Hirokawa N, Noda Y, Tanaka Y, Niwa S: **Kinesin superfamily motor proteins and intracellular transport**. *Nat Rev Mol Cell Bio* 2009, **10**(10):682-696.

31. Huber O, Sumper M: **Algal-Cams - Isoforms of a Cell-Adhesion Molecule in Embryos of the Alga *Volvox* with Homology to *Drosophila* Fasciclin-I**. *Embo J* 1994, **13**(18):4212-4222.

32. Johnson KL, Jones BJ, Bacic A, Schultz CJ: **The fasciclin-like arabinogalactan proteins of *Arabidopsis*. A multigene family of putative cell adhesion molecules**. *Plant Physiol* 2003, **133**(4):1911-1925.

33. Paulsrud P, Lindblad P: **Fasciclin domain proteins are present in *Nostoc* symbionts of lichens**. *Appl Environ Microb* 2002, **68**(4):2036-2039.

34. Yuan F, Yang HM, Xue Y, Kong DD, Ye R, Li CJ, Zhang JY, Theprungsirikul L, Shrift T, Krichilsky B *et al*: **OSCA1 mediates osmotic-stress-evoked Ca2+ increases vital for osmosensing in *Arabidopsis***. *Nature* 2014, **514**(7522):367-+.
