# Supplementary material for: The lichen symbiosis re-viewed through the genomes of Cladonia grayi and its algal partner Asterochloris glomerata
Source: BMC Genomics. 2019 Jul 23;20:605. doi: 10.1186/s12864-019-5629-x (PMC6652019; doi:10.1186/s12864-019-5629-x)
Supplement: Supplementary file 7 — Expanded and contracted families. (ZIP 220 kb) [file 12864_2019_5629_MOESM7_ESM.zip › Additional file 7/Additional file 7_1.Expanded and contracted families.docx]

**Additional file 7_1**

**Expanded and contracted families**

**Mycobiont expanded families and contracted transportome**

They are listed in Additional files 7_2 and 7_3. **A.** HET incompatibility proteins (containing HET domains) populate expanded families 11, 12, 13 and 362, with a total of 204 members 13 of which are also induced in coculture. HET incompatibility proteins are involved in preventing somatic fusions between genetically different hyphae of the same fungus [1]. Why would HET gene families be expanded in *Cladonia* and 6% of them be induced in coculture with the alga? We speculate that this may reflect the need for an efficiently lichenizing genotype to prevent other genotypes from lichenizing with its alga through hyphal fusions. Such a role for some of the mycobiont HET genes could be a variant of one of the proposed functions of heterokaryon incompatibility in fungi in general: defense against resource plundering [2]. HET domain proteins are common in Ascomycota. After their discovery in the ectomycorrhizal basidiomycete *Laccaria bicolor,* where they represent the largest gene family [3], HET proteins have been found to be common also in Basidiomycota [4]. **B.** Ankyrin domain (ANK) proteins constitute the next largest *Cladonia* expanded group, centered on families 3 and 872, with 156 members total. ANK proteins are expanded in the photobiont as well (Photobiont expanded families section). ANK domains form protein-protein interaction surfaces [5] and are widely distributed among diverse proteins in eukaryotes and prokaryotes. They are strongly expanded also in the ectomycorrhyzal basidiomycete *Laccaria bicolor* [3] but depleted in the ectomycorrhyzal ascomycete *Tuber melanosporum* [6] (the Périgord black truffle) and in the mutualistic root symbiont *Piriformospora indica* [7] (now *Serendipita indica* [8]). Expansion of ANK-domain proteins has been observed in obligate intracellular or facultative animal-associated bacteria relative to free-living bacteria, suggesting a functional link to those symbioses [9]. The secretion and transfer of proteins from one symbiont to the other [9, 10] or the as yet undocumented but likely extracellular interaction of secreted proteins at the symbiotic boundary [11-13] may perhaps connect symbiosis to expansion of ANK protein families in prokaryotes and eukaryotes. However, such tentative association needs functional verification on a broad scale, especially in view of the depletion of ANK proteins in *T. melanosporum* and *S. indica*. **C.** The analysis circumscribed to the 458 classifiable *C. grayi* membrane transporters shows an overall contraction of the *C. grayi* transportome. Within that context, however, the 9-member L-type Amino Acid Transporter family (LAT) appears slightly expanded. Characterized genes belonging to the fungal LAT family are the two *S. cerevisiae genes MUP1* and *MUP3*, encoding respectively a high-affinity and a low-affinity methionine permease [14, 15]. If some of the *C. grayi* LAT genes are also involved in methionine transport, their expansion might support enhanced methionine import to methylate the large quantities of secondary metabolites it produces in the lichen [16] and to methylate DNA, possibly as part of symbiotic reprogramming of transcription [17]. As mentioned in Additional file 6_1, one of the genes induced in coculture is the *Cladonia* homolog of the *Neurospora* Dim-2 DNA methyltransferase. It is not known whether the alga is a source of methionine for the mycobiont, but both partners have the capacity to synthesize methionine: the mycobiont has two copies and the photobiont one copy of cobalamine-independent methionine synthase. **D.** Expanded family 5 comprises 80 members each with 5 to 7 or more transmembrane helices but is not included in the transportome analysis because Fam_5 members are unknown proteins. Ten of them are induced in coculture. Like the LAT family, Fam_5 also stands in contrast to the overall contraction of the *C. grayi* transportome described below (G.). This atypical expansion and the induction of ten of its members suggest that Fam_5 comprises a class of membrane proteins involved in symbiosis-related signaling and/or transport. **E.** Dramatically expanded Fam_668 comprises seven fructosamine kinases of which three are induced in coculture. Fructosamine kinases [18] are enzymes that reverse the aging-associated protein damage produced by glycation, a spontaneous reaction linking sugars mostly to the ε-amino group of lysine [19]. Studied extensively in mammals, fructosamine kinases are also found in fungal and bacterial genomes [20]. **F.** Expanded family 10 includes polyketide synthases (PKSs), proteins that assemble the carbon backbones of many secondary metabolites. Large numbers of PKSs in lichens had been predicted from the many ketosynthase-domain-encoding fragments amplified by PCR from lichen or mycobiont DNA [21-23]. The last pre-genomic tally had identified 12-13 PKS genes in *C. grayi* [16, 23]. Due to the extensive chemical knowledge on the major lichen secondary metabolites [24, 25] and their debated but central functions [26-28], we analyzed each of the 52 gene models in this family to reduce assembly and annotation errors. The resulting more accurate if not yet definitive estimate (Fig.11 and page 6 in this file) sets the number of complete PKS & NRPS (Non Ribosomal Peptide Synthases) genes in *C. grayi* to 29 with 14 additional but questionable cases. The uncertain cases comprise five likely pseudogenes and nine gene fragments that cannot be reliably defined from the current genome assembly. Our number (29) is close to that (32) tallied from the genome of *Cladonia uncialis* [29] but higher than that in *Lasallia hispanica* (18) and *L. pustulata* (21) [30]. Interestingly, the *Cladonia* numbers place the lichen PKS repertoire on par with that of pathogenic ascomycetes and much higher than that of other mutualists like *Laccaria, Tuber, Rhizophagus,* and *Serendipita* [31]. The mycobiont synthesizes each lichen's known and most abundant extracellularly deposited metabolites [32, 33], yet ascribing specific PKSs to specific compounds has been slow in lichens. Only in two cases, both in Cladoniaceae, has a lichen fungal PKS gene cluster been linked with high likelihood to the corresponding lichen-specific metabolite [16, 29]. The known lichen metabolites are, however, few compared to the large numbers of PKSs in the mycobiont genome, implying a vast and cryptic metabolic potential. This potential is also suggested by the frequent appearance in mycobiont culture of compounds not observed in the natural lichen [33-35]. As algal and cyanobacterial photobionts as well as other prokaryotic and eukaryotic inhabitants of the lichen thallus also have genes for secondary metabolites, the vast majority of trace lichen secondary compounds remains unexplored [36]. The few specific attempts at uncovering trace lichen compounds possibly involved in inter-symbiont communication have so far centered on the algal [37] or cyanobacterial [38] photobiont. **G.** In parallel with Fam_1, Fam_2 and Fam_7, the most significantly contracted families in *C. grayi* included the ATP-binding Cassette (ABC) Superfamily, the Major Facilitator Superfamily (MFS), the Amino acid-Polyamine Organocation (APC) family, Oligopeptide Transporter (OPT) family and the Proton-dependent Oligopeptide Transporter (POT) family. ABC transporter families are contracted also in *S. indica* [7]. APC and MFS families are significantly contracted also in *T. melanosporum* [6], whereas the APC family is expanded in the ectomycorrhizal basidiomycete *L. bicolor* [3].

**Photobiont expanded families**

They are listed in Additional file 7_3. **A.** With 100 members, Fam_16 is the largest expanded photobiont family; it appears unique to *A. glomerata*, and 20% of its members are induced in coculture. From a Fam_16 alignment, a core region of ~ 400 AA overlapping in 21 of the 100 members was selected. Phyre2, a protein structure prediction program [39, 40], matched short computed structures within this core region to known DNA binding proteins in 15 of the 21 sequences tested. Among the top structural matches were the DNA-binding kinetochore protein Ndc10 from *S. cerevisiae* [41] and the structurally related λ-integrase protein family [41], suggesting that some of the Fam_16 proteins may be involved in viral integration events that are prominent in this alga (see A low-GC region in *Asterochloris* is a remnant of a large virus insertion, an HGT-mediator section). Fam_16 members are not concentrated in the viral integration region but are scattered around the genome. A direct symbiotic role for Fam_16 remains undemonstrated but is possible due to this family's uniqueness and to the large fraction of family members induced in coculture. **B.** Kinases are represented by expanded families 40, 248, 264, 1269, with a total of 52 members, suggesting diversification of signal transduction processes in the symbiotic alga (see Additional file 8). **C.** Families 153, 249, and 745, comprising Carbohydrate Active Enzymes (CAZ), are strongly expanded with a total of 40 members, mostly glycosyl transferases. These enzymes are involved in protein glycosylation and cell wall metabolism, affecting the composition of extracellular surfaces. The expansion of these CAZ enzymes may thus extend the range of extracellular structures in a photobiont shuttling through a wide range of diverse contacts, comprising cryptic free-living stages [42-44] and various developmental interactions [45] with one or different mycobiont species [46, 47] under different environmental conditions [46] (see also Conclusions). This variable and subtle sculpting of the algal surfaces might contribute to the difficulty in obtaining reproducible lichen reconstitution *in vitro.* In this regard, it is interesting that cell wall polysaccharides extracted from aposymbiotically-cultured *Asterochloris* from the lichen *Cladina confusa* were not found in the natural *C. confusa* thallus [48]. **D.** *Asterochloris* Fam_20, with 29 members, is an expanded Ankyrin domain protein family. It parallels the Ank-protein expansion seen in the mycobiont and its connection to symbiosis must remain equally tentative. **E.** Fam_137, with its 26 ATPase-like proteins of likely archaeal origin [49], appears to represent a large prokaryotic contribution to *A. glomerata*. The putative archaeal ATPases in *Asterochloris* are dispersed throughout the genome, and none are encoded in the low GC region representing the footprint of a relatively recent viral insertion (see A low-GC region in Asterochloris is a remnant of a large virus insertion, an HGT-mediator section). This makes it unlikely that this particular virus was the HGT mediator for archaeal ATPases. HGT transfer of archaeal ATPases to eukaryotes was documented for the red alga *Galdieria sulphuraria* [50, 51], a unicellular soil extremophile. Our phylogeny (Fig. 12) confirms the *G. sulphuraria* results. It also suggests additional HGT events from Archaea to several eukaryotic lineages. The taxa with archaeal-derived ATPases are scattered among Chlorophyta (*Asterochloris*, *Coccomyxa, Gonium),* Charophyta (*Klebsormidium*), Lycopodiophyta (*Selaginella*), Glomeromycota (*Rhizophagus*) and Basidiomycota (*Puccinia).* Archaeal ATPases are structurally adapted to function under extreme conditions [52], and their gene copy number increases in thermophilic archaea with increasing optimum growth temperature [50]. It was therefore hypothesized that their eukaryotic derivatives could also contribute to thermotolerance [50]. Mostly, the presence of archaea-derived ATPases in eukaryotic taxa appears congruent with this possibility when there are numerous ATPase copies in the genome: the two largest families of archaea-derived ATPases are found in *Asterochloris glomerata* (26 members) and *Galdieria sulphuraria* (12 members), soil algae with tolerance for extreme conditions. The charophytic terrestrial alga *Kelbsormidium flaccidum* (2 members) and the lycopsid *Selaginella moellendorffii* (7 members) are descendants, respectively, of the first land-colonizing algae [53] and of the first vascular plants [54], which had to adapt to dry and warm conditions. Archaeal ATPases may be relevant for the glomeromycete *Rhizophagus irregularis* (5 members), since AM fungi protect plants from drought stress [55]. The significance of archaeal ATPases remains unclear for the not particularly thermotolerant plant pathogens *Puccinia striiformis* (2 members) and *Puccinia triticina* (1 member) and for *Coccomyxa subellipsoidea* C-169 (1 member) and *Gonium pectorale* (1 member), both chlorophycean freshwater algae. No archaeal ATPases are found in the *C. grayi* mycobiont. **F.** A recent transcriptomic study of the cultured lichen alga *Trebouxia gelatinosa* highlights the importance of a family of Desiccation-Related Proteins (DRPs) in the response of *Trebouxia* to desiccation and rehydration, although the mechanisms involved are unclear [56]. Among Chlorophyta, DRP families are thought to have originated from bacteria by HGT [56] and are found only in the lichen algae *Trebouxia* (13 members), *Coccomyxa* (7 members) and *Asterochloris* (where we identified 7 members). Like archaeal ATPases, the *Asterochloris* DRPs are not linked to the viral insertion region described in section 1.2. **G.** Finally, expanded Fam_624 comprises 8 Mg^++^ transporters of unknown role and location. Besides having universal functions in a variety of cellular processes, in plants Mg^++^ is concentrated in chloroplasts where it is essential in photosynthesis, and in vacuoles where it participates in the homeostatic adaptation to changing metabolic and environmental conditions [57, 58].

**List of *C. grayi* PKSs and NRPSs genes** (see also Fig.11)

**Complete PKSs 24**

**Non-Reducing**

CLAGR_002264-RA

CLAGR_010151-RA

CLAGR_005447-RA

CLAGR_001618-RA

CLAGR_011105-RA

CLAGR_001933-RA

CLAGR_002732-RA (Grayanic acid PKS)

CLAGR_007392-RA

CLAGR_003178-RA

**Reducing**

suz_1_CLAGR_005953-RA (formerly 005953 & 005954)

CLAGR_006954-RA

CLAGR_001303-RA

CLAGR_007516-RA

CLAGR_001142-RA

suz_1_CLAGR_010242-RA (formerly 010242 & 010243)

CLAGR_011106-RA

CLAGR_010697-RA

CLAGR_010616-RA

CLAGR_002268-RA

CLAGR_001765-RA

CLAGR_002212-RA

CLAGR_005568-RA

CLAGR_009720-RA

CLAGR_009784-RA (PKS-NRPS hybrid)

**NRPSs 5**

CLAGR_003042-RA

CLAGR_010988-RA

CLAGR_001765-RA

CLAGR_002268-RA

CLAGR_010616-RA

**Almost certainly pseudogenes 5**

suz_1_CLAGR_004544-RA (formerly CLAGR_004546-RA plus CLAGR_004544-RA)

CLAGR_009968-RA linked to CLAGR_009967-RA

CLAGR_010779-RA linked to CLAGR_010780-RA (includes stop codons)

suz_1_CLAGR_007684-RA (formerly from 007684 to 687)

suz_1_CLAGR_001574-RA (formerly from 001572 to 1574)

**Small fragments. If they exist only because of misassembly, they suggest the presence of 9 additional PKSs:**

**Fragments that cannot be joined to other fragments**

CLAGR_006911-RA

CLAGR_010734-RA

CLAGR_001934-RA

CLAGR_001747-RA

CLAGR_002825-RA

**Fragments likely to belong to the same PKS**

CLAGR_010689-RA, CLAGR_010690-RA, CLAGR_011195-RA

CLAGR_006210-RA, CLAGR_006211-RA

CLAGR_007317-RA, CLAGR_007318-RA

CLAGR_006912-RA, CLAGR_007860-RA

**References**

1. Paoletti M, Saupe SJ, Clave C: **Genesis of a Fungal Non-Self Recognition Repertoire**. *Plos One* 2007, **2**(3):e283.

2. Debets AJM, Griffiths AJF: **Polymorphism of het-genes prevents resource plundering in *Neurospora crassa***. *Mycological Research* 1998, **102**:1343-1349.

3. Martin F, Aerts A, Ahren D, Brun A, Danchin EGJ, Duchaussoy F, Gibon J, Kohler A, Lindquist E, Pereda V *et al*: **The genome of *Laccaria bicolor* provides insights into mycorrhizal symbiosis**. *Nature* 2008, **452**(7183):88-U87.

4. Van der Nest MA, Olson A, Lind M, Velez H, Dalman K, Durling MB, Karlsson M, Stenlid J: **Distribution and evolution of het gene homologs in the basidiomycota**. *Fungal Genet Biol* 2014, **64**:45-57.

5. Li JN, Mahajan A, Tsai MD: **Ankyrin repeat: A unique motif mediating protein-protein interactions**. *Biochemistry-Us* 2006, **45**(51):15168-15178.

6. Martin F, Kohler A, Murat C, Balestrini R, Coutinho PM, Jaillon O, Montanini B, Morin E, Noel B, Percudani R *et al*: **Perigord black truffle genome uncovers evolutionary origins and mechanisms of symbiosis**. *Nature* 2010, **464**(7291):1033-1038.

7. Zuccaro A, Lahrmann U, Guldener U, Langen G, Pfiffi S, Biedenkopf D, Wong P, Samans B, Grimm C, Basiewicz M *et al*: **Endophytic life strategies decoded by genome and transcriptome analyses of the mutualistic root symbiont *Piriformospora indica***. *Plos Pathog* 2011, **7**(10):e1002290.

8. Weiss M, Waller F, Zuccaro A, Selosse MA: **Sebacinales - one thousand and one interactions with land plants**. *New Phytologist* 2016, **211**(1):20-40.

9. Jernigan KK, Bordenstein SR: **Ankyrin domains across the Tree of Life**. *PeerJ* 2014, **2**:e264.

10. Lowe RGT, Howlett BJ: **Indifferent, Affectionate, or Deceitful: Lifestyles and Secretomes of Fungi**. *Plos Pathog* 2012, **8**(3):e1002515.

11. Martin F, Nehls U: **Harnessing ectomycorrhizal genomics for ecological insights**. *Curr Opin Plant Biol* 2009, **12**(4):508-515.

12. Bonfante P, Genre A: **Mechanisms underlying beneficial plant-fungus interactions in mycorrhizal symbiosis**. *Nat Commun* 2010, **1**:48.

13. Plett JM, Martin F: **Blurred boundaries: lifestyle lessons from ectomycorrhizal fungal genomes**. *Trends Genet* 2011, **27**(1):14-22.

14. Isnard AD, Thomas D, Surdin-Kerjan Y: **The study of methionine uptake in *Saccharomyces cerevisiae* reveals a new family of amino acid permeases**. *Journal of molecular biology* 1996, **262**(4):473-484.

15. Kosugi A, Koizumi Y, Yanagida F, Udaka S: **MUP1, high affinity methionine permease, is involved in cysteine uptake by *Saccharomyces cerevisiae***. *Biosci Biotech Bioch* 2001, **65**(3):728-731.

16. Armaleo D, Sun XM, Culberson C: **Insights from the first putative biosynthetic gene cluster for a lichen depside and depsidone**. *Mycologia* 2011, **103**(4):741-754.

17. Armaleo D, Miao V: **Symbiosis and DNA methylation in the *Cladonia* lichen fungus**. *Symbiosis* 1999, **26**(2):143-163.

18. Delpierre G, Van Schaftingen E: **Fructosamine 3-kinase, an enzyme involved in protein deglycation**. *Biochemical Society transactions* 2003, **31**(Pt 6):1354-1357.

19. Wautier JL, Schmidt AM: **Protein glycation - A firm link to endothelial cell dysfunction**. *Circ Res* 2004, **95**(3):233-238.

20. Gemayel R, Fortpied J, Rzem R, Vertommen D, Veiga-da-Cunha M, Van Schaftingen E: **Many fructosamine 3-kinase homologues in bacteria are ribulosamine/erythrulosamine 3-kinases potentially involved in protein deglycation**. *Febs J* 2007, **274**(17):4360-4374.

21. Miao V, Coeffet-LeGal MF, Brown D, Sinnemann S, Donaldson G, Davies J: **Genetic approaches to harvesting lichen products**. *Trends Biotechnol* 2001, **19**(9):349-355.

22. Muggia L, Schmitt I, Grube M: **Purifying selection is a prevailing motif in the evolution of ketoacyl synthase domains of polyketide synthases from lichenized fungi**. *Mycological Research* 2008, **112**:277-288.

23. Timsina BA, Hausner G, Piercey-Normore MD: **Evolution of ketosynthase domains of polyketide synthase genes in the *Cladonia chlorophaea* species complex (Cladoniaceae)**. *Fungal Biol-Uk* 2014, **118**(11):896-909.

24. Culberson CF: **Chemical and botanical guide to lichen products**. Chapel Hill,: University of North Carolina Press; 1969.

25. Huneck S: **The significance of lichens and their metabolites**. *Naturwissenschaften* 1999, **86**(12):559-570.

26. Molnar K, Farkas E: **Current Results on Biological Activities of Lichen Secondary Metabolites: a Review**. *Z Naturforsch C* 2010, **65**(3-4):157-173.

27. Armaleo D, Zhang Y, Cheung S: **Light might regulate divergently depside and depsidone accumulation in the lichen *Parmotrema hypotropum* by affecting thallus temperature and water potential**. *Mycologia* 2008, **100**(4):565-576.

28. Deduke C, Timsina B, Piercey-Normore MD: **Effect of Environmental Change on Secondary Metabolite Production in Lichen-Forming Fungi**. *International Perspectives on Global Environmental Change* 2011:197-230.

29. Abdel-Hameed M, Bertrand RL, Piercey-Normore MD, Sorensen JL: **Putative identification of the usnic acid biosynthetic gene cluster by de novo whole-genome sequencing of a lichen -forming fungus**. *Fungal Biol-Uk* 2016, **120**(3):306-316.

30. Dal Grande F, Meiser A, Tzovaras BG, Otte J, Ebersberger I, Schmitt I: **The draft genome of the lichen-forming fungus *Lasallia hispanica* (Frey) Sancho & A. Crespo**. *Lichenologist* 2018, **50**(3):329-340.

31. Zuccaro A, Lahrmann U, Langen G: **Broad compatibility in fungal root symbioses**. *Curr Opin Plant Biol* 2014, **20**:135-145.

32. Culberson CF, Armaleo D: **Induction of a Complete Secondary-Product Pathway in a Cultured Lichen Fungus**. *Exp Mycol* 1992, **16**(1):52-63.

33. Fazio AT, Bertoni MD, Adler MT, Ruiz LB, Rosso ML, Muggia L, Hager A, Stocker-Worgotter E, Maier MS: **Culture studies on the mycobiont isolated from *Parmotrema reticulatum* (Taylor) Choisy: metabolite production under different conditions**. *Mycol Prog* 2009, **8**(4):359-365.

34. Molina MC, Crespo A, Vicente C, Elix JA: **Differences in the composition of phenolics and fatty acids of cultured mycobiont and thallus of *Physconia distorta***. *Plant Physiol Bioch* 2003, **41**(2):175-180.

35. Brunauer G, Hager A, Grube M, Tuerk R, Stocker-Worgotter E: **Alterations in secondary metabolism of aposymbiotically grown mycobionts of *Xanthoria elegans* and cultured resynthesis stages**. *Plant Physiol Bioch* 2007, **45**(2):146-151.

36. Parrot D, Jan S, Baert N, Guyot S, Tomasi S: **Comparative metabolite profiling and chemical study of *Ramalina siliquosa* complex using LC-ESI-MS/MS approach**. *Phytochemistry* 2013, **89**:114-124.

37. Meessen J, Eppenstein S, Ott S: **Recognition mechanisms during the pre-contact state of lichens: II. Influence of algal exudates and ribitol on the response of the mycobiont of *Fulgensia bracteata***. *Symbiosis* 2013, **59**(3):131-143.

38. Kampa A, Gagunashvili AN, Gulder TAM, Morinaka BI, Daolio C, Godejohann M, Miao VPW, Piel J, Andresson OS: **Metagenomic natural product discovery in lichen provides evidence for a family of biosynthetic pathways in diverse symbioses**. *P Natl Acad Sci USA* 2013, **110**(33):E3129-E3137.

39. Jefferys BR, Kelley LA, Sternberg MJ: **Protein folding requires crowd control in a simulated cell**. *Journal of molecular biology* 2010, **397**(5):1329-1338.

40. Kelley LA, Sternberg MJE: **Protein structure prediction on the Web: a case study using the Phyre server**. *Nature protocols* 2009, **4**(3):363-371.

41. Perriches T, Singleton MR: **Structure of Yeast Kinetochore Ndc10 DNA-binding Domain Reveals Unexpected Evolutionary Relationship to Tyrosine Recombinases**. *Journal of Biological Chemistry* 2012, **287**(7):5173-5179.

42. Mukhtar A, Garty J, Galun M: **Does the Lichen Alga *Trebouxia* Occur Free-Living in Nature - Further Immunological Evidence**. *Symbiosis* 1994, **17**(2-3):247-253.

43. Frey B, Buhler L, Schmutz S, Zumsteg A, Furrer G: **Molecular characterization of phototrophic microorganisms in the forefield of a receding glacier in the Swiss Alps**. *Environ Res Lett* 2013, **8**(1):015033.

44. Hedenas H, Blomberg P, Ericson L: **Significance of old aspen (*Populus tremula*) trees for the occurrence of lichen photobionts**. *Biol Conserv* 2007, **135**(3):380-387.

45. Honegger R: **The symbiotic phenotype of lichen-forming ascomycetes and their endo- and epibionts. In: Hock, Bertold. Fungal Associations. Berlin, Heidelberg, 287-339. ISBN 978-3-642-30825-3.**, vol. 9. Berlin, Heidelberg: Springer; 2012.

46. Peksa O, Skaloud P: **Do photobionts influence the ecology of lichens? A case study of environmental preferences in symbiotic green alga *Asterochloris* (Trebouxiophyceae)**. *Mol Ecol* 2011, **20**(18):3936-3948.

47. Yahr R, Vilgalys R, Depriest PT: **Strong fungal specificity and selectivity for algal symbionts in Florida scrub *Cladonia* lichens**. *Mol Ecol* 2004, **13**(11):3367-3378.

48. Cordeiro LMC, Sassaki GL, Iacomini M: **First report on polysaccharides of *Asterochloris* and their potential role in the lichen symbiosis**. *Int J Biol Macromol* 2007, **41**(2):193-197.

49. Koonin EV: **Evidence for a family of archaeal ATPases**. *Science* 1997, **275**(5305):1489-1490.

50. Schonknecht G, Chen WH, Ternes CM, Barbier GG, Shrestha RP, Stanke M, Brautigam A, Baker BJ, Banfield JF, Garavito RM *et al*: **Gene transfer from bacteria and archaea facilitated evolution of an extremophilic eukaryote**. *Science* 2013, **339**(6124):1207-1210.

51. Rocha EP: **Evolution. With a little help from prokaryotes**. *Science* 2013, **339**(6124):1154-1155.

52. Gruber G, Manimekalai MSS, Mayer F, Muller V: **ATP synthases from archaea: The beauty of a molecular motor**. *Bba-Bioenergetics* 2014, **1837**(6):940-952.

53. Hori K, Maruyama F, Fujisawa T, Togashi T, Yamamoto N, Seo M, Sato S, Yamada T, Mori H, Tajima N *et al*: ***Klebsormidium flaccidum* genome reveals primary factors for plant terrestrial adaptation**. *Nat Commun* 2014, **5**.

54. Banks JA, Nishiyama T, Hasebe M, Bowman JL, Gribskov M, dePamphilis C, Albert VA, Aono N, Aoyama T, Ambrose BA *et al*: **The *Selaginella* genome identifies genetic changes associated with the evolution of vascular plants**. *Science* 2011, **332**(6032):960-963.

55. Wu Q, Zou, YN.: **Arbuscular Mycorrhizal Fungi and Tolerance of Drought Stress in Plants**. In: *Arbuscular Mycorrhizas and Stress Tolerance of Plants.* Edited by Wu Q. Singapore: Springer; 2017.

56. Carniel FC, Gerdol M, Montagner A, Banchi E, De Moro G, Manfrin C, Muggia L, Pallavicini A, Tretiach M: **New features of desiccation tolerance in the lichen photobiont *Trebouxia gelatinosa* are revealed by a transcriptomic approach**. *Plant Mol Biol* 2016, **91**(3):319-339.

57. Martinoia E, Maeshima M, Neuhaus HE: **Vacuolar transporters and their essential role in plant metabolism**. *J Exp Bot* 2007, **58**(1):83-102.

58. Hermans C, Conn SJ, Chen J, Xiao Q, Verbruggen N: **An update on magnesium homeostasis mechanisms in plants**. *Metallomics : integrated biometal science* 2013, **5**(9):1170-1183.
