# Supplementary material for: The lichen symbiosis re-viewed through the genomes of Cladonia grayi and its algal partner Asterochloris glomerata
Source: BMC Genomics. 2019 Jul 23;20:605. doi: 10.1186/s12864-019-5629-x (PMC6652019; doi:10.1186/s12864-019-5629-x)
Supplement: Supplementary file 7 — Expanded and contracted families. (ZIP 220 kb) [file 12864_2019_5629_MOESM7_ESM.zip › Additional file 7/Additional file 7_2.The transportome of C. grayi and other fungi.pdf]

Distribution of genes for the main membrane transporter families in the symbiotic fungi *Cladonia grayi* and *Tuber melanosporum* compared to other ascomycetes with different life styles.

Family names are based on the Transport Classification Database: http://www.tcdb.org/. Lineage-specific gene gain and loss in the membrane transporter families have been estimated using CAFE (doi:10.1093/bioinformatics/btl097). Listed are 75 transporter families containing 14 expanded (e), 26 unchanged (n), 22 contracted (c) and 7 extinct (ex) families in the *Cladonia grayi* lineage. See Methods for more details. Significantly contracted *C. grayi* families are highlighted in color.

* indicates significant contraction ($P<0.05$) in the *C. grayi* branch.

Abbreviations: *ASPFU : Aspergillus_fumigatus, ASPNI : Aspergillus_nidulans, ASPOR : Aspergillus_oryzae, BOTCI : Botrytis_cinerea, CHAGL : Chaetomium_globosum, CLAGR : Cladonia grayi, FUSGR : Fusarium_graminearum, NEUCR : Neurospora_crassa, PYRTR : Pyrenophora_tritici-repentis, SCLSC : Sclerotinia_sclerotiorum, STANO : Stagonospora_nodorum, TUBME : Tuber_melanosporum*

[1]http://genome.jgi.doe.gov/Clagr2/Clagr2.home.html
[2]http://mycor.nancy.inra.fr/IMGC/TuberGenome
[3]http://www.broadinstitute.org/annotation/genome/neurospora/Home.html,
[4] http://www.broadinstitute.org/annotation/genome/chaetomium_globosum/Home.html
[5]http://www.broadinstitute.org/annotation/genome/botrytis_cinerea/Home.html,
[6]http://www.broadinstitute.org/annotation/genome/aspergillus_group/MultiHome.html,
[7]www.broadinstitute.org/annotation/genome/fusarium_graminearum/MultiHome.html
[8]http://genome.jgi-psf.org/Pyrtr1/Pyrtr1.home.html
[9]http://www.broadinstitute.org/annotation/genome/sclerotinia_sclerotiorum/MultiHome.html
[10]http://genome.jgi-psf.org/Stano1/Stano1.home.html

Transporter type Family (TC number)	*CLAGR*[1]	*TUBME*[2]	*NEUCR*[3]	*CHAGL*[4]	*BOTCI*[5]	*ASPNI*[6]	*ASPFU*[6]	*ASPOR*[6]	*FUSGR*[7]	*PYRTR*[8]	*SCLSC*[9]	*STANO*[10]	Changes in *C. grayi*
	Symbiont	Symbiont	Saprophyte	Saprophyte	Plant-pathogen	Saprophyte	Animal pathogen	Sapropyhte	Plant pathogen	Plant pathogen	Plant pathogen	Plant pathogen	
**ATP-Dependent**													
ABC (ATP-binding Cassette) (3.A.1.)	22	28	26	30	43	42	41	58	53	31	35	33	c*
F-ATPase (H+- or Na+-translocating F-type, V-type and A-type ATPase (3.A.2)	20	21	21	26	17	26	20	21	22	24	21	22	c
MPT (Mitochondrial Protein Translocase) (3.A.8)	21	20	17	21	18	25	25	19	25	26	25	27	c
P-ATPase (P-type ATPase) (3.A.3)	16	17	19	20	19	23	23	25	27	20	15	22	c*

Sec (General Secretory Pathway) (3.A.5)	5	7	7	7	6	9	7	8	9	10	9	10	c
**TOTAL PROTEINS (ATP-Dependent)**	**84**	**93**	**90**	**104**	**103**	**125**	**116**	**131**	**136**	**111**	**105**	**114**	c*
**Ion Channels**													
Amt (Ammonium Transporter) (1.A.11)	4	4	4	2	3	4	3	4	4	5	3	5	n
Annexin	2	1	1	3	1	3	3	2	3	2	1	2	n
Ctr (Copper Transporter) (1.A.56)	1	1	1	1	0	2	1	0	1	2	1	3	n
Mid1 (Yeast Stretch-Activated, Cation-Selective, Ca2+ Channel)	1	1	1	1	1	1	1	0	1	1	1	1	n
MIP (Major Intrinsic Protein) (1.A.8)	7	2	1	1	11	5	3	9	6	7	9	8	e
1.A.35 MIT (CorA Metal Ion Transporter)	0	0	1	0	0	0	0	0	0	0	0	0	n
1.MPP (Mitochondrial and Plastid Porin)	1	1	1	1	1	1	1	1	1	1	1	1	n
NSCC2 (Non-selective Cation Channel-2)	1	1	1	1	1	1	1	1	1	1	1	1	n
TRP-CC (Transient Receptor Potential Ca2+ Channel)	0	0	1	3	1	0	1	0	4	0	2	1	ex
VIC (Voltage-gated Ion Channel)	4	2	3	2	3	1	4	3	3	3	3	3	e
**TOTAL PROTEINS (Ion Channels)**	**21**	**13**	**15**	**15**	**22**	**18**	**18**	**20**	**24**	**22**	**22**	**25**	e
**Secondary Transporters**													
ACR3 (Arsenic Resistance-3) (2.A.59)	1	1	1	1	1	1	3	1	1	2	1	1	n
AE (Anion (2.A.31)	2	2	2	2	2	2	2	2	3	3	1	2	n
AEC (Auxin Efflux Carrier) (2.A.69)	0	2	2	0	3	2	0	0	0	0	0	0	ex
APC (Amino Acid-Polyamine-Organocation) (2.A.3)	47	19	26	28	49	71	62	96	82	42	47	55	c*
AAAP (Amino Acid/Auxin Permease)	11	4	7	8	9	14	15	24	27	10	9	14	c
ACT (Amino Acid/Choline Transporter)	21	5	4	3	19	24	27	38	23	16	19	21	c

LAT (L-type Amino Acid Transporter)	9	4	5	6	5	8	4	8	7	5	3	6	e
YAT (Yeast Amino Acid Transporter)	6	6	10	11	16	25	16	26	25	11	16	14	c*
CaCA (Ca2+:Cation Antiporter) (2.A.19)	9	5	8	9	9	6	7	10	9	7	9	9	e
CCC (Cation-Chloride Cotransporter) (2.A.30)	1	1	1	1	1	1	1	1	1	1	1	1	e
CDF (Cation Diffusion Facilitator) (2.A.4)	6	4	8	5	6	6	7	7	7	8	6	8	n
CHR (Chromate Ion Transporter) (2.A.51)	1	2	1	0	0	2	1	1	1	1	0	1	n
ClC (Chloride Carrier/Channel) (2.A.49)	4	3	3	3	3	3	3	3	4	4	3	4	e
CNT (Concentrative Nucleoside Transporter) (2.A.41)	1	1	1	1	0	1	1	1	1	1	0	1	n
CPA1 (Monovalent Cation:Proton Antiporter-1) (2.A.36)	4	2	3	4	5	6	4	4	5	5	4	6	n
CPA2 (Monovalent Cation:Proton Antiporter-2) (2.A.37)	2	1	2	1	2	2	3	3	2	2	3	3	n
DAACS (Dicarboxylate/Amino Acid:Cation (Na+ or H+) Symporter) (2.A.23)	0	0	0	1	0	1	0	0	1	1	0	1	ex
DASS (Divalent Anion:Na+ Symporter)(2.A.47)	1	1	1	1	1	1	1	1	1	1	1	1	n
DMT (Drug/Metabolite Transporter) (2.A.7)	11	11	13	10	10	9	11	9	13	11	11	10	e
ENT (Equilibrative Nucleoside Transporter)(2.A.57)	1	1	1	1	1	2	1	2	1	1	1	1	n
FNT (Formate-Nitrite Transporter) (2.A.44)	0	0	1	1	1	1	0	2	2	0	1	0	n
FP (Ferroportin) (2.A.100)	2	1	0	1	1	1	1	2	1	1	1	1	e
GPH (Glycoside-Pentoside-Hexuronide: Cation Symporter (2.A.2))	3	1	2	2	4	2	2	2	3	3	3	2	e

GUP (Glycerol Uptake)(2.A.50)	1	1	0	1	0	1	1	1	1	1	1	1	n
KUP (K+ Uptake Permease) (2.A.72)	2	0	1	0	1	0	0	1	1	1	1	1	e
LCT (Lysosomal Cystine Transporter) (2.A.43)	2	3	2	2	3	3	4	2	3	2	2	1	c
MC (Mitochondrial Carrier) (2.A.29)	33	34	34	31	38	36	37	36	34	36	34	38	c
MFS (Major Facilitator Superfamily) (2.A.1)	177	85	154	191	279	382	300	s	362	257	207	359	c*
The Anion:Cation Symporter (ACS) Family (2.A.1.14)	14	16	27	28	35	81	52	104	82	45	30	85	c*
MOP (Multidrug/Oligosaccharidyl-lipid/Polysaccharide) Flippase) (2.A.66)	3	3	4	1	1	2	3	3	6	4	2	3	n
MTC (Mitochondrial Tricarboxylate Carrier) (2.A.54)	1	1	1	1	1	1	1	2	1	1	1	1	n
NCS1 (Nucleobase:Cation Symporter-1) (2.A.39)	10	1	3	4	7	11	6	11	7	11	9	15	e
NCS2 (Nucleobase:Cation Symporter-2) (2.A.40)	0	3	1	1	1	2	1	1	1	2	1	1	ex
Nramp (Metal Ion (Mn2+-iron) Transporter)(2.A.55)	1	0	2	1	1	1	1	1	1	1	2	0	n
NSS (Neurotransmitter:Sodium Symporter) (2.A.22)	0	0	0	0	0	2	0	2	3	3	0	2	ex
OPT (Oligopeptide Transporter) (2.A.67)	3	3	11	9	12	6	8	9	11	2	12	4	c*
Oxa1 (Cytochrome Oxidase Biogenesis) (2.A.9)	1	1	1	1	1	1	1	1	1	1	1	1	n
PiT (Inorganic Phosphate Transporter) (2.A.20)	0	1	1	1	1	4	3	4	4	2	1	3	ex
POT (Proton-dependent Oligopeptide Transporter)(2.A.17)	1	3	2	3	3	4	7	16	4	8	5	10	c*

RND (Resistance-Nodulation-Cell Division) (2.A.6)	1	1	1	1	1	1	1	1	1	1	1	1	n
SSS (Solute:Sodium Symporter) (2.A.21)	2	2	2	1	3	4	3	3	2	4	1	3	c
SulP (Sulfate Permease) (2.A.52)	2	4	4	4	4	2	2	2	5	5	3	5	n
TDT (Tellurite-resistance/Dicarboxylate Transporter) (2.A.16)	3	0	2	1	4	5	7	6	8	7	2	6	c
Trk(K^+ Transporter)(2.A.38)	2	2	2	4	2	3	3	5	4	3	2	4	c
YaaH (ATO) (2.A.96)	4	0	1	2	3	5	3	4	5	4	2	4	n
ZIP (Zinc (Zn^{2+})-Iron (Fe^{2+}) Permease)(2.A.5)	3	7	7	9	7	7	7	9	9	7	6	7	c
**TOTAL PROTEINS (Secondary Transporter)**	**348**	**213**	**312**	**341**	**472**	**603**	**509**	**267**	**612**	**457**	**389**	**577**	c*
**Incompletely Characterized Transport System**													
ATP-E (ATP Exporter) (9.A.6)	2	1	1	1	1	1	1	1	1	1	1	1	e
ILT (Iron/Lead Transporter) (9.A.10)	2	2	2	2	2	0	2	2	3	2	2	2	e
MHP (Metal Homeostasis Protein) (9.B.1)	0	1	1	0	1	1	1	1	1	1	1	1	ex
SHP (Stress-Induced Hydrophobic Peptide) (9.B.12)	1	0	0	1	0	2	2	0	2	2	3	3	c
**TOTAL PROTEINS (Incompletely Characterized)**	5	4	4	4	4	4	6	4	7	6	7	7	n
**TOTAL TRANSPORTER PROTEINS**	**458**	**323**	**421**	**464**	**601**	**750**	**649**	**422**	**779**	**596**	**523**	**723**	c*
