# Supplementary material for: The lichen symbiosis re-viewed through the genomes of Cladonia grayi and its algal partner Asterochloris glomerata
Source: BMC Genomics. 2019 Jul 23;20:605. doi: 10.1186/s12864-019-5629-x (PMC6652019; doi:10.1186/s12864-019-5629-x)
Supplement: Supplementary file 8 — Signal transduction diversification. (ZIP 3420 kb) [file 12864_2019_5629_MOESM8_ESM.zip › Additional file 8/Additional file 8_1.Fungal and algal signal transduction diversification.docx]

**Additional file 8_1**

**Fungal and algal signal transduction protein diversification**

**A.** The largest fungal family of signal transduction components (Additional file 8_3) contains 121 PTH11-type receptors, unique to filamentous ascomycetes and some connected to pathogenicity in *Magnaporthe oryzae*, where they were first identified as a 61-member family [1]. Unfortunately, their functions are poorly understood. **B.** Also expanded are families of well-known components of MAPK pathways: G-protein α subunits, RGS proteins (Regulators of G-protein Signaling), and dual specificity phosphatases, with 8, 8, and 11 members respectively (Additional file 8_3). A phylogenetic analysis of the eight Gα subunits in *C. grayi* (Fig. 13) reveals that three correspond to the standard Gα subunits present in all fungi (MAG A, MAG B and MAG C), while the other five appear to be divergent paralogs of MAG C (CLAGR_011186-RA), possibly adapted to specific symbiotic functions. Proteins corresponding to the standard and the five new Gα subunits are also in the genome of the lichen fungus *Endocarpon pusillum* [2] (Armaleo, data not shown), which belongs to a different class (Eurotiomycetes) within the Pezizomycotina. Gα protein family expansion might be thus widespread among lichens, a possibility to be investigated. **C.** Overexpression in coculture (Additional file 8_2) points to several fungal signal transduction genes of potential symbiotic significance, most notably the original MAG C paralog CLAGR_011186-RA; CLAGR_002910-RA, an RGS family member; CLAGR_002710-RA, a dual specificity phosphatase; and CLAGR_000113-RA, a putative Ca^++^ transporter mentioned in Additional file 6. **D.** G-proteins are generally present in plants, although GPCRs are absent [3, 4]. Chlorophyta, however, lack both GPCRs and G-proteins [5], and *Asterochloris* is no exception (Additional file 8_4). Regarding *Asterochloris*-specific expansions of signal transduction protein families, we relate them only to two other Trebouxiophyceae, *Chlorella* NC64A and *Coccomyxa* C169. Data from other sequenced Chlorophyta and *Arabidopsis* are included in the table for completeness but are deemed less informative with regard to the lichen symbiosis, given the large phylogenetic distances, genome size differences and lifestyle differences. The *Asterochloris* expansions involve a more diverse set of families than in *Cladonia,* and the expanded components can be broadly grouped as likely parts of the same signal transduction processes (Additional file 8_4): protein kinases and phosphatases; Ras-like GTPases and GTPase regulator proteins; 2-component system receptors and regulators; light sensors (Bacteriorhodopsin-like); adenylate cyclases and cyclic nucleotide binding proteins. This apparent correlation of lichenization with signal transduction diversification is another hallmark of an extremely versatile photobiont able to live independently occasionally [6, 7], engage in varied fungus-alga interactions (Additional file 7_1), and contribute to the adaptability of lichens to wide ranges of ecological conditions.

**References**

1. Kulkarni RD, Thon MR, Pan HQ, Dean RA: **Novel G-protein-coupled receptor-like proteins in the plant pathogenic fungus *Magnaporthe grisea***. *Genome biology* 2005, **6**(3).

2. Wang YY, Liu B, Zhang XY, Zhou QM, Zhang T, Li H, Yu YF, Zhang XL, Hao XY, Wang M *et al*: **Genome characteristics reveal the impact of lichenization on lichen-forming fungus *Endocarpon pusillum* Hedwig (Verrucariales, Ascomycota)**. *Bmc Genomics* 2014, **15**:34.

3. Urano D, Chen JG, Botella JR, Jones AM: **Heterotrimeric G protein signalling in the plant kingdom**. *Open Biol* 2013, **3**.

4. Hackenberg D, Pandey S: **Heterotrimeric G-proteins in green algae. An early innovation in the evolution of the plant lineage**. *Plant Signal Behav* 2014, **9**(4):e28457.

5. Urano D, Jones JC, Wang H, Matthews M, Bradford W, Bennetzen JL, Jones AM: **G Protein Activation without a GEF in the Plant Kingdom**. *Plos Genet* 2012, **8**(6):e1002756.

6. Ahmadjian V: **The Lichen Alga *Trebouxia* - Does It Occur Free-Living?** *Plant Syst Evol* 1988, **158**(2-4):243-247.

7. Sanders WB: **Observing microscopic phases of lichen life cycles on transparent substrata placed in situ**. *Lichenologist* 2005, **37**:373-382.
