# Supplementary material for: The lichen symbiosis re-viewed through the genomes of Cladonia grayi and its algal partner Asterochloris glomerata
Source: BMC Genomics. 2019 Jul 23;20:605. doi: 10.1186/s12864-019-5629-x (PMC6652019; doi:10.1186/s12864-019-5629-x)
Supplement: Supplementary file 10 — Carbonic anhydrases in A. glomerata. (DOCX 217 kb) [file 12864_2019_5629_MOESM10_ESM.docx]

**Additional file 10**

**Carbonic anhydrases in *A. glomerata***

In photosynthetic organisms, carbonic anhydrases (CAs) concentrate CO_2_ near ribulose-1-5-biphosphate carboxylase (Rubisco), the enzyme responsible for most of the inorganic carbon entering the biosphere. Three families of CAs, α, β, and γ, originated independently and are widespread across all kingdoms of life [1] performing the same reaction in a variety of contexts. The alga *Chlamydomonas* *reinhardtii* has been a major cellular and molecular model for the roles played in the carbon concentrating mechanism (CCM) by the different CAs found in mitochondria, thylakoids, cytoplasm and periplasm of Chlorophyta [2, 3]. *Asterochloris* has one α- and four β-type CAs (below Figure, panel A). Panels B and C depict gene phylogenies of α and β CAs from four chlorophycean taxa. The α CA of *Asterochloris* (Aste_gl_8844) clusters with *CAH3* of *Chlamydomonas*. *CAH3* has a chloroplast targeting sequence and has been localized to the thylakoid lumen associated with the pyrenoid [2, 3]; *CAH3* is thus likely to deliver CO_2_ directly to Rubisco, the majority protein in the pyrenoid. Since Aste_gl_8844 also has a predicted chloroplast targeting sequence, we predict that Aste_gl_8844 is localized in the pyrenoid. CA11032 from *Chlorella* clusters with *CAH3* as well, but none of the *Coccomyxa* CAs do (below Figure, panel B). This corroborates a known distinction between *Coccomyxa*, in which both lichenized [4] and free-living [5] species lack pyrenoids, and *Chlamydomonas*, *Chlorella* and *Asterochloris*, that have pyrenoids [2, 3, 6-9]. Five of the six β-type CAs from *Chlamydomonas* have N-terminal target sequences that localize them to organelles or extracellularly, and only *CAH9* lacks a target sequence and likely operates in the cytoplasm [2, 3]. Most of the β CAs from the other algae in below Figure, panel C, lack target sequences (with the possible exception of *Chlorella* 12412, whose N terminal region is incomplete) and are presumably cytoplasmic. All four β CAs in *Asterochloris* cluster in a distinct subclade with cytoplasmic *CAH9* of *Chlamydomonas* (below Figure, panel C). Taken together, these data suggest that *Asterochloris* has one α CA functioning in the pyrenoid like *Chlamydomonas* and *Chlorella*, but that it has an expanded specific subclass of cytoplasmic β CAs. Detailed analysis of each CA in the lichen and in culture should verify these suggestions and help clarify the specific significance of the *Asterochloris* CAs for the symbiosis.

# Distribution and phylograms of carbonic anhydrases (CAs)

# CAs were sampled in *A. glomerata* and three other Chlorophyta. (Protein data: from each Chlorophyta genome at [10]; from *Arabidopsis* at [11]; (phylograms: PhyML, 100 bootstraps). A: Distribution of α, β, and γ CAs and pyrenoids among the four taxa. B: Phylogram of α CAs. The numbers next to the species acronyms are protein identifiers. Bootstrap values are next to each node. The CA from *Asterochloris* is highlighted red. C: Phylogram of β CAs. Same layout as in B. Notice that in panel B Aste_gl_8844 clusters with thylakoid-CAH3, and in panel C the four CAs from *Asterochloris* cluster with cytoplasmic-CAH9. Species acronyms: Aste_gl, *Asterochloris glomerata*; Cocc_sp, *Coccomyxa subellipsoidea* C-169; Chlo_va, *Chlorella variabilis* NC64A; Chla_re, *Chlamydomonas reinhardtii*; Arab_th, *Arabidopsis thaliana.*

# References

1. Banerjee S, Deshpande PA: **On origin and evolution of carbonic anhydrase isozymes: A phylogenetic analysis from whole-enzyme to active site**. *Comput Biol Chem* 2016, **61**:121-129.

2. Meyer M, Griffiths H: **Origins and diversity of eukaryotic CO2-concentrating mechanisms: lessons for the future**. *J Exp Bot* 2013, **64**(3):769-786.

3. Moroney JV, Ma YB, Frey WD, Fusilier KA, Pham TT, Simms TA, DiMario RJ, Yang J, Mukherjee B: **The carbonic anhydrase isoforms of *Chlamydomonas reinhardtii*: intracellular location, expression, and physiological roles**. *Photosynthesis research* 2011, **109**(1-3):133-149.

4. Palmqvist K, Ogren E, Lernmark U: **The Co2-Concentrating Mechanism Is Absent in the Green Alga *Coccomyxa* - a Comparative-Study of Photosynthetic Co2 and Light Responses of *Coccomyxa*, *Chlamydomonas reinhardtii* and Barley Protoplasts**. *Plant Cell Environ* 1994, **17**(1):65-72.

5. Blanc G, Agarkova I, Grimwood J, Kuo A, Brueggeman A, Dunigan DD, Gurnon J, Ladunga I, Lindquist E, Lucas S *et al*: **The genome of the polar eukaryotic microalga *Coccomyxa subellipsoidea* reveals traits of cold adaptation**. *Genome biology* 2012, **13**(5):R39.

6. Blanc G, Duncan G, Agarkova I, Borodovsky M, Gurnon J, Kuo A, Lindquist E, Lucas S, Pangilinan J, Polle J *et al*: **The *Chlorella variabilis* NC64A Genome Reveals Adaptation to Photosymbiosis, Coevolution with Viruses, and Cryptic Sex**. *Plant Cell* 2010, **22**(9):2943-2955.

7. Palmqvist K: **Carbon economy in lichens**. *New Phytologist* 2000, **148**(1):11-36.

8. Nĕmcová Y KT: **Cell wall development, microfibril and pyrenoid structure in type strains of *Chlorella vulgaris*, *C. kessleri*, *C. sorokiniana* compared with *C. luteoviridis* (Trebouxiophyceae, Chlorophyta)**. *Algological Studies* 2000, **100**:95-105.

9. Moya P, Skaloud P, Chiva S, Garcia-Breijo FJ, Reig-Arminana J, Vancurova L, Barreno E: **Molecular phylogeny and ultrastructure of the lichen microalga *Asterochloris mediterranea* sp nov from Mediterranean and Canary Islands ecosystems**. *Int J Syst Evol Micr* 2015, **65**:1838-1854.

10. **Joint Genome Institute Portal.** Available from: http://genome.jgi.doe.gov/. Accessed 15 June 2016.

11. **The Arabidopsis Information Resource.** Avilable from: <https://www.arabidopsis.org/>. Accessed 3 December 2017.
