# Supplementary material for: The lichen symbiosis re-viewed through the genomes of Cladonia grayi and its algal partner Asterochloris glomerata
Source: BMC Genomics. 2019 Jul 23;20:605. doi: 10.1186/s12864-019-5629-x (PMC6652019; doi:10.1186/s12864-019-5629-x)
Supplement: Supplementary file 11 — Slow- and fast-evolving proteins. (ZIP 104 kb) [file 12864_2019_5629_MOESM11_ESM.zip › Additional file 11/Additional file 11_1.Slow- and fast-evolving proteins.docx]

**Additional file 11_1**

**Slow- and fast-evolving proteins**

**Slow-evolvers and other double-scoring proteins in the mycobiont**

A double-scoring slow-evolver of particular interest is CLAGR_002836-RA due to its predicted function resembling that of Aster-03695, an algal gene highlighted in Additional file 6_1 and Table 3. CLAGR_002836-RA encodes a putative calcium-binding mechanosensitive ion channel from a family that includes the *S. pombe* osmosensors *Msy2* and *Msy1* [1]. Osmotically elicited changes in membrane structure or cell volume can affect ion flux through such channels to modulate fungal responses to various stimuli [2], like osmotic stress [1], suggesting that this channel may also operate in the context of the lichen’s exposure to rapid desiccation/rehydration cycles. Besides the 38 double-scoring slow-evolvers discussed in the Slow-evolving proteins and anti-stress strategies in the mycobiont section, Additional file 11_2 also lists slow-evolving mycobiont proteins that scored double in other ways, including being induced in the reconstitution experiment or belonging to contracted *C. grayi* protein families. Among these alternative double-scorers, two groups stand out due to their homogeneity: eighteen membrane transporters and seven aldehyde dehydrogenases (ALDHs). Most of the eighteen double-scoring transporters belong to the Major Facilitator Superfamily (MFS) of membrane transporters that carry a variety of small substrates in and out of cells and cell compartments [3, 4]. These transporters are a subset of the overall contracted transportome (Additional file 7). The seven ALDHs scoring as slow-evolvers by MCL also belong to a contracted protein family (fam_20) reduced from 18 to 10 members in *C. grayi* (Additional file 7). The slow evolution and concomitant pruning of the fam_20 tree suggest that in *C. grayi* 44% of the members became unnecessary and seven of the remaining ten could be under purifying selection. ALDHs are ubiquitous enzymes that oxidize aldehydes to carboxylic acids by producing NAD(P)H [5]. In addition to being involved in intermediary metabolism, many ALDHs play major roles in protecting cells from oxidative damage either by neutralizing toxic aldehydes derived from the peroxidation of lipids by reactive oxygen species [6, 7] or by synthesizing betaine, an alternative nitrogen source [8] that also plays a large role in protecting proteins and membranes from oxidative damage [9]. Thus, the evolution of these lichen ALDHs might have also been molded by the mycobiont's adaptation to oxidative stress from rapid dehydration/rehydration and/or from interaction with the oxygen-producing alga [10].

**Fast-evolving proteins in the mycobiont**

As discussed in the Search for symbiosis-specific genes III: Proteins with anomalous rates of evolution section, we view the fast-evolvers as representing relatively recent changes. Among the eleven proteins defined as fast-evolvers by at least two criteria, five satisfied all three (Additional file 11_2). The presumed functions of the eleven proteins include signal transduction (CLAGR_001337-RA, CLAGR_004875-RA, CLAGR_000913-RA), membrane trafficking (CLAGR_000379-RA, CLAGR_007666-RA), and stress protection. We place in the latter category CLAGR_008836-RA and CLAGR_000199-RA, which encode respectively superoxide dismutase, a universal and well-known defense against oxidative stress, and trehalose synthase. Trehalose, a disaccharide ubiquitous across all kingdoms of life, has a variety of roles [11], and prominent among them is protection of membranes and proteins during dehydration/rehydration [12]. Increases in intracellular threhalose concentration raise desiccation tolerance in *S. cerevisiae* several thousand-fold [13]. Thus, trehalose and betaine may perform similar roles in the lichen fungus. Most other proteins in Additional file 11_2 include the induced members of mycobiont-expanded families discussed in Additional file 7.

**Slow- and fast- evolving proteins in the photobiont**

The evolutionary rate approach did not produce clear results for the alga. Only three *A. glomerata* proteins were identified as slow-evolvers by two methods. Six more were slow-evolvers by one method and induced in coculture (Additional file 11_2). One of the nine is a DNA-binding protein. Two are kinesin motor domains. One is a clathrin-linked adaptor protein involved in membrane receptor recycling which, together with five kinases, suggests that most of the algal slow-evolvers may be involved in signal transduction. Seven *A. glomerata* proteins were identified as fast-evolvers by at least two methods (Additional file 11_2), and include Aster-01625, the coculture-induced kinesin-domain protein mentioned in Additional file 6. The seven proteins represent a heterogeneous group with no clear overall trend. The one protein identified as fast-evolving by all three methods is the ATP-grasp domain of phosphoribosylglycinamide synthetase, which catalyzes an early step in purine biosynthesis. Most other proteins in Additional file 11 include the induced members of expanded families discussed in Additional file 7.

**References**

1. Nakayama Y, Yoshimura K, Iida H: **Organellar mechanosensitive channels in fission yeast regulate the hypo-osmotic shock response**. *Nat Commun* 2012, **3**.

2. Lew RR, Abbas Z, Anderca MI, Free SJ: **Phenotype of a mechanosensitive channel mutant, mid-1, in a filamentous fungus, *Neurospora crassa***. *Eukaryot Cell* 2008, **7**(4):647-655.

3. Coleman JJ, Mylonakis E: **Efflux in Fungi: La Piece de Resistance**. *Plos Pathog* 2009, **5**(6):e1000486.

4. Stergiopoulos I, Zwiers LH, De Waard MA: **Secretion of natural and synthetic toxic compounds from filamentous fungi by membrane transporters of the ATP-binding cassette and major facilitator superfamily**. *Eur J Plant Pathol* 2002, **108**(7):719-734.

5. Perozich J, Nicholas H, Wang BC, Lindahl R, Hempel J: **Relationships within the aldehyde dehydrogenase extended family**. *Protein Sci* 1999, **8**(1):137-146.

6. Sunkar R, Bartels D, Kirch HH: **Overexpression of a stress-inducible aldehyde dehydrogenase gene from *Arabidopsis thaliana* in transgenic plants improves stress tolerance**. *Plant Journal* 2003, **35**(4):452-464.

7. Asiimwe T, Krause K, Schlunk I, Kothe E: **Modulation of ethanol stress tolerance by aldehyde dehydrogenase in the mycorrhizal fungus *Tricholoma vaccinum***. *Mycorrhiza* 2012, **22**(6):471-484.

8. Lambou K, Pennati A, Valsecchi I, Tada R, Sherman S, Sato H, Beau R, Gadda G, Latge JP: **Pathway of Glycine Betaine Biosynthesis in *Aspergillus fumigatus***. *Eukaryot Cell* 2013, **12**(6):853-863.

9. Chen THH, Murata N: **Glycinebetaine protects plants against abiotic stress: mechanisms and biotechnological applications**. *Plant Cell Environ* 2011, **34**(1):1-20.

10. Kranner I, Cram WJ, Zorn M, Wornik S, Yoshimura I, Stabentheiner E, Pfeifhofer HW: **Antioxidants and photoprotection in a lichen as compared with its isolated symbiotic partners**. *P Natl Acad Sci USA* 2005, **102**(8):3141-3146.

11. Tournu H, Fiori A, Van Dijck P: **Relevance of Trehalose in Pathogenicity: Some General Rules, Yet Many Exceptions**. *Plos Pathog* 2013, **9**(8):e1003447.

12. Elbein AD, Pan YT, Pastuszak I, Carroll D: **New insights on trehalose: a multifunctional molecule**. *Glycobiology* 2003, **13**(4):17R-27R.

13. Tapia H, Young L, Fox D, Bertozzi CR, Koshland D: **Increasing intracellular trehalose is sufficient to confer desiccation tolerance to *Saccharomyces cerevisiae***. *P Natl Acad Sci USA* 2015, **112**(19):6122-6127.
