# Supplementary material for: The lichen symbiosis re-viewed through the genomes of Cladonia grayi and its algal partner Asterochloris glomerata
Source: BMC Genomics. 2019 Jul 23;20:605. doi: 10.1186/s12864-019-5629-x (PMC6652019; doi:10.1186/s12864-019-5629-x)
Supplement: Supplementary file 12 — Locations and lengths of nuclear rDNA introns in Cladonia grayi. (DOCX 130 kb) [file 12864_2019_5629_MOESM12_ESM.docx]

**Additional file 12**

# Locations and lengths of nuclear rDNA introns in *Cladonia grayi*

# Black lines represent the sequence of the small (top) and the large (bottom) rDNA subunits. The numbers below the lines correspond to the reference rRNA sequence of *Saccharomyces cerevisiae* (measured from the start of the mature SSU or LSU rRNA [1]) and indicate the insertion sites of all currently known *C. grayi* rDNA introns. The red bars and numbers indicate intron length. Varying subsets of these introns are present in different *C. grayi* isolates, and the sequence of a given intron can vary slightly from isolate to isolate.

**References**

1. Stanford University DoG. ***Saccharomyces* Genome Database.** Avaliable from: http://www.yeastgenome.org/. Accessed July 2 2017.
