# Supplementary material for: The lichen symbiosis re-viewed through the genomes of Cladonia grayi and its algal partner Asterochloris glomerata
Source: BMC Genomics. 2019 Jul 23;20:605. doi: 10.1186/s12864-019-5629-x (PMC6652019; doi:10.1186/s12864-019-5629-x)
Supplement: Supplementary file 13 — Likelihood-based heterotachy detection method. (DOCX 22 kb) [file 12864_2019_5629_MOESM13_ESM.docx]

**Additional file 13**

**Likelihood-based heterotachy detection method**

The method is a likelihood-based phylogenetic approach for detecting lineage-specific radical changes in substitution rates of a certain locus relative to all other loci. I.e., it is meant to detect accelerations and decelerations in substitution rates, which means changes in the intensity of purifying selection. The method can be applied to nucleotide and amino acid sequences, and hence it is applicable to both protein-coding as well as non-coding DNA. If it is applied to nucleotide sequences it uses by default the six parameter general time reversible (GTR) model [1], and if it is applied to amino acid sequences it uses by default the JTT substitution matrix [2]. However, other nucleotide and amino acid GTR models can alternatively be used. The data input for the model are a multi-locus multiple sequence alignment (MSA), which we denote as *D_l_* , and a corresponding phylogenetic tree, the topology of which is denoted as $T$. The MSA is typically constructed for each individual locus interpedently, yet the phylogenetic tree topology corresponds to all loci. Our null assumption is that the selective constraints that each locus is subjected to are shared across the entire phylogeny, i.e., homotachy. This is captured by assuming that all loci share the same branch lengths of the phylogenetic tree. This null model, however, accounts for the possibility that different loci may be subjected to different phylogeny-wide selective constraints by allowing a locus-specific rate parameter. This is equivalent to assuming that the branch lengths of different loci are proportional to one another by a scaling factor [3, 4]. This among-loci-rate-variation is captured by assuming a global locus-specific evolutionary rate *γ* (or equivalently a locus-specific branch-length scaling factor), which is treated as a random variable sampled independently for each locus from a gamma distribution Γ(*α_γ_,β_γ_*) approximated by *C^γ^* discrete rate categories (as in [5] for modelling among-sites-rates-variation). The distribution of *γ* is restricted to have mean of one, which is facilitated by equating a*_γ_* and *β_γ_* thereby avoiding the confounding effect between evolutionary rates and divergence times [6]. Thus, the global locus rate is obtained by estimating a single parameter, a*_γ_* , which determines the shape of the distribution of *γ*. In addition to the global locus rate we further assume that each site within each locus *l* evolves under a substitution rate *λ* sampled from a locus-specific site-specific gamma distribution Γ(*α_γ_,β_γ_*), approximated by *C^λ^* discrete rate categories. Similar to the global locus rate distribution, *α_λ_* and *β_λ_* are also equated and thus for each locus we estimate an *α_λ_* parameter. In case a mechanistic GTR model is used, for each specific locus we additionally estimate the set of parameters of that model, which we denote *θ_l_*. Finally, we also estimate the set of tree branch lengths common to all loci, which we denote *τ* .

The likelihood function of this null model is therefore:

$$L=\prod_{l\in loci} L_{l}=\prod_{l\in loci} \left[\sum_{\gamma\in C^{\gamma}} P(\gamma)P(D_{l}|T,\tau,\theta_{l},\gamma)\right]$$

Where

$$P(D_{l}|T,\tau,\theta_{l},\gamma)=\prod_{s\in l} \left[\sum_{\lambda\in C^{\lambda}} P(\lambda)P(D_{l_{s}}|T,\tau,\theta_{l},\gamma,\lambda)\right]$$

is the likelihood of locus *l* under global locus rate *γ*, where *s* denotes site.

Parameters are estimated such that they maximize the likelihood function, which is computed using the pruning algorithm [6]. To test for violations of our null assumptions, i.e. when the selective constraints a locus is subjected to are not shared across the entire phylogeny, suggesting heterotachy, we re-maximize the likelihood of the specific locus where all fitted null-model parameters are held fixed and only the length of the branch in the lineage where heterotachy is suspected is re-estimated. If the re-estimated maximum likelihood shows a significant improvement over that computed for this locus under the null model, we infer that this locus experienced a radical change in its selective regime in the specified lineage. If the re-estimated branch length is longer than that estimated under the null model this radical change is inferred as acceleration, and if it is shorter this radical change is inferred as deceleration. The statistical significance of likelihood improvement is computed empirically by simulating multi-locus data under the null assumptions and testing for violations in a specified lineage. This produces a distribution of delta likelihoods. A P-value for a real data test is the fraction of empirical delta likelihoods that are greater or equal to the real-data delta likelihood between the alternative and null models.

**References**

1. Rodriguez F, Oliver JL, Marin A, Medina JR: **The General Stochastic-Model of Nucleotide Substitution**. *J Theor Biol* 1990, **142**(4):485-501.

2. Jones DT, Taylor WR, Thornton JM: **The Rapid Generation of Mutation Data Matrices from Protein Sequences**. *Comput Appl Biosci* 1992, **8**(3):275-282.

3. Yang ZH: **Maximum-likelihood models for combined analyses of multiple sequence data**. *J Mol Evol* 1996, **42**(5):587-596.

4. Pupko T, Huchon D, Cao Y, Okada N, Hasegawa M: **Combining multiple data sets in a likelihood analysis: Which models are the best?** *Molecular biology and evolution* 2002, **19**(12):2294-2307.

5. Yang ZH: **Maximum-Likelihood Phylogenetic Estimation from DNA-Sequences with Variable Rates over Sites - Approximate Methods**. *J Mol Evol* 1994, **39**(3):306-314.

6. Felsenstein J: **Evolutionary Trees from DNA-Sequences - a Maximum-Likelihood Approach**. *J Mol Evol* 1981, **17**(6):368-376.
